# Genetics of flight in spongy moths (*Lymantria dispar* ssp.): functionally integrated profiling of a complex invasive trait

**DOI:** 10.1186/s12864-023-09936-8

**Published:** 2024-05-31

**Authors:** Gwylim S. Blackburn, Christopher I. Keeling, Julien Prunier, Melody A. Keena, Catherine Béliveau, Richard Hamelin, Nathan P. Havill, Francois Olivier Hebert, Roger C. Levesque, Michel Cusson, Ilga Porth

**Affiliations:** 1 Natural Resources Canada, Pacific Forestry Centre, Canadian Forest Service, 506 Burnside Road West, Victoria, BC V8Z 1M5 Canada; 2 Natural Resources Canada, Laurentian Forestry Centre, Canadian Forest Service, 1055 Rue du PEPS, Quebec City, Québec G1V 4C7 Canada; 3 Department of Wood and Forest Sciences, Laval University, 1030 Avenue de La Médecine, Québec, QC G1V 0A6 Canada; 4 Institute of Integrative Biology and Systems, Laval University, Québec, QC Canada; 5 United States Department of Agriculture, Northern Research Station, Forest Service, 51 Mill Pond Road, Hamden, CT 06514 USA; 6 Forest Sciences Centre, University of British Columbia, 2424 Main Mall, Vancouver, BC 3032V6T 1Z4 Canada; 7 Department of Biochemistry, Microbiology, and Bioinformatics, Laval University, Québec, QC G1V 0A6 Canada; 8 Centre for Forest Research, Laval University, 2405 Rue de La Terrasse, Québec, QC G1V 0A6 Canada

**Keywords:** GWAS, Inbred lines, Transcriptomics, Biological invasion, Biosurveillance, Spongy moth

## Abstract

**Background:**

Flight can drastically enhance dispersal capacity and is a key trait defining the potential of exotic insect species to spread and invade new habitats. The phytophagous European spongy moths (ESM, *Lymantria dispar dispar*) and Asian spongy moths (ASM; a multi–species group represented here by *L. d. asiatica and L. d. japonica*), are globally invasive species that vary in adult female flight capability—female ASM are typically flight capable, whereas female ESM are typically flightless. Genetic markers of flight capability would supply a powerful tool for flight profiling of these species at any intercepted life stage. To assess the functional complexity of spongy moth flight and to identify potential markers of flight capability, we used multiple genetic approaches aimed at capturing complementary signals of putative flight–relevant genetic divergence between ESM and ASM: reduced representation genome–wide association studies, whole genome sequence comparisons, and developmental transcriptomics. We then judged the candidacy of flight–associated genes through functional analyses aimed at addressing the proximate demands of flight and salient features of the ecological context of spongy moth flight evolution.

**Results:**

Candidate gene sets were typically non–overlapping across different genetic approaches, with only nine gene annotations shared between any pair of approaches. We detected an array of flight–relevant functional themes across gene sets that collectively suggest divergence in flight capability between European and Asian spongy moth lineages has coincided with evolutionary differentiation in multiple aspects of flight development, execution, and surrounding life history. Overall, our results indicate that spongy moth flight evolution has shaped or been influenced by a large and functionally broad network of traits.

**Conclusions:**

Our study identified a suite of flight–associated genes in spongy moths suited to exploration of the genetic architecture and evolution of flight, or validation for flight profiling purposes. This work illustrates how complementary genetic approaches combined with phenotypically targeted functional analyses can help to characterize genetically complex traits.

**Supplementary Information:**

The online version contains supplementary material available at 10.1186/s12864-023-09936-8.

## Background

The spread of invasive alien species (IAS) around the world has accelerated over the past century [1], with devastating consequences for natural ecosystems [2–7] and global commerce [7–9]. These impacts have prompted increased efforts to strengthen detection and intervention of IAS to limit their movement from place of origin, dispersal, establishment in novel habitats, and population expansion [10–15]. Species–specific traits that strongly affect the risk or extent of biological invasion (“invasive traits”) [16] offer potentially powerful tools for this purpose because they present a means to strategically confront individual IAS on multiple aspects of the invasion process: profiling intercepted specimens in terms of their origin or invasive potential; identifying habitat features or geographic regions that are most vulnerable to invasion, and; directly targeting invasive traits to limit IAS ecological success [17–20].

**Fig. 1:**
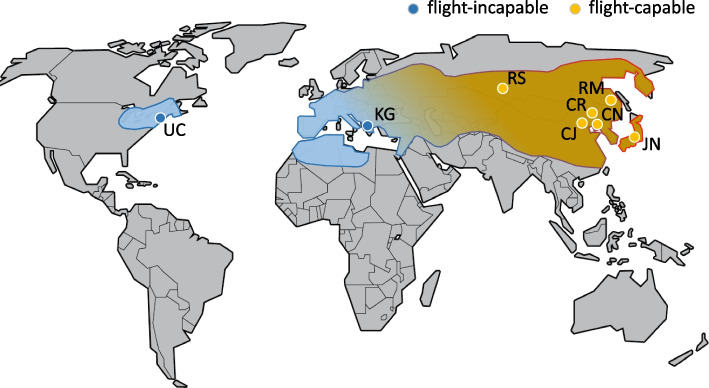
Spongy moth global range and sample colony locations. The outline represents the known global range of spongy moths *Lymantria dispar* ssp. Coloration approximates the global transition in reported adult female flight capability from *L. d. dispar* (blue: typically flight–incapable) to *L. d. asiatica*, *L. d. japonica* and other members of Asian spongy moth (orange: typically flight–capable) across a broad hybrid zone in Europe. Strain locations are shown with two–letter codes

In this study, we assessed the potential to apply these management tools to spongy moths, *Lymantria dispar* ssp., through the identification of candidate genes for female flight capability. Our approach was to compare two members of the flight–capable Asian spongy moth subspecies group, *L. d. asiatica* and *L. d. japonica* (for shorthand, ASM), with a sister subspecies, the European spongy moth (ESM, *L. d. dispar*), which features typically flightless adult females (Fig. 1) [21–23]. ESM and ASM are among the most destructive IAS globally [24]. Larvae of both forms are capable of feeding on a wide array of deciduous and coniferous host species around the world [22, 25–29], and population outbreaks can lead to substantial losses in natural, commercial, or urban forest stands [27]. ESM has spread across eastern North America since its accidental introduction from Europe in 1869, in part through natural dispersal of larvae but also via incidental transport of egg masses on motor vehicles or their cargo. ASM is not established in North America but is frequently detected on shipping materials or vessels at coastal ports of entry, and occasionally inland from those sites [30]. Importantly, differential flight capability between ESM and ASM adult females across most of their respective ranges results in strongly contrasting natural adult dispersal rates of the two forms, from meters (ESM) [31] to potentially kilometers per generation (ASM) [22, 32, 33]. Identification of candidate genes mediating spongy moth flight would help forge a vital management tool that is suitable for rapid flight–profiling of spongy moths intercepted outside of their natural ranges. The tool would enhance resource–limited IAS management efforts by helping to gauge the risk of spread posed by moths that potentially exhibit a wide range of flight capabilities [34–36]. Moreover, it would permit this assessment independent of the geographic origin, taxonomic status (i.e., ESM, ASM, or their hybrids), or developmental stage of intercepted specimens (adults, larvae, or egg masses) due to its ability to directly measure the invasive trait of concern.

Flight is a highly complex trait, integrating aspects of body form, metabolic output, and sensory processing [37–40]. As a result of the greater dispersal capacity and resource access it enables, flight can also strongly interact with life history characteristics such as foraging, habitat choice, dispersal, mating, and oviposition [41–43]. Evolution of flight capability may therefore involve a wide array of features. In the focal taxa considered here, this may also include daily fluctuations in female flight motivation and, in ESM, sexually dimorphic flight expression [26, 34, 44]. Previous research on spongy moth flight genetics has assessed flight inheritance [23], flight–relevant functional differentiation among ASM forms [45], and subsets of functionally compelling genes that may influence ESM and ASM flight divergence [46, 47]. However, comprehensive genome–wide surveys of multiple flight–relevant functional domains are needed to assess the breadth of traits affected by flight [48]. As a result of the diverse traits that may shape spongy moth flight, we anticipated multiple genetic flight associations to emerge from comparisons of ESM and ASM. We used several genetic approaches and a novel, explicitly flight–targeted candidate gene ranking approach to address this complexity.

We employed three genetic approaches (for shorthand, “flight analyses”) that provide complementary insights into the genetics of flight differentiation in spongy moths. First, we performed genome–wide association studies (GWAS) of adult female flight capability and forewing length across multiple populations of ESM and ASM, with the aim to survey flight–associated genotypic variants across the global range of the two subspecies. Female flight capability represents our specific trait of interest and was measured directly using ecologically relevant behavioral assays; forewing length is a morphological correlate of spongy moth flight capability [49] that we incorporated as an additional and more precisely measurable flight proxy. We also conducted an analysis of inbred female moths of contrasting flight capability deriving from repeated generations of full–sib mating from an ESM × ASM parental cross. This latter method allowed us to compare flight capability with genotypic variation at the resolution of whole–genome sequences, across individuals with relatively reduced genome–wide heterozygosity. Finally, an analysis of differential ESM and ASM gene expression across pupal development provided a means to survey the transcriptome for gene activity that distinguishes the two subspecies across a key developmental period for flight morphology.

We assessed genetic associations and gene expression differences between ESM and ASM in terms of a broad set of manually defined flight–relevant functional categories. This illuminated genome–wide functional themes that broaden our understanding of potential sources of selection shaping or influenced by spongy moth flight capability, and conversely strengthened the candidacy of individual putative flight–relevant genes. We also corroborated our findings based on flight–related research activity across an extensive literature search, and on more direct evidence for flight–relevance in a sample of the insect flight genetics literature. Our results highlight multiple functional categories associated with flight divergence between ESM and ASM. They also expose a suite of candidate genes identical or functionally parallel to those previously reported from other taxa with flight–relevant functions, including several with demonstrated selection effects in relation to flight evolution in other insects [50]. Collectively, our results suggest that flight differentiation in spongy moths has involved a broad network of traits.

## Results

### Genome–wide association study

The sequencing effort returned 521.1 million reads (range per individual: 0.3–11.0 million) across 297 individuals, representing 79 ESM (40 UC, 39 KG) and 218 ASM (60 CJ, 49 CR, 30 CN, 30 RS, 30 RM, 19 JN) (for sample origins, see Table S1). The Fast–GBS pipeline identified 94916 variants, of which 8919 variants across 2507 contigs and 294 individuals were retained following quality filtering. See Additional file 1 for a detailed summary of SNP variants (Fig. S1), GWAS model setup (Fig. S2), and output (Fig. S3).

**Fig. 2:**
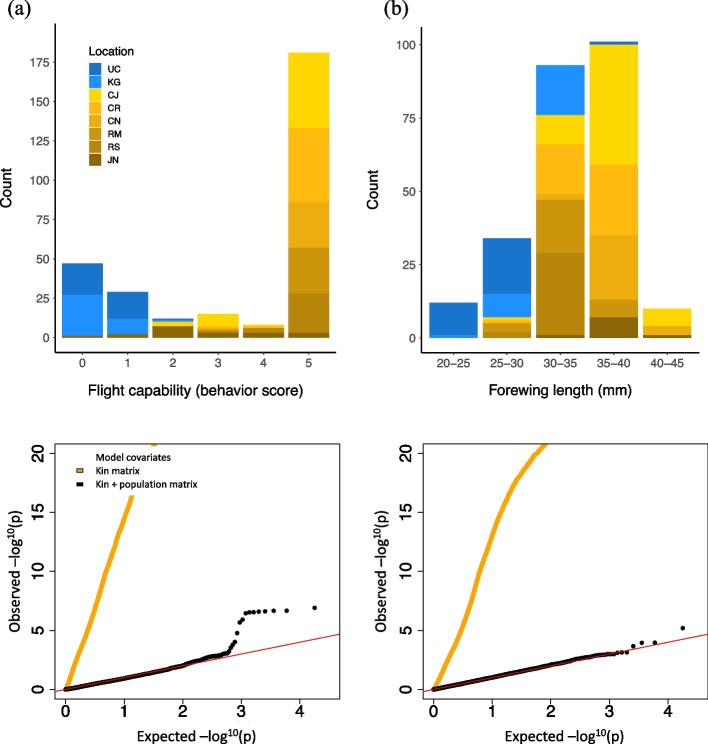
Phenotypic distributions and quantile–quantile plots of two GWAS models. **a** Flight capability (**b**) Forewing length. Strain locations are shown with two–letter codes. Blue and orange color classes follow Fig. 1, while arbitrary shades within each class distinguish individual strain locations

Flight data (Table S2) were available for 292 individuals, representing 75 ESM (38 UC, 37 KG) and 217 ASM (60 CJ, 49 CR, 30 CN, 29 RS, 30 RM, 19 JN). Flight capability scores were predominantly “0” or “5” across all individuals (Fig. 2a). We therefore binned all scores into “no flight” (flight codes 0–2) or “flight” (flight codes 3–5) and modeled the data using a binomial error link. We selected a four–PC model for downstream analysis, which represented the highest number of PCs explored among flight capability models that resolved statistically. Our results indicate that the four PCs captured a large proportion of genome-wide structure across the study system (0.36; Fig. S2a) and a quantile–quantile plot confirmed that the four–PC model successfully controlled for most or all kinship and kin-corrected geographic structure while exposing a subset of loci potentially relevant to flight capability (Fig. 2a). Specifically, the flight capability GWAS model detected 393 outlier SNPs across 303 contigs at *p* < 0.05 and one SNP at a *p*–value adjusted for multiple comparisons (p_adj_ < 0.05).

Forewing lengths (Table S2) were available for 250 individuals, representing 57 ESM (31 UC, 26 KG) and 193 ASM (58 CJ, 42 CR, 27 CN, 30 RS, 27 RM, 9 JN). Forewing lengths were approximately normally distributed (Fig. 2b) so we modeled them using a Gaussian error link. All forewing length models we explored resolved statistically. We chose for downstream analyses output from the 10–PC model–the highest number of PCs explored, (genome-wide structure explained: 0.45; Fig. 2b), although results for all models incorporating six or more PCs were highly correlated (Fig. S3d). The forewing length GWAS model detected 494 outlier SNPs across 370 contigs at *p* < 0.05 and none at p_adj_ < 0.05.

### Inbred lines

The whole–genome sequencing effort yielded high quality reads averaging 39$\times$ coverage across the ESM female parent, ASM male parent, four progeny showing strong flight capability, and four progeny showing little or no flight capability. We detected 1.38 million SNPs with high coverage and low missing rate across 14917 contigs, representing 85% of the ASM genome assembly. We identified 250 SNPs across 102 contigs that featured homozygous genotypes in all ASM parent and female progeny showing strong flight capability (average score = 5), while exhibiting opposite homozygous genotypes or only occasional heterozygotes in the ESM parent and non–flying female progeny (average score=1.25). This resulted in an average allele frequency difference between flying and non–flying groups of 0.57 (SD = 0.08).

### Gene expression during pupal development

Thirty samples were sequenced for expression analysis, comprising three replicate female pupae of each strain (ESM, ASM) sampled at one, three, five, eight and 11 days post pupation. After quality trimming, there were 6.6–20.4 million reads per sample. Of these reads, 29–64% mapped to the ASM genome (Table S3). Contamination of RNA with the L. d. iflavirus 1 (LdIV1, NCBI accession KJ629170) [51] is common in spongy moths [52] and accounted for 5–23% of unmapped reads across individuals in the present study. This content was filtered prior to further analyses.

**Fig. 3:**
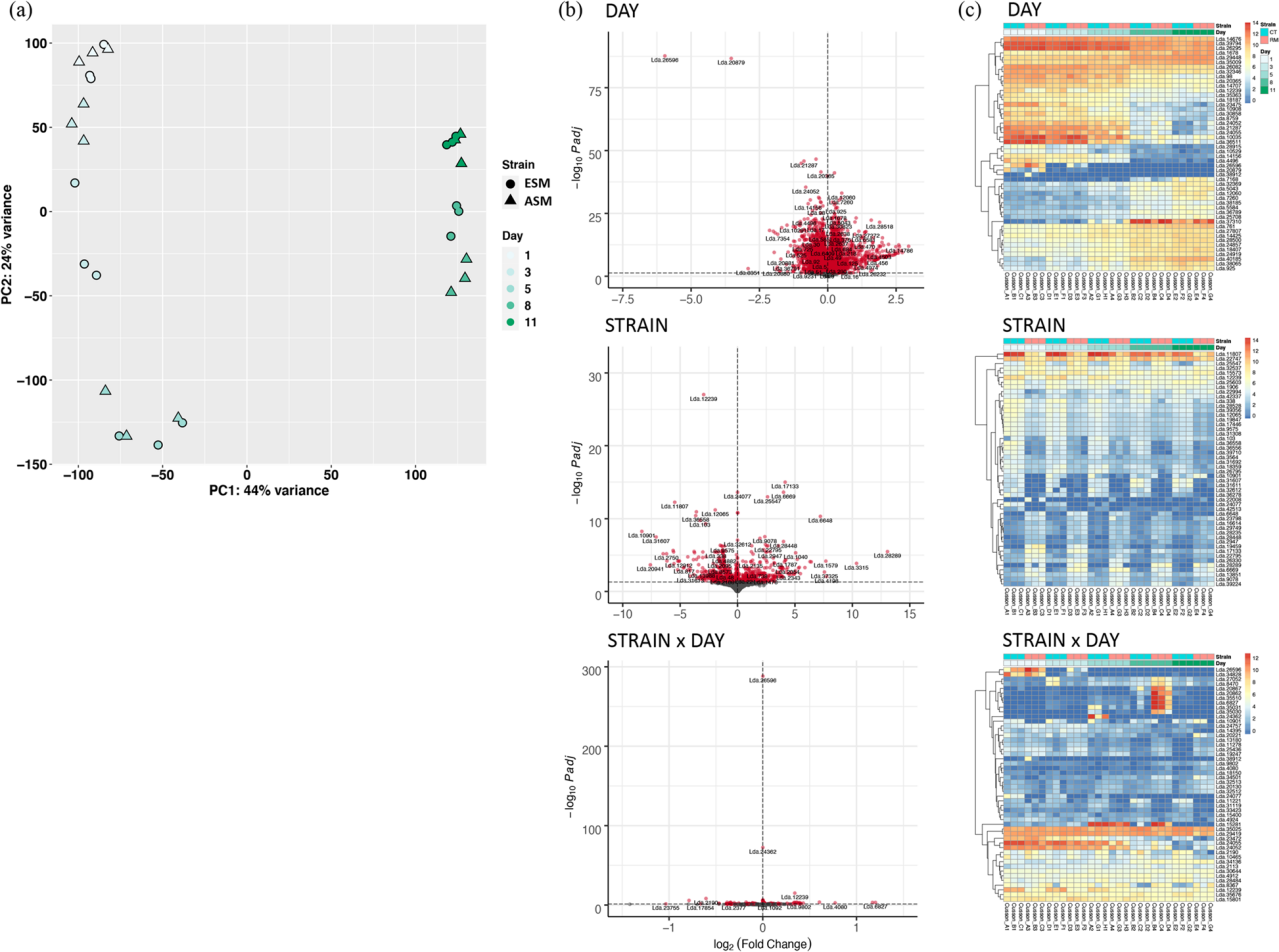
Differential gene expression between ASM and ESM pupae. **a** PCA plot based on the top 5000 most abundant transcripts (ASM: circles; ESM: diamonds). **b** Volcano plots representing differential transcript levels. Each dot represents a gene (red: significant [p_adj_ ≤ 0.05] difference in transcript levels; grey: non–significant difference). **c** Heatmaps of gene expression patterns for 50 most significant genes across all samples. Transcripts were clustered based on the Euclidean distance of their variance–stabilized counts

Of 19,654 expressed genes detected, many showed significantly different transcript levels across pupal development: 3995 were up–regulated, and 4706 were down–regulated by day (p_adj_ < 0.05). Changes between days 5 and 8 were most apparent (Fig. 3a,c). We found 357 transcripts more abundant in ASM than ESM, and 492 less abundant (Fig. 3b,c). There was a significant strain × day interaction in transcript levels for 170 genes (Fig. 3b,c, Table S4).

We used qPCR to confirm differential expression between the two strains of three potentially flight–relevant genes that we detected via the NCBI invertebrate RefSeq protein annotations (below): one *takeout* (Lda.26596) [53] and two *Osiris* genes (osiris 18 [Lda.35031] and osiris 20 [Lda.35510]) [54–57]. The *Osiris* genes were over 1000 times more abundant on day 8 in ASM than in ESM (Fig. S4). The *takeout* gene showed higher transcript levels in ASM on the first two sample points after pupation, particularly on day 3 when transcripts in that strain were more than 500 times more abundant than in ESM (Fig. S4). We examined approximately 2000 bp upstream of these genes for haplotypic differences between ESM and the *L. d. asiatica* and *L. d. japonica* genome builds of Hebert et al. [45]. We found several distinct SNPs in ESM compared to both genomes, but none within the regulatory regions of the promoters (Fig. S5).

### Gene candidacy

#### Candidate gene definition and overlap across analyses

To undertake flight–targeted functional analyses, we considered all gene expression candidates above (defined at p_adj_ < 0.05) but relaxed our candidate gene definition for the other flight analyses to include: (1) all annotated genes in strong LD (r ≥ 0.90) with GWAS loci exhibiting *p* < 0.05, and; (2) all annotated genes in strong LD (r ≥ 0.90) with inbred line loci showing strong allele segregation between flying and non–flying inbred individuals. We employed these criteria simply for the purpose of supplying a sample of top–ranked genes from each flight analysis to explore functional patterns, and we do not imply that any of the three gene sets are either confirmed flight genes or an exhaustive list of top candidates.

**Fig. 4:**
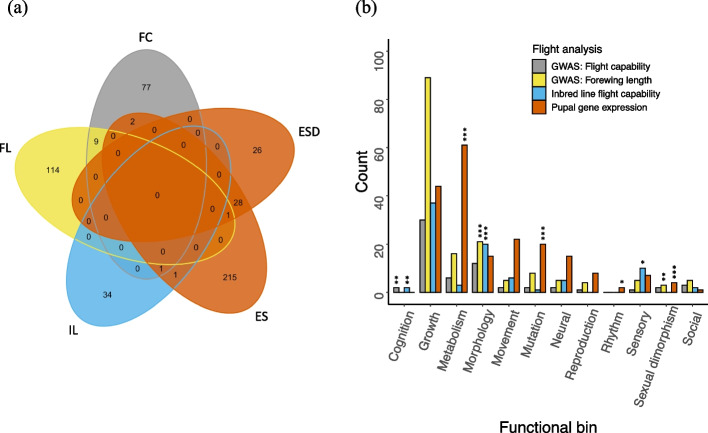
Gene overlap and functional category representation across flight analyses. **a** Numbers reflect all top–ranked genes with GO Biological Process assignments. Letters reflect type of flight analysis (FC: flight capability GWAS; FL: forewing length GWAS; IL: inbred line flight capability; ES, ESD: pupal gene expression strain, strain × day model terms, respectively). **b** Functional categories show representation by significantly enriched GO terms (*p* < 0.05). Asterisks reflect significant over–representation within each flight analysis compared to background representation in the reference annotation (binomial test, adjusted for multiple comparisons: * p_adj_ < 0.05, **, p_adj_ < 0.01, *** p_adj_ < 0.001)

We based functional analyses on a reference gene annotation created using the UniProtKB/Swiss–Prot database and comprising 24,019 gene models (69% of those queried). We focused on GO “Biological Processes” annotations for all functional analyses. This furnished 435 annotated genes across 508 outliers in total, representing 274 (26.9%) gene expression outlier gene models, 124 (25.1%) forewing length GWAS outlier SNPs, 89 (22.6%) flight capability GWAS outlier SNPs, and 36 (14.4%) inbred line outlier SNPs (Fig. S6). Candidate gene sets showed limited overlap across pairs of flight analyses, and none were shared among all analyses (Fig. 4a).

#### Functional themes across candidate genes

Enrichment analyses revealed significant (*p* < 0.05) GO term representation of annotated candidates from each of the three flight genomics analyses. Manual inspection of prominent GO term definitions allowed us to classify many terms into 12 functional categories potentially relevant to the biology of flight in spongy moths: cognition, growth, metabolism, morphology, movement, mutational mechanisms, neural processes, biological rhythms, reproductive mechanisms or behavior, sensory traits or perception, evidence of sexual dimorphism or conflict (including molecular mechanisms facilitating those processes), and social behavior (Table S5). Enriched terms fell into several of these functional categories within each flight analysis (Fig. 4b). Across analyses, GO term tallies were highest in the functional categories growth, metabolism, and morphology. All functional categories were represented in the reference annotation by more than 10 GO terms (maximum: 1412) except the social category (7 terms). We observed 7 significantly over–represented categories across flight analyses at p_adj_ < 0.05 when compared to proportional category representation in the reference annotation, with three of those being shared across analyses (Fig. 4, Table S6).

The Markov Cluster (MCL) Algorithm returned numerous significant GO term functional clusters. Those including keywords from our flight–relevant categories and receiving relatively strongest functional (10 or more GO terms) or genetic support (at least 5 genes) are summarized in Table 1 (for a summary of all clusters see Table S7). Collectively, the flight–relevant MCL clusters featured all of our manually defined flight–relevant functional categories above (Table S6), as well as most of the GO terms represented in those categories (Fig. S7). Many of the most supported clusters featuring flight–relevant GO terms reflected support for our manually defined functional categories (GWAS forewing length: 2 clusters; inbred lines: 2 clusters; gene expression: 5 clusters). However, in general the clusters frequently either revealed sub–structuring of genes (i.e., multiple clusters) within functional categories or conversely bundled various categories together, in particular with aspects of growth (Table 1). Among smaller clusters (Table S7), these functional bundles included one that implicates morphology, movement, and social functions in the context of wing–mediated mating interactions (flight performance GWAS), and another that implicates movement and social functions in the context of mating (inbred lines). Finally, multiple clusters contained no GO terms detected by our flight–relevant keywords, including 13 clusters represented by at least 5 genes (Table 1). These reflected transcriptional regulation or cellular response to stress (4 clusters); cell death or phase transition (2); protein acetylation or other modification (4), and; intra–cellular signaling (3).

**Table 1:** MCL clusters of enriched GO terms that showed greatest functional or genetic support^a^

Cluster descriptor^b^	Flight category assignments^c^	Unique GO terms	Unique genes^d^
Flight capability GWAS			
Brain, head, central, nervous	Growth (3), Neural (1)	3	7 (6–7)
Transcription, templated, nucleic, rna	Growth (1)	4	5 (3–5)
Forewing length GWAS			
Blood, vessel, vasculature, labyrinthine	Growth (4), Reproduction (1)	4	5 (1–5)
Bulb, interneuron, olfactory, tangential	Growth (2), Neural (3), Sensory (4), Social (4)	6	1 (1)
Juvenile, hormone, fate, regulation	Growth (13), Metabolism (6)	25	59 (1–59)
Pole, duplication, septin, body	Growth (2), Morphology (3), Sexual dimorphism (3)	15	31 (1–31)
Stimulus response cellular	n/a	2	36 (28–36)
Vascular, smooth, differentiation, muscle	Growth (9), Morphology (8)	9	2 (1–2)
Inbred lines flight capability			
Action, potential, cardiac, muscle	Morphology (10)	17	1 (1)
Eye, death, programmed, retinal	Growth (11), Sensory (6)	15	7 (1–7)
Modification, phosphorylation, phosphorus, phosphate	Metabolism (2)	7	9 (6–9)
Pupal gene expression			
Cytotoxicity, immune, mediated, adaptive	Growth (4), Mutation (2)	27	21 (4–21)
Diameter, vasoconstriction, blood, size	Morphology (1)	8	11 (4–11)
Endopeptidase, cysteine, type, activity	Movement (4)	4	5 (4–5)
Erythrocyte differentiation myeloid hemopoiesis	Growth (4)	5	5 (4–5)
Extrinsic absence ligand apoptotic	n/a	5	5 (4–5)
G1 transition phase cycle	n/a	4	5 (4–5)
Growth, cell, negative, regulation	Growth (2)	2	7 (5–7)
Import, chaperone, autophagy, process	Growth (3), Metabolism (37), Mutation (3), Reproduction (4)	73	252 (2–223)
Internal acetylation lysine peptidyl	n/a	6	8 (4–8)
Mannose, hexose, monosaccharide, metabolic	Metabolism (3)	3	5 (3–5)
Mitochondrion targeting intracellular protein	n/a	9	5 (4–5)
Modification protein process cellular	n/a	2	38 (38)
Nutrient levels starvation extracellular	n/a	3	9 (6–9)
Perception, sensory, smell, chemical	Sensory (3)	3	10 (5–10)
Permeability lysosomal lytic membrane	n/a	8	5 (4–5)
Polymerization, microtubule, supramolecular, inclusion	Growth (6), Morphology (4)	28	23 (2–18)
Promoter polymerase II transcription	n/a	3	5 (5)
Proteasomal, ubiquitin, catabolic, proteolysis	Metabolism (11)	12	6 (4–6)
Refolding folding protein regulation	n/a	6	6 (2–6)
Removal conjugation ubiquitination modification	n/a	3	10 (4–10)
Rho ras transduction signal	n/a	3	6 (2–6)
Skeletal, muscle, organ, tissue	Growth (3), Morphology (3)	3	6 (4–6)
Slow, axo, axonal, dendritic	Morphology (1), Neural (2)	4	5 (4–5)
Specialization, maintenance, postsynaptic, structure	Neural (4)	6	5 (4–5)
Topoisomerase, hydrolyzing, isomerase, atp	Movement (6)	6	6 (3–6)
Topologically incorrect unfolded response	n/a	4	5 (4–5)
Transporter, calcium, transmembrane, coupled	Movement (6)	10	5 (3–4)
Viral, genome, modulation, host	Mutation (4)	14	5 (4–5)

^a^Both cluster membership (clusterMaker) and GO enrichment (BiNGO) were assigned at *p* < 0.05. Shown are clusters receiving 10 or more category hits, and/or support from 5 or more genes (for a list of all clusters see Table S7). Results are partitioned by flight analysis

^b^Cluster descriptors reflect the four most common words from across the definitions of GO terms comprising each cluster

^c^Numbers in parentheses reflect the number of GO term hits assigned to flight–relevant functional categories (“n/a”, no GO terms assigned)

^d^Numbers in parentheses reflect the range of genes representing individual GO terms within a given cluster

We conducted an additional search for matches between the pupal gene expression data and NCBI invertebrate RefSeq proteins. This effort revealed several gene models for a cluster of transcripts exhibiting significantly increased abundance in ASM compared to ESM pupae on day 8 (Fig. 3c). The genes included putative annotations of zonadhesin (Lda.8470 and Lda.27052), osiris 18 (Lda.6827 and Lda.35031) and osiris 20 (Lda.35510; as well as Lda.35030, another *Osiris* gene), peroxisomal catalase (Lda.20862 and Lda.20867), a circadian clock–controlled protein takeout (Lda.26596), and a putative tweedle cuticular protein (Lda.15281). Genes uniquely showing strain effects clustered with transcripts for putative tweedle cuticular proteins (Lda.20030 and Lda.12768), cuticle protein 19.8 (Lda.23652), and alpha–tocopherol transfer protein (Lda.4455) (Fig. 3c). Several of the genes above were also detected earlier using the UniProtKB/Swiss–Prot annotation (Lda.20862, Lda.20867, Lda.4455, and Lda.26596 [shared annotation with Lda.458]).

#### Targeted literature searches for individual gene candidacy

We searched for additional support of individual candidate genes serving flight–related functions via customized, flight–targeted literature searches within each functional category described above. This approach bypassed the stringent standards for functional assignment in gene databases. Instead, our search focused on surveying the scientific literature for bulk evidence of flight–relevant research activity focused on putative candidate genes. In a taxonomically restricted effort we first examined a selection of insect–specific studies that have addressed the genetics of flight or migratory variation. The 32 articles examined featured a range of species and a variety of candidate gene or genome–wide approaches (Table S8). The survey uncovered matches with 48 (11.0%) of our flight candidate genes from across all flight analyses (Table 2; for gene descriptions see Table S9) [18, 47, 50, 58–74], reflecting a lower bound on the expected proportion of matches of our candidates with those across the entire insect–specific flight literature. Of the gene matches observed, 12 were exact, four of which were previously reported by Mitterboeck et al*.* [50]*,* (Table 2, column “Literature Support/Insect–targeted/Match”) as showing molecular signatures of selection during evolutionary flight divergence among sister taxa. The remaining genes were either functionally comparable to flight–implicated genes from a variety of insects based on UniProtKB/Swiss–Prot and NCBI gene names or functional definitions (20 genes, seven of which showed flight–related selection in insects) [50], apparently interact with flight–implicated genes from the other insects reviewed (14 genes), or both (two genes, both of which showed flight–related selection in insects; Table S10) [50]. Among the associations above, three reflected exact matches with flight candidates from the comparative genomic analysis of ASM and ESM by Zhang et al*.* [47]*,* (Table 2), and two others showed close apparent functional overlap (Discussion). Eleven additional genes matching those detected by Zhang et al. [47] do not show obvious direct flight relevance (Table S9).

**Table 2:** Candidate genes showing combined statistical significance and flight relevance from previous insect flight genetics research

Gene^a^	Statistical support (p/p_adj_)^b^					Functional support^c^								Literature support														
						GO terms enriched				Functional category membership (no. enriched GO terms)				Insect-targeted			Taxonomically general											
	FC	FL	ES	ESD	IL	FC	FL	ES/ESD	IL	FC	FL	ES/ESD	IL	Match^d^	Function reported	Source	Cog	Gro	Met	Mor	Mov	Mut	Neu	Rep	Rhy	SD	Sen	Soc
ACOX3	n/a	0.016/0.874	n/a	n/a	n	0	0	0	0	n/a	n/a	n/a	n/a	C	Met	58	0	2	8	1	0	3	1	1	0	1	0	1
ACVR1	n/a	n/a	n/a	0.000/0.004	n	0	0	10	0	n/a	n/a	Met (7)	n/a	E	Mor	59, 60	3	60	24	42	6	67	10	6	0	14	7	4
ARP2	n/a	n/a	0.001/0.022	n/a	y	0	0	14	14	n/a	n/a	n/a	Mor (1)	**C, I**	Mor, Met	48, 50, 61	2	68	86	50	54	95	64	2	0	9	15	72
BX42	n/a	n/a	0.000/0.000	0.017/0.316	n	0	0	13	0	n/a	n/a	Met (12)		I	Gro, Met	61, 62	0	1	0	2	0	1	0	0	0	0	1	0
CADF	n/a	n/a	n/a	0.044/0.448	y	0	0	0	6	n/a	n/a	n/a	Mor (1)	I	Mor	61	0	2	0	1	0	2	1	0	0	1	0	0
CBL	n/a	n/a	n/a	n/a	y	0	0	0	0	n/a	n/a	n/a	n/a	I	Mor	61, 63–65	71	468	317	263	58	447	115	25	2	97	56	73
CDK2	n/a	n/a	0.001/0.044	n/a	n	3	4	18	10	n/a	n/a	Met (13)	n/a	**C**	Met	50	6	832	409	422	62	643	120	37	1	165	30	92
DLL	n/a	0.033/0.882	n/a	n/a	n	0	13	0	0	n/a	n/a	n/a	n/a	E	Mor	61, 63	14	114	39	93	11	50	35	10	1	15	27	16
DPP6	n/a	n/a	0.001/0.040	0.022/0.344	n	0	0	5	0	n/a	n/a	Met (5)	n/a	C	Mor	61	0	1	0	0	0	1	0	0	0	0	0	0
E75	n/a	0.007/0.792	n/a	n/a	n	0	13	0	0	n/a	n/a	n/a	n/a	I	Gro	66	1	11	8	2	1	3	4	3	0	3	1	1
FA2H	0.002/0.561	n/a	n/a	n/a	n	1	0	0	0	n/a	n/a	n/a	n/a	C	Met	58	5	5	6	3	6	12	13	0	0	1	6	1
FGFR1	n/a	0.036/0.882	0.041/0.294	n/a	n	0	57	0	0	n/a	Mor (3)	n/a	n/a	C	Mor, Mov	47	2	122	45	39	11	70	34	1	1	28	7	18
FYV1	n/a	n/a	0.000/0.000	n/a	n	0	0	5	0	n/a	n/a	Met (4)	n/a	C	Met	58	0	0	1	0	0	1	0	0	0	0	0	0
G6PI	n/a	0.003/0.752	n/a	n/a	n	0	1	0	0	n/a	n/a	n/a	n/a	C	Mor	67	0	2	1	0	0	3	1	0	0	0	0	1
GEK	n/a	n/a	0.002/0.049	n/a	n	0	0	10	0	n/a	n/a	Met (7)	n/a	**E**	Met	50	6	25	11	22	13	12	35	4	3	4	8	8
GLU2B	n/a	n/a	0.001/0.037	n/a	n	0	0	9	0	n/a	n/a	Met (8)	n/a	**C**	Neu	50	0	0	0	0	1	0	1	0	0	1	0	0
GRIK2	n/a	0.012/0.837	n/a	n/a	n	0	0	0	0	n/a	n/a	n/a	n/a	E	Cog, Mov	47	4	11	4	6	3	18	16	1	0	5	2	5
HIPK2	0.032/1.000	n/a	n/a	n/a	n	0	0	0	0	n/a	n/a	n/a	n/a	**C, I**	Mor, Met	50, 63, 68, 69	0	11	11	4	1	5	0	0	0	4	0	1
HR38	n/a	0.033/0.882	n/a	n/a	n	0	13	0	0	n/a	n/a	n/a	n/a	E	Rep	70, 71	1	4	7	3	1	3	3	2	0	2	1	0
IDH3B	0.046/1.000	n/a	n/a	n/a	n	1	0	0	0	n/a	n/a	n/a	n/a	**C**	Met	61, 50	0	4	4	2	1	5	0	2	0	1	1	4
IPP	n/a	n/a	n/a	n/a	y	0	0	0	0	n/a	n/a	n/a	n/a	I	Mor	61	807	1007	993	1238	411	632	1565	177	70	525	301	270
ITPR	n/a	n/a	0.001/0.029	0.000/0.045	n	0	0	1	0	n/a	n/a	n/a	n/a	C	Met	58	0	6	5	1	1	5	9	0	0	2	1	4
JMJD7	n/a	n/a	0.001/0.033	0.008/0.225	n	0	0	10	0	n/a	n/a	Met (7)	n/a	C	Met	58	0	1	0	0	0	1	0	0	0	1	0	0
KPCA	n/a	n/a	n/a	n/a	y	0	0	0	0	n/a	n/a	n/a	n/a	**C**	Met	50	1	2	1	0	0	0	0	0	0	0	0	0
M3K4	n/a	n/a	0.000/0.008	0.000/0.009	n	0	0	10	0	n/a	n/a	Met (7)	n/a	E	Met	47	0	0	0	0	0	0	0	0	0	0	0	0
NPY2R	0.019/1.000	n/a	n/a	n/a	n	1	0	0	0	n/a	n/a	n/a	n/a	C	Neu	58	0	0	0	0	0	0	0	0	0	0	0	0
ORC3	0.018/1.000	n/a	n/a	n/a	n	0	0	0	0	n/a	n/a	n/a	n/a	**C**	Cog	50	2	6	9	2	0	15	4	2	0	2	2	2
OSP	n/a	0.047/0.883	n/a	n/a	n	0	23	0	0	n/a	Mor (2)	n/a	n/a	I	Mor	61	1069	7565	6851	7091	1543	5686	5139	1120	125	2694	1366	1498
PAF1	0.004/0.681	n/a	n/a	n/a	n	4	0	0	0	n/a	n/a	n/a	n/a	I	Mor	63	0	6	2	3	1	6	2	1	0	1	0	2
PI4KA	n/a	0.042/0.882	n/a	0.026/0.365	n	0	6	0	0	n/a	n/a	n/a	n/a	C	Met	58	0	1	1	0	0	3	0	1	0	1	0	0
PICAL	0.004/0.648	n/a	n/a	n/a	n	1	0	0	0	n/a	n/a	n/a	n/a	C	Met	58	228	1639	1143	1704	472	1199	961	257	40	651	472	371
PIGP	n/a	n/a	n/a	0.000/0.029	n	0	0	16	0	n/a	n/a	Met (14)	n/a	C	Met	58	0	4	2	1	0	2	0	0	0	0	0	1
PSMD8	n/a	n/a	0.048/0.317	0.000/0.015	n	0	0	5	0	n/a	n/a	Met (5)	n/a	**C**	Met	50	1	3	0	0	0	0	0	0	0	1	0	1
RAC1	n/a	0.019/0.874	n/a	n/a	n	0	6	0	0	n/a	n/a	n/a	n/a	I	Mor	61, 72	46	800	655	514	416	839	400	30	4	173	68	537
RFC4	0.015/0.973	n/a	0.002/0.045	n/a	n	0	0	6	0	n/a	n/a	Met (5)	n/a	**E**	Met	50	0	2	1	1	0	4	0	0	0	2	0	0
RIOK2	n/a	n/a	0.000/0.009	0.046/0.456	n	0	0	10	0	n/a	n/a	Met (7)	n/a	**E**	Met	50	0	0	0	0	0	0	0	0	0	0	0	0
RL4	n/a	n/a	0.002/0.046	n/a	n	0	0	9	0	n/a	n/a	Met (8)	n/a	**C**	Reg	50	1	14	12	3	1	13	3	2	0	3	0	1
SMAD3	0.043/1.000	n/a	n/a	n/a	n	3	0	0	0	n/a	n/a	n/a	n/a	I	Mor	63, 64, 73	4	1018	452	448	69	393	75	37	2	132	24	108
SNF5	n/a	n/a	0.002/0.048	n/a	n	0	0	2	0	n/a	n/a	Met (1)	n/a	I	Mor	61	1	49	22	17	0	62	7	2	0	8	4	19
SODC	0.048/1.000	n/a	n/a	0.007/0.215	n	3	0	0	0	n/a	n/a	n/a	n/a	**E**	Met	50	1	2	3	0	1	1	1	0	0	0	0	0
SPR	n/a	n/a	0.014/0.163	n/a	y	0	0	0	0	n/a	n/a	n/a	n/a	E	SD	18	559	3228	2507	2894	907	1928	2149	634	63	959	577	866
TAKT	0.041/1.000	n/a	n/a	n/a	n	0	0	0	0	n/a	n/a	n/a	n/a	E	Rep, Rhy	74	3	10	4	13	7	6	13	2	1	1	5	4
TLD	n/a	n/a	0.001/0.023	0.016/0.307	n	0	0	1	0	n/a	n/a	n/a	n/a	I	Neu	65	41	239	31	257	41	68	66	152	1	25	79	13
TLN1	n/a	n/a	0.001/0.043	0.000/0.030	n	0	0	1	0	n/a	n/a	n/a	n/a	E	Mor	61, 47	0	2	1	1	0	1	0	0	0	1	0	2
TLN2	n/a	n/a	0.006/0.102	0.000/0.005	n	0	0	1	0	n/a	n/a	n/a	n/a	I	Mor	61	0	2	2	0	0	2	0	0	1	0	0	0
TLR4	n/a	n/a	0.000/0.008	0.001/0.062	n	0	0	1	0	n/a	n/a	n/a	n/a	C	Cog, Mov, Rhy	47	89	1188	1673	947	150	1089	400	98	6	424	116	249
TSG	n/a	0.026/0.882	n/a	n/a	n	0	8	0	0	n/a	n/a	n/a	n/a	I	Mor	61	1	73	41	33	16	48	35	5	0	11	8	16
WASF1	0.004/0.648	0.028/0.882	n/a	n/a	n	0	5	0	0	n/a	Mor (2)	n/a	n/a	I	Mor	61	0	1	1	0	1	0	1	0	0	0	0	1

^a^For gene definitions and reference genetic markers associated with each gene see Table S9

^b^Flight analyses are abbreviated as flight capability GWAS (FC), forewing length GWAS (FL), pupal gene expression Strain term (ES) and Strain × Day interaction term (ESD), and inbred lines flight capability (IL). Statistical support reflects the genetic marker showing strongest support for a given gene. Only values significant at *p* < 0.05 or greater are reported. IL genotypic flight associations were judged qualitatively as yes/no

^c^Functional categories are abbreviated as cognition (Cog), growth (Gro), metabolism (Met), morphology (Mor), movement (Mov), mutational mechanisms (Mut), neural (Neu), reproduction (Rep), circadian rhythm (Rhy), regulatory (Reg; excluded from taxonomically general literature search), sexual dimorphism (SD), sensory (Sen), and social (Soc). Significance thresholds are as follows: *p* < 0.05 (GO enrichment), p_adj_ < 0.05 (functional category)

^d^Status of currently identified candidate genes in relation to those previously reported from insect flight research is depicted as exact (E), functionally comparable (C), or functionally interacting (I) based on UniProtKB/Swiss–Prot gene names or functional definitions. Those in bold highlight candidates associated with direct molecular evidence for flight-related evolution by Mitterboeck et al. [50]

We also identified 49 unique genes from differentially expressed transcripts between ESM and ASM spongy moth pupae that are orthologous to those involved in wing development signaling pathways in the moth *Ostrinia furnacalis* and the locust *Locusta migratoria manilensis* (Table S11) [75]. Of these, 27 differed significantly by day in the spongy moth pupae, none by strain, and one showed a significant strain × day interaction (Lda.35676, a putative bone morphogenetic protein type I receptor, *Saxophone*, in the decapentaplegic signaling pathway) [76]. We also identified 43 unique spongy moth genes orthologous to proteins involved in a wing–size phenotype in *Drosophila* (Table S12) [77]. Twenty–five of these differed significantly by day in the spongy moth pupae, but none by strain or strain × day.

In a taxonomically unrestricted literature search covering the years 1970–2020 we obtained 166556 article hits across all functional categories (Fig. S8). The hits addressed 268 (49.9%) of all annotated candidate genes and 261 of these (48.6%) featured references relevant to multiple functional categories across articles, while 61 (11.3%) featured references relevant to all 12 categories (Table S9). Total publication volume per gene across all functional categories in the unrestricted literature search varied widely (range: 1–65613 hits; median: 28 hits). The 48 of these candidates distinguished above during the insect–specific literature search (Table 2) also reflected this general pattern. Total research activity per gene for the 48 insect–specific candidates ranged from 2–41747 hits across all functional categories (median: 46 hits; Table S9).

### Flight capability prediction success of top candidate genes

Twelve SNP loci exhibiting outlier status in the flight capability GWAS also received independent flight–associated support from our sample of recent insect flight literature (i.e., Flight capability GWAS statistical outliers in Table 2). These loci collectively showed an average success of 0.882 (± 0.008 SE) in assigning individuals to their correct binary flight category across replicated test runs, significantly more than expected by chance (binomial tests for each of 10 profiling replicates: *p* < 0.001).

## Discussion

The evolutionary gain or loss of flight represents a stark turning point in insect life history, due to the proximate demands of flight itself and the unique ecological opportunities that it brings within reach [41, 42, 78]. As a result of the potentially wide array of traits affecting flight evolution, molecular signatures of this process are likely to reflect changes in a variety of inter–related functions. Documenting this signal presents a way to strengthen the candidacy of individual genes through knowledge of their integration within functional patterns across the genome, and to consider what the functional patterns themselves reveal about the forces mediating flight evolution. We searched for genes underlying spongy moth flight capability from this mechanistic perspective by using several genetic approaches to assess multiple flight–relevant functional themes distinguishing *L. d. dispar* (European spongy moth, ESM) from *L. d. asiatica* and *L. d. japonica* (representative Asian spongy moths, ASM).

### Flight–targeted gene candidacy

Our approach for identifying flight candidate genes centered on assessing membership of statistically top–ranked genes from each flight analysis to flight–relevant functional categories [79–81], then comparing category membership of these gene sets to background patterns across all annotated genes. Our premise for this approach is that significantly over–represented functional themes reflect collective evidence for causal functional drivers or outcomes, whereas false positive associations are unlikely to produce cohesive themes. Our aims in employing manually defined flight–relevant functional categories were twofold: (1) to explore genome–wide signal of functional features relevant to the biological context of evolutionary divergence in spongy moth flight capability, and; (2) to employ that functional signal in our appraisal of the flight candidacy of individual genes. The first aim permitted a specifically flight–oriented functional interpretation of our results, although we also employed conventional (i.e., functionally general) enrichment tests to corroborate the breadth of GO term representation by our method and to broaden our functional insights into spongy moth flight evolution. The second aim leveraged the cumulative flight–relevant functional signal that may be present across genome–wide markers, even where that signal is diffuse as is commonly the case for polygenic traits [82, 83]. This added a signal–driven functional criterion to help expose flight–relevant genes that may reflect only modest statistical support owing to their small individual effects or, in the case of GWAS, to practical limits on experimental replication [84, 85]. Below, we discuss potential research applications of candidates uncovered by our functionally flight–targeted approach, including the subset of those that met a relatively stringent statistical candidacy criterion (p_adj_ < 0.05).

### Functional integration of flight

The results expose differentiation between ASM and ESM along multiple flight–relevant functional axes. Several of the significantly overrepresented functional categories we observed (Fig. 4) reflect proximate constraints that are known to broadly mediate flight evolution across other taxa, such as morphological and metabolic demands of flight (e.g., [86–89]), and cognitive and sensory requirements of navigation and flight performance [90]. Other categories may reflect relatively narrower aspects of flight evolution in ESM, including a potential modification of behavioral rhythms underlying daily fluctuations in ASM female flight activity [34, 44], or molecular mechanisms facilitating the evolution of sexually dimorphic flight capability [91–93]. Additional bundling of functional categories by the MCL algorithm, particularly with aspects of growth (e.g., tissue morphogenesis, differentiation, development; Table 1), suggests further functional integration of these broad themes. Confirmation of these patterns awaits a more complete annotation of the reference genome we used (Fig. S6) [94]. Nonetheless, assuming the present annotation approximates an unbiased sample of functional variation across the spongy moth genome, we expect additional data will uphold our general finding of diverse functional differentiation between spongy moth lineages. Overall, this pattern indicates that divergence in flight capability between European and Asian spongy moth lineages has coincided with evolutionary differentiation in multiple aspects of flight development, execution, and surrounding life history.

The functional patterns above suggest that multiple traits may have contributed to flight loss in ESM females. Indeed, our analyses returned many genes previously implicated in flight specifically or locomotion in general. Considering only *L. dispar* subspecies, we observed several direct matches or functional parallels with potentially flight–relevant genes previously detected via whole–genome comparisons between *L. d. dispar* and *L. d. asiatica*. Specifically, Zhang et al*.* [47] reported divergent genes implicated in insect flight muscle contraction (multiple L–glutamate receptors, voltage–gated calcium channel proteins, and cytoskeletal protein talin 1) and wing development (a mitogen–activated protein, a Toll–like receptor, *lingerer*, *capua*, *snake*, and *easter*). In line with both themes, we observed direct matches with glutamate receptor ionotropic kainate 2 (GRIK2, Lda.4585), talin 1 (TLN1, Lda.16464), and mitogen–activated protein kinase kinase kinase 4 (M3K4, Lda.15801), as well two functionally comparable genes (Table 2): Toll–like receptor 4 (TLR4, Lda.35963), which shows evidence for effects on cognition [95], locomotion [96], and circadian rhythm [97] in mammals, and; fibroblast growth factor receptor homolog 1 (FGFR1, Lda.18769), which shows morphological effects in birds [98] and locomotory effects in mice [99, 100]. Expanding our assessment to a selection of other insect species subject to research on flight evolution exposed 43 additional candidates that either constitute exact matches, play comparable roles, or interact with previously reported flight candidate genes, based on UniProtKB/Swiss–Prot and NCBI gene names or functional definitions (Table 2). Notably, although the insect–specific literature we inspected featured flight and migration gene candidates from an array of taxa and genetic approaches (Table S8), this represents only a partial survey (e.g., [101–105]). A comprehensive assessment, including analyses at the level of shared functional pathways, will likely illuminate further taxonomic parallels in flight–relevant genes. A subset of those genes may already be represented within our bulk returns from the general literature, which suggested that at least 267 (61.4%) of our annotated flight candidates have been subject to functionally flight–relevant research activity in general (Table S9). Interestingly, while the insect–specific literature support highlighted several significantly over–represented functional categories in our study, the taxonomically general literature returns for those genes suggested that many may in fact contribute to multiple flight–relevant functional themes (Table 2).

Despite the candidate matches above, literature returns were overall highly variable in relation to the statistical or flight–relevant functional candidacy of individual genes in the present study (Table 2, Table S9). Further, we failed to detect a range of well–supported candidates from several previous insect–specific studies of flight genetics (Table S8) [77, 106–113], and overlap among those studies is also mixed. Differences in mechanistic focus, genetic coverage or annotation success, or statistical power across studies may explain many of these discrepancies, as exemplified in the several flight analyses we conducted. The gene expression analysis highlighted almost totally novel candidate genes compared to the other flight anlayses (Fig. 4a), suggesting there is little overlap between differential gene activity among flight types during pupal development and genotypic correlates of adult female flight capability or forewing length. Inbred line flight capability candidate returns were relatively limited, likely due to the small number of individuals tested and strict genotypic criteria used to define individual gene candidacy. Nonetheless, the whole-genome sequence resolution of that analysis contributed potentially valuable candidates, given that none of the SNPs it exposed were represented in the GWAS data matrices. Greatest candidate overlap between analyses was observed for the flight capability and forewing length GWAS’s, in line with the association between those phenotypic measures (Fig. 2) [49]. Here, candidate differences may stem from a combination of partially differing samples available for each analysis, different error links required for each model, and power limits or false positive detections returned as a result of the small sample size tested. It is also possible that the genetic relationship between forewing length and overall flight capability varies across the geographically widespread locations we sampled—partially obscuring a proportion of true associations when all locations are analysed together. In particular, forewing measures for the Greek ESM samples (“KG”) were on average markedly higher than those for other ESM samples showing comparably low flight capability (“UC”, Fig. 2). Overall, these considerations suggest that the diverse candidate returns from across flight analyses may offer useful insights to the breadth of genetic variation potentially shaping spongy moth flight capability, but they also emphasize the need for corroboration via comparative or functional support.

Across well–powered surveys of common mechanistic domains, unshared candidate genes apparently reflect true differences in flight genetics among taxa. For example, our analysis of spongy moth pupal development did not detect differential gene regulation at any of the proteins associated with changes in *Drosophila* wing shape in a proteomics study by Okada et al. [77], and detected only one of the many *Drosophila* wing development–related signaling pathway genes found in the transcriptomes of *Ostrinia furnacalis* and *Locusta migratoria manilensis* [75]: a putative *saxophone* (Lda.35676, vertebrate ortholog: ACVRI, Table 2) in the decapentaplegic signaling pathway that in *Drosophila* affects wing development [59] and venation [60]. Apparently, the subtle differences in wing shape associated with differential flight capability in ESM and ASM (Fig. 2) [49] do not reflect expression differences in the majority of those genes. We noted above several candidates detected in common with the findings of Zhang et al. [47] that may influence spongy moth wing morphology. Further, ecdysteroid–induced programmed cell death has been implicated in wing dimorphism across the Lymantrinnae [109, 110] and may be represented in our results by flight–associated genotypic divergence between ESM and ASM at the ecdysone receptor gene E75 (Lda.21602) [66] and the isocitrate dehydrogenase gene IDH3B (Lda.7112, Table 2) [114]. True differences in flight candidate genes among taxa may reflect either the existence of distinctive mechanisms of flight evolution or diverse molecular influences on shared gene pathways. Formal comparative tests across species at the level of gene pathways would help to resolve these alternatives.

The familiar ecological context of evolutionary loss of flight in ESM suggests that the functional breadth of genomic patterns we observed may also characterize other taxa that undergo evolutionary transitions in flight. Differentiation of ESM from the ancestor of ASM approximately 0.35–0.40 mya followed an expansion, probably from the Caucasus Mountains area, into the predominantly broadleaf forest habitat that is found throughout its present European range [115–117]. The stability and abundance of several primary tree host species within that region (e.g., *Quercus* spp.) are thought to be key factors favoring ESM female flight loss [22], given that in this setting even undirected dispersal of larvae over short distances following their spring hatch would often put them in proximity of suitable foliage for development [22, 26]. Unlike ESM, differentiation of ASM may have favored retention of female flight as a means to optimize access to preferred hosts or oviposition sites across the predominantly coniferous and more seasonal habitat of their Asian ranges [22, 26, 118]. Baranchikov and Sukachev [22] reviewed research suggesting that ASM in central and eastern Asia require reliable dispersal between alternate habitats (rocky outcrops in open landscape for larvae, forest tree trunks for adults) to acquire sufficient temperature days to complete their life cycle. This ecological pattern characterizing evolutionary transitions in flight ability is widely documented across insects, which show a positive relationship between habitat permanence and some degree of evolutionary flight loss [41, 42, 119]. Female–limited flight loss is also common among insect species that exhibit sexually dimorphic flight capability [41, 43, 120], and in ESM this feature likely reflects an adaptive partitioning of sex–specific reproductive priorities under the permissive ecological conditions of its European habitat—relaxed flight costs in females to heighten fecundity (e.g., [121–123]) but retained flight capability in males to favor mate searching [42]. Given the suite of traits affecting or shaped by flight, as well as shared ecological circumstances of transitions in this dispersal strategy across taxa, the flight–associated functional patterns we have observed in spongy moths may resemble those occurring in other species showing evolutionary flight loss (i.e., shared “dispersal syndromes”) [40, 124], despite species–specific variation in the genes underlying that phenotypic network.

### Genetic characterization of flight

Our functionally flight–targeted approach supports the use of spongy moth flight gene candidacy definitions that are flexible to different research goals or management applications. A strict shortlist of genes suited for validation research might include only candidates exhibiting strong statistical support and compelling evidence of flight–relevant functionality. From the several flight analyses we conducted, the gene expression analysis alone furnished genes surpassing a conservative statistical threshold while also showing overrepresented functional categories, enriched flight–relevant GO terms, widespread flight–relevant literature activity, or a combination of these supporting criteria (200 genes; Table S9). Table 2 highlights 21 of these genes that also received support from within our insect–specific flight research literature search, potentially reflecting taxonomically proximate signatures of spongy moth flight evolution. This shortlist may point to key developmental differences between ASM and ESM female flight capability at the pupal stage. Genotypic sources of these expression differences are unknown. Promoter region sequences of three differentially expressed genes that we confirmed via qPCR do not differ between strains, suggesting divergent regulation of these genes originates at other genomic regions.

The results also support consideration of a broader set of genes that are potentially functionally relevant to spongy moth flight, but that are currently defined only qualitatively (inbred line analysis), observed with relatively low statistical power (GWAS), or of potentially small individual effect size (any analysis). Of 435 candidate genes detected by this approach (Table S9) 27 emerged from the insect–specific flight literature search in addition to the gene expression outliers above (Table 2). These candidates uniquely reflect two of the five genes matching or comparable to those reported by Zhang et al*.* [47] based on ESM–ASM whole–genome comparisons, as well as five of 13 genes apparently associated with direct molecular signatures of evolutionary transitions in flight across insects [50]. As the diverse functional themes returned by our current analyses suggest, this more liberally defined candidate gene set may be best suited for investigations of the genetic architecture of spongy moth flight and the ecological conditions in which it evolved.

The potential for a reliable molecular assay to profile female flight capability for regulatory purposes warrants further investigation, given the implications of this trait for invasion capacity and its variation across the global range of spongy moths [34, 35]. We confirmed that markers for 12 genes detected in direct association with female flight data (flight capability GWAS statistical outliers in Table 2) showed strong flight predictive strength (88%). These are implicated in a range of flight–relevant functional roles, including several key pathways for wing or muscle development, metabolism, and circadian rhythm (Table 3), suggesting they may present a promising step toward a reliable and functionally broad gene panel for spongy moth flight profiling. However, under the global distribution of flight variation sampled in the present study, we cannot rule out a potential role for geographic artefacts (e.g., genetic drift, non–flight related selection) driving false flight associations at these genes, despite our statistical precautions against this source of error in the GWAS model (see Methods, Fig. 2). We suggest that tests on specimens collected from within a single strongly flight–variable population are needed to more rigorously distinguish putatively causal spongy moth flight genes, either substantiating or replacing the candidates above.

**Table 3:** Functions of candidate genes used in flight capability profiling analysis

Locus	Gene	Gene definition	Function	Implicated pathway or role
Lda.16452	HIPK2	Homeodomain-interacting protein kinase 2	Growth	Hedgehog (Hh), Wingless (Wnt) signaling
Lda.22240	NPY2R	Neuropeptide Y receptor type 2	Growth	JH hormone
Lda.29984	ORC3	Origin recognition complex subunit 3	Growth	DNA replication
Lda.16374	PAF1	RNA polymerase II-associated factor 1 homolog	Growth	Hedgehog (Hh) signaling
Lda.31456	PICAL	Phosphatidylinositol-binding clathrin assembly protein LAP	Growth	Decapentaplegic (Dpp) signaling
Lda.11863	RFC4	Replication factor C subunit 4	Growth	DNA replication
Lda.8391	SMAD3	Mothers against decapentaplegic homolog 3	Growth	Transforming growth factor beta (TGFB) signaling, Decapentaplegic (Dpp) signaling
Lda.31200	FA2H	Fatty acid 2-hydroxylase	Metabolism	Fatty acid metabolism
Lda.7112	IDH3B	Isocitrate dehydrogenase	Metabolism	Fatty acid metabolism
Lda.19357	SODC	Superoxide dismutase	Metabolism	Oxygen metabolism
Lda.23349	WASF1	Wiskott-Aldrich syndrome protein family member 1	Muscle function	Actin regulation
Lda.458	TAKT	Protein takeout	Behavior	Circadian rhythm

## Conclusions

This study offers a novel approach to advance our understanding of genetically complex traits. We integrated multiple complementary genetic methods that collectively capture a wide breadth of spongy moth flight–relevant signals across the genome. We then surveyed top–ranked candidate genes for flight–relevant functional themes to investigate potential evolutionary drivers or outcomes of divergent spongy moth flight evolution, and to substantiate the candidacy of individual genes. Our approach was aimed at balancing functional and statistical support for judging individual gene candidacy, by advocating for trait–targeted, functionally comprehensive surveys and application of statistical thresholds tailored to research needs. In our view, this approach offers a path forward in the face of inherent statistical power constraints and small effect sizes that limit characterization of genetically complex traits in non–model organisms. Our results present an array of candidate genes that may help characterize or profile spongy moth flight capability, and they also highlight potential evolutionary drivers, consequences, or correlated processes characterizing flight evolution within the *Lymantria dispar* subspecies group.

## Methods

### Sample sources and rearing

Adult female spongy moths for all flight analyses in this study were selected from among eight colonies maintained at the US Forest Service quarantine facility in Ansonia, Connecticut (USA). Colonies were originally founded based on egg masses hand–collected from across the global range of ESM and ASM (Table S1, Fig. 1) [34, 35]. Rearing of the colonies was as follows: Eggs were held at 5 ± 1 °C and ~ 100% RH with L:D of 16:8 for 130–150 days to ensure that diapause requirements were met. Within each subspecies, eggs from 100 individual females (ESM 32nd lab generation and ASM 34th lab generation) were then mixed together within each population. From the pool of mixed eggs, we created packets of about 500 eggs. Eggs were incubated at 25 ± 1 °C, 60% RH, and a light:dark schedule of 16:8 h to initiate hatch. Larvae were reared in cohorts of 6–8 under the same conditions as for eggs, using methods described by Keena [125]. A high wheat germ artificial diet [126] was optimized for the individual populations using Wesson salt mix without iron and by adding 0.21 g (ASM population) or 0.13 g (ESM population) amorphous FePO_4_/L of diet [127].

### Genome–wide association study

#### Experimental setup

The flight capability and forewing length GWASs employed females from all colonies above (Fig. 1, Table S1). Adult female moths from Greece were phenotyped for flight capability in 1997 and from all other source locations in 2012–2013, based on a behavioral test protocol described by Keena et al*.* [23, 24]. In brief, recently emerged females were paired with males and were allowed to complete mating 5–45 min prior to their flight test. The flight tests occurred between the hours of 12:00 and 16:00; the early dark phase of the laboratory light cycle (start: 12:00). In nature, females are primed for potential dispersal during this dusk period, immediately prior to egg–laying [34]. Each subject was placed on a vertical bolt of wood (60 cm length, 10 cm width) in a room illuminated at 0.1 lx (conditions that approximate tree perches under natural dusk ambient light levels) [34, 44] and monitored for flight behavior for up to 75 min. Subjects were scored during the test period on a flight behavior scale ranging from 0 to 5: 0, walking only; 1, falling after launch without wing flapping; 2, a gentle fall over < 2 m with vigorous wing flapping; 3, flight with wing flapping for ≥ 2 m but lacking upward displacement; 4, brief but ascending, directed flight; 5, sustained, ascending, directed flight. Following flight assays, each moth was euthanized by freezing and preserved at –20 ℃ until further processing.

We removed the right forewing of adult females and photographed the wings individually. To prepare each wing for imaging, we first dampened the wing with 95% ethanol, which allowed us to spread it without damage on a microscope slide. We then pressed the wing flat beneath a second microscope slide and photographed it using a Dino–lite digital microscope (AnMo Electronics Corporation, New Taipei City, Taiwan). We used ImageJ (v 1.51) [128] to record forewing length from each image, measured as a straight line from the most basal wing vein junction to the outermost vein junction at the wing tip (Fig. S2c).

#### Sequencing and variant calling

We dissected 1–3 legs or thoracic muscle tissue from individual samples and extracted DNA using Qiagen DNeasy Blood and Tissue kits according to manufacturer’s specifications. We assessed DNA quality with a Nanodrop 1000 (Thermo Scientific, Wilmington, DE, USA), DNA concentrations with DNA HS Qubit assays (ThermoFisher), and DNA integrity by manual inspection of agarose gels. Library preparation and genotype–by–sequencing on Ion Torrent sequencers with P1 v3 chips (ThermoFisher) was performed at the Genomic Analysis Platform of IBIS at Université Laval. DNA was sequenced twice—with 150 bp and 225 bp read lengths—to increase the number of available reads.

We used the Fast–GBS pipeline [129, 130] for variant calling, which involved Sabre (v 1.0.0) [131] for demultiplexing, Cutadapt (v 2.3) [132] for read trimming and cleaning, BWA (v 0.7.13) [133] for alignment to the draft ASM genome assembly (v 0.3) [45], SAMtools (v 1.8) [134] for file conversion and indexing, and Platypus (v 0.8.1.1) [135] for variant calling. We imputed missing SNP data from the resulting matrix using Beagle (v 4.1.0) [136], assuming an effective population size of 1000 and running the imputation routine for ten iterations prior to genotype assignment.

We used default quality control settings within the Fast–GBS pipeline, which includes minimum alignment quality (phred = 10) and base calling quality (10) thresholds that are appropriate for Ion Torrent long–read sequence data. We subsequently filtered the Fast–GBS variant calls according to the following criteria: (1) We retained only biallelic SNPs or indels. (2) We discarded loci strongly violating Hardy–Weinberg Equilibrium (*p* < 1 × 10^–6^) in ASM populations (i.e., the ‘control’ [flight–capable] phenotype in the GWAS) in an effort to limit the effect of potential sequencing errors on genotype calls, while minimizing the chance of discarding loci that potentially reflect recent flight–related selection in ESM populations (i.e., the ‘case’ phenotype) [137]. (3) We discarded loci showing the combined conditions of a minor allele frequency below 0.10 globally and below 0.30 in ESM individuals. We applied the 0.10 threshold in order to omit loci contributing little signal to the global dataset. The approximate ESM lower minor allele frequency threshold of 0.30 was included to accommodate the possibility that flight–related directional selection acting on this relatively less replicated test group may produce at least modest allele frequency differences in this group compared to ASM (i.e., allele frequencies substantially above the observed 0.10 global value). (4) We discarded loci showing a global proportion of missing data > 0.5 or a mean depth < 5 reads. (5) We randomly discarded individual loci from locus pairs showing complete linkage disequilibrium (r = 1). (6) We discarded individuals showing > 0.5 proportion of total missing data.

#### GWAS analytical workflow

We expected the geographically widespread population sources for this analysis to exhibit genome–wide differentiation simply due to genetic drift, and potentially also to divergent selection mechanistically unrelated to flight. We could not ensure the elimination of these confounding signals through replicated association tests across populations [138] because most populations showed only narrow variation in flight capability (see Results). Instead, we relied on the expected presence of conflicting signal of non–flight related (i.e., purely geographic) genetic variation across the replicate test populations to help diminish false positive flight associations. We also removed the largest axes of random genetic variation among populations by including in our GWAS models principal components (PCs) of population genetic structure. Finally, to limit false positive associations arising from non–flight related selection, we restricted consideration of detected GWAS candidate genes to top–ranked outliers showing independent functional support for flight relevance from other taxa.

We estimated pairwise kinship across all samples within each geographic location using King (v 2.1.3) [139]. We then used the PC–Air function in the Genesis package (v 2.13.0) [140] in R [141] to estimate global kinship–corrected population structure (using a kinship threshold of 0.025) [142], and the PC–Relate function in Genesis to estimate family–level genetic structure while accounting for population structure [142]. Finally, we incorporated the derived population and kinship factors as fixed effect covariates in separate linear mixed models in base R for female flight capability and forewing length. We used the R packages GWAStools (v 1.40.0) [143] and SNPrelate (v 1.16.0) [144] to prepare the genetic matrix for the models. We explored a range of models, one featuring backwards stepwise selected PCs and several others featuring incrementally increasing numbers of PCs. We then selected a single model to support downstream analyses based on post hoc inspection of model qqplots (created using R package Qqplotr v 0.0.5) [145], Akaike’s information criterion (AIC) values, genomic inflation factor (GIF) values, and p–value distributions across loci. Per–locus *p*–values were calculated based on their individual score test Chi–square statistics. Finally, we used the software Ld–annot (v 0.4) [146] to identify genes in strong linkage disequilibrium (r ≥ 0.90, *p* < 0.05) with GWAS outliers.

### Inbred line analysis

#### Experimental setup

We created the inbred lines by mating one ESM female (source: UC) and one ASM male (source: RM), as well as one ASM female (source: RM) and one ESM male (source: UC) (Fig. 1, Table S1), to produce the initial F_1_ generation. Subsequent generations up to F_4_ consisted of within–generation full–sib single pair crosses of up to five females per egg mass. At each generation, all females were flight tested using the same methods as for the GWASs above and the egg masses of those that showed either minimal flight capability or strong flight capability were used to start the next generation. In the final generation (F_5_) 20 individuals were reared from each F_4_ egg mass and all resulting females were flight tested. We subsequently generated whole–genome sequences for both parental individuals, four females (one F_2_, one F_3_, and two F_5_) exhibiting little or no flight capability (flight score ≤ 1) and relatively small forewing length, and four females (one F_2_, one F_3_, and two F_5_) exhibiting strong flight capability (flight score = 5) and relatively long forewings.

#### Sequencing and variant calling

We isolated DNA from filial samples as described above for GWAS samples. Library synthesis and sequencing for parent samples were performed at the McGill University and Génome Québec Innovation Centre using HiSeqX (Illumina) technology. We prepared libraries using the NEBNextUltraDNA Library Prep Kit for Illumina, according to manufacturer’s instructions with minor modifications. Library concentrations for these samples were measured with DNA HS QuBit assays (ThermoFisher) and assessed for quality with a 2100 Bioanalyzer system (Agilent) using Agilent High Sensitivity DNA Kit LabChips. Samples were then sequenced in the same manner as the parent samples at the McGill University and Génome Québec Innovation Centre [45].

We assessed the quantity and quality of raw sequencing reads using FastQC (v 0.11.2) [147]. Low quality nucleotide calls and sequences (Phred < 30), as well as reads < 30 nucleotides were trimmed from the dataset using Trimmomatic (v 0.36) [148]. Trimmed reads were aligned to the reference ASM genome (v 0.3; 45) using BWA (v 7.13) [133]. Variant detection was carried out with Platypus (v 0.8.1) [135]. As linkage disequilibrium was expected to be high in this inbred line and the sampling was relatively small, this approach required robust and highly informative polymorphisms. For this, we used default Platypus parameters but retained only bi–allelic SNPs and required at least 10 reads per variant. SNPs with very high heterozygosity (> 0.93, possibly indicating paralogous sequences) or a genotyping rate below 70% (relatively less informative) were discarded.

#### Inbred line analytical workflow

Our goal was to identify SNPs co–segregating with flight capability between groups. Assuming a purely additive effect of alleles (no dominance), and given differential flight capability between parental populations, we first selected SNPs that were of opposite homozygous genotypes between parents. We also retained SNPs that were homozygous for one allele in all fully flying individuals (flight score of 5) while being heterozygous or homozygous for the opposite allele in non–flying individuals (flight score of 1 or 2). These steps were performed using in–house python scripts.

### Gene expression analysis

#### Experimental setup

We tested gene expression using individuals from one ESM (source: UC) and one ASM colony (source: RM) (Fig. 1, Table S1). Pupae were separated according to sex and harvested 1, 3, 5, 8 and 11 days after pupation. We chose these time points to capture any extant flight–relevant developmental genetic signal across most of the pupation duration of females (12–13 days) [149]. We spaced the early time points relatively closer together to capture potentially early changes in wing development, as has been observed in *Junonia orithya* pupae [150].

#### RNA sequencing

ESM and ASM female pupae (*n* = 5/sampling point/strain) were homogenized individually in Trizol reagent (Ambion, Life Technologies; 4 to 7 mL depending on the size of the pupa) using 15 mL disposable tissue grinders (VWR, Cat.47732–446). They were then flash frozen in liquid nitrogen prior to further processing. For each sample, 500 µL of the homogenate was transferred to a 1.5 mL microfuge tube, to which another 500 µL of Trizol was added. The tubes were then centrifuged for 4 min at 17,000 × *g* to pellet debris. A 400 µL sample of the supernatant was subsequently used for RNA extraction using the Direct–zol RNA miniprep kit (Zymo Research). RNAs were eluted using 50 µL of water, quantified using a NanoDrop ND1000 spectrophotometer (Thermo Fisher Scientific Inc.) and assessed for quality using a 2100 Bioanalyzer system (Agilent). We used three of the five replicates (60 µL at 10 ng/µL). RNAseq libraries and Ion Proton sequencing were performed at the Genomic Analysis Platform of IBIS (Institute of Integrative and Systems Biology, Laval University, Quebec, QC, Canada) using the NEBNext Ultra II directional RNA library prep kit (New England BioLabs) with the NEBNext Poly (A) mRNA magnetic isolation module. Ion Proton sequencing was performed on an Ion Chef with a P1 chip according to manufacturer's instructions.

Sequencing reads were assessed for quality with FastQC (v 0.11.5) [147] and then trimmed for adapters, quality, and minimum length with Trimmomatic (v 0.36) [147]. Reads were mapped to the draft ASM genome (v 0.3) [45] using HISAT2 (v 2.1.0) [151], SAMtools (v 1.9) [134], and StringTie (v 2.0) [152] by adapting the protocol and scripts described by Pertea et al. [153]. The existing annotations from the draft genome were augmented by merging them with the read mapping information from all samples using StringTie prior to counting reads at each locus with featureCounts (v 2.0.0; counting gene_id meta–features) [154].

#### Differential gene expression analytical workflow

Statistical analyses of the reads mapping to the ASM genome were conducted with the DESeq2 package (v 1.28.1) [155] in R (v 4.0.2), using the apeglm package (v 1.10.0;) [156] for shrinkage estimation. Volcano plots were generated with the EnhancedVolcano package (v 1.6.0) [157]. Heatmaps were generated with the pheatmap package (v 1.0.12) [158] after variance stabilizing transformation of the data.

#### Gene annotation

Protein coding regions for the augmented gene models from StringTie were predicted with Transdecoder (v 5.5.0) [159] using homology searches with BLASTp (blast + v 2.7.1) [160] and the UniProtKB/Swiss–Prot database (v2019_07) [161], and hmmer (v 3.1b2) [162] with Pfam (v 32.0) [163]. We annotated the protein coding regions with BLASTp and the UniProtKB/Swiss–Prot database (v 2019_07) and InterProScan (v 5.36–75.0) [164] analyses.

#### Real–time quantitative PCR (qPCR)

qPCR primers were designed for three spongy moth target genes identified as strongly differentially expressed between the two strains at a given time point (osiris 18, osiris 20, *takeout* with gene IDs Lda.35031, Lda.35510, and Lda.26596, respectively; see Results), and also for four housekeeping genes to use for data normalization (*actin*, *GAPDH*, Ef1g, gamma–Tubulin with gene IDs Lda.11070, Lda.9151, Lda.33417, and Lda.39600, respectively). Prior to primer design, the coding DNA sequences for the above genes were reconstructed based on the ASM (v 3.0; 45) and ESM genome assemblies [47], with manual validation of exon/intron junctions. Primers were designed using Oligo Explorer (v 1.2; Gene Link, NY, USA) and Oligo Analyzer (v 1.2; Gene Link, NY, USA) and manufactured by Integrated DNA Technologies Inc. (Coralville, IA, USA). Primer sequences are provided in Table S13.

For each gene, we used five biological replicates for each tissue sampling time point. cDNA was prepared from 2 µg total RNA using a scaled–up version of the protocol “Reverse transcription with elimination of genomic DNA for quantitative, Real–Time PCR” of the QuantiTect Reverse Transcription kit (Qiagen). 360 µL of Tris–HCl 10 mM, pH 8 was added to the cDNA preparation, for a final volume of 400 µL. The cDNA was stored at –20°C until used for qPCR analysis.

qPCR analyses were conducted using a quantity of cDNA equivalent to 5 ng of total RNA (1 µL of the final RT reaction), using the QuantiTect SYBR Green PCR kit (Qiagen) in a final volume of 10 µL. The five biological– and three technical replicates were run for each sampling point and spongy moth strain. qPCR amplifications were carried out on a 7500 Fast PCR (Applied Biosystems) thermocycler, using 50 cycles of 95°C/15 s, 60°C/30 s and 65°C/90 s. For absolute quantification of target molecules, we used the “linear regression of efficiency” (LRE) method of Rutledge [165], using lambda DNA as a quantitative standard. Copy numbers obtained using this method were then normalized using the GeNorm algorithm [166].

### Gene candidacy

#### Functional themes across all candidate genes

We constructed gene functional networks in the Cytoscape (v 3.8.2) plug–in Bingo [167] to identify GO “Biological process” terms that were enriched within the total spongy moth reference annotation based on the UniProtKB/Swiss–Prot database. To implement flight–targeted functional analyses, we first binned enriched GO terms representing all candidates within each flight analysis into functional categories potentially relevant to sexually dimorphic, temporally variable spongy moth flight. All terms used to characterize each functional category (Table S5) were defined manually and aimed to collectively capture a large proportion of the available GO terms. Search term development and subsequent GO term functional categorization were conducted blind to flight analysis and spongy moth strain. Next, we tested for significant over–representation of individual functional categories within each flight analysis, based on one–tailed binomial tests of GO term counts observed within a given flight analysis compared to those observed in the reference annotation (p_adj_ < 0.05 at FDR 5%). Our null expectation for the test was that observed GO term count within a given functional category occurs in equal proportion to reference annotation GO term counts within that category. Conducting functional assessments within each flight analysis avoided the potentially biased findings that might result from a pooled analysis involving unequal contributions from each flight analysis in terms of biological signal, genetic resolution, statistical power, or candidate gene set size. It also allowed us to define gene sets from each flight analysis individually, requiring only a sample of top–ranked genes from each analysis to submit to enrichment tests.

To explore higher–order functional patterns across enriched candidate genes within each flight analysis, we also used the unsupervised Markov Clustering Algorithm (MCL) within the Cytoscape plug–in clusterMaker [168], accessed via default settings of the plug–in AutoAnnotate [169], to group enriched GO terms into associated clusters.

We also conducted an alternative functional analysis of the gene expression dataset based on transcript matches to NCBI invertebrate RefSeq proteins. We subjected annotations from this approach to a blast2go gene enrichment analysis (v 5.2.5) [170] based on Fisher’s exact test and then compared differentially regulated genes (p_adj_ < 0.05) to all those passing independent filtering in DEseq2 as a reference (strain: up and down separately; strain × day: all).

#### Targeted literature searches for individual gene candidacy

We used two insect–targeted approaches to compare our flight candidates directly with other studies. First, we compared our candidates to those reported in a selection of articles returned by the combined search terms “insect” and “flight”, as well as to genes shown by Mitterboeck et al*.* [50] to exhibit direct signatures of selection associated with flight divergence among insect sister taxa. The search was not comprehensive, but aimed to gauge parallel findings across a range of insect species, flight associated characters, and genetic approaches. We also used the extracted transcripts from our pupal gene expression gene models to identify spongy moth orthologues of: (1) *Drosophila* wing–development related signaling genes reported by Liu et al*.* [75] in the moth *Ostrinia furnacalis* and the locust *Locusta migratoria manilensis*, and; (2) *Drosophila* proteins found by Okada et al*.* [77] to be significantly associated with wing size. We employed the transcripts reported from both studies above as queries for tBLASTx searches (v 2.9.0 +) [159] and then examined the expression patterns of the resulting best matches for statistical significance in our dataset.

To gauge flight–relevant research activity from any taxon in relation to our candidate genes, we scanned Scopus for all peer–reviewed articles published in English between the years 1970–2020 that included in their Title, Keywords, or Abstract both the name of one or more candidate genes in the present study (either a gene code or gene description) and one or more of the potentially flight–relevant search categories noted above (for literature search commands see Additional files). We used a truncated set of our flight–relevant functional category keywords (Table S5) in this search to maximize flight–relevant literature hits while minimizing unrelated hits within the constraints of typical reporting style in the article sections above (Table S3). We then collated search results using the R package bibliometrix [171] and quantified all articles citing each candidate gene. For this search we assumed increasing literature activity reflects positive research evidence of flight relevance.

### Flight capability prediction success of top candidate genes

We estimated the frequency of correct assignment of GWAS samples to binary (“yes/no”) flight capability across moth samples to assess the prediction potential of flight–associated markers from that analysis in the context of a molecular assay. We limited the test to statistically top–ranked markers from the flight capability GWAS that additionally received external functional support for flight candidacy from our sample of the insect flight literature. We constrained ourselves to these loci in order to minimize any confounding geographic association that may have contributed to detection of outliers in that flight analysis. We calculated prediction potential of the loci using a discriminant analysis of principal components (DAPC) [172] in the R package Adegenet [173]. Anderson [174] showed that testing discriminant model prediction success should be limited to samples not used in the model training phase. Accordingly, we first trained the DAPC based on a subset of 100 samples (flight category: 50 “yes”, 50 “no”) randomly selected from across all sample locations (Fig. 1), using cross validation to select the optimal number of principal components within the model to assign flight category membership. We then predicted flight class membership in the remaining samples (60 “yes”, 25 “no”). We replicated the procedure 10 times to estimate mean model prediction success.

### Supplementary Information

**Additional file 1.**

**Additional file 2.**

## Data Availability

Raw sequence reads have been submitted to NCBI SRA under BioSample accessions PRJNA934902 (GWAS). Gene expression data have been submitted to NCBI GEO with series record accession GSE158466. Flight data are provided in the Additional Files of this article. The python script used to identify extreme inbred line flight genotypes is available upon request to the corresponding author.
