# Supplementary material for: Genetics of flight in spongy moths (Lymantria dispar ssp.): functionally integrated profiling of a complex invasive trait
Source: BMC Genomics. 2024 May 31;25:541. doi: 10.1186/s12864-023-09936-8 (PMC11140922; doi:10.1186/s12864-023-09936-8)

# Supporting Information: Genetics of flight in spongy moths (*Lymantria dispar* ssp.): functionally integrated profiling of a complex invasive trait. G. Blackburn *et al*., 2023.

# Table of Contents

[GWAS models 2](#_Toc146918073)

[Literature search commands 2](#_Toc146918074)

[Figure S1. Summary distributions of quality-filtered variants used in GWAS analyses. 10](#_Toc146918075)

[Figure S2. GWAS model setup. 11](#_Toc146918076)

[Figure S3. GWAS model output. 13](#_Toc146918077)

[Figure S4. qPCR output. 16](#_Toc146918078)

[Figure S5. Promoter analysis. 16](#_Toc146918079)

[Figure S6. Candidate gene annotation status across analyses. 28](#_Toc146918080)

[Figure S7. Flight-relevant functional categories represented by MCL functional clusters. 29](#_Toc146918081)

[Figure S8. Taxonomically general literature support. 30](#_Toc146918082)

# GWAS models

Retained loci had 0.106 mean proportion missing data (range: 0–0.502) and 14.9 average mean read depth (range: 5.0–35.7) across individuals. Individuals had 0.106 mean proportion missing data across loci (range: 0.029–0.473). Following missing data imputation, loci had a mean minor allele frequency of 0.216 (range: 0.003–0.500) (Fig. S1).

Principal components (PCs) individually captured modest proportions of global genetic variation (35.49% across the first four PCs) and collectively distinguished each of the sample locations (Fig. S2a). Except for a subset of samples from CR and CN (Table 1), most samples were deemed to be sufficiently distantly related to each other to contribute to the estimation of population structure (total individuals contributing: 113; Fig. S2b).

Flight data (Table S1) were available for all individuals except one from UC and one from RS (n = 292). Flight capability scores were predominantly “0” or “5” across all individuals (Fig. 2). In the Greek colony, individual flight scores were not connected to individual sample identities, but all scores ranged from 0–2 (counts: 28 [score “0”], 10 [“1”], and 1 [“2”]). We therefore binned scores across all populations into “no flight” (flight codes 0–2) or “flight” (flight codes 3–5) categories and modeled the data using a binomial error link. We explored models that included up to 10 PCs to account for geographical structure. These models statistically converged only when employing the kinship matrix with the first PC, two PCs, or four PCs. Performance diagnostics (AIC, GIF, p–value distributions, and quantile–quantile plots) were variable across models (Fig. S3). However, the models employing one or two PCs produced nearly entirely outlying loci (Fig. S3d), probably reflecting lingering effects of random genome-wide geographic structure among populations. We selected the four–PC model for downstream analysis, given that it produced a probability quantile distribution resembling the null expectation of a low proportion of statistical outliers.

Forewings were frequently damaged during storage or processing, and those failing to support accurate measurement were omitted from analyses. Forewing lengths (Table S1) were available for 250 individuals: 31 (UC); 26 (KG); 58 (CJ); 42 (CR); 27 (CN); 30 (RS); 27 (RM), and; 9 (JN). Lengths were approximately normally distributed (Fig. 2) so we modeled them using a Gaussian error link. In general, GWAS models for forewing length that included both the kinship matrix and multiple PCs for population structure exhibited low AIC values, GIF values near one, relatively uniform p–value distributions across loci, and locus quantiles closely matching null predictions (Fig. S3a–d). We chose for downstream analyses output from the forewing length model that included 10 PCs (the highest number of PCs explored) because it had a GIF of approximately 1. However, locus rankings were highly correlated across models with six or more PCs (r ⩾ 0.962; Fig. S3e), as well as between those models and a stepwise model based on PCs 1, 3 and 4 (r ⩾ 0.920).

# Literature search commands

Cognition terms: TITLE-ABS-KEY("cognit*" OR "learn*" OR "memor*")

Growth terms: TITLE-ABS-KEY("develop*" OR "growth" OR "morphogen*")

Metabolism terms: TITLE-ABS-KEY("catab*" OR "metab*" OR "thermogenesis")

Morphology terms: TITLE-ABS-KEY("*limb*" OR "*wing*" OR "anatom*" OR "morphol*" OR "musc*" OR "skelet*")

Movement terms: TITLE-ABS-KEY("*motion" OR "*motor" OR "flight" OR "movement")

Mutation terms: TITLE-ABS-KEY("chromosomal rearrangement*" OR "crossover*" OR "deletion*" OR "frameshift" OR "indel*" OR "insertion*" OR "intron*" OR "inversion*" OR "missense" OR "muta*" OR "nonsense mutation*" OR "null allele*" OR "point mutation*" OR "recombin*" OR "repair*" OR "replicat*" OR "segregat*" OR "substitution*" OR "synonymous" OR "telomer*" OR "translat*" OR "transloc*" OR "transpos*" OR "transversion*")

Neural terms: TITLE-ABS-KEY("nerv*" OR "neur*")

Reproduction terms: TITLE-ABS-KEY("fertil*" OR "reproduc*")

Rhythm terms: TITLE-ABS-KEY("circad*" OR "photoperiodism" OR "rhythm*")

Sexual dimorphism and sexual conflict terms: TITLE-ABS-KEY("antagon*" OR "dimorph*" OR "duplic*" OR "gene dosage" OR "genomic imprint*" OR "modifier*" OR "sex-bias*" OR "sex-determin*" OR "sex-linked" OR "sex-specific" OR "sexual conflict*" OR "splic*")

Sensory terms: TITLE-ABS-KEY("eye*" OR "olfact*" OR "percep*" OR "retina*" OR "sensory" OR "vision" OR "visual*")

Social terms: TITLE-ABS-KEY("aggressi*" OR "breed*" OR "courtship" OR "dispersal" OR "explorat*" OR "mating" OR "migrat*" OR "motivation" OR "social*")

Regulation terms: TITLE-ABS-KEY("channel*" OR "chromatin" OR "epigen*" OR "gene expression" OR "gene silenc*" OR "histone*" OR "hormon*" OR "methyl*" OR "modifier*" OR "receptor*" OR "regulat*" OR "steroid*" OR "transport*")

Source and date parameter terms: LANGUAGE(english) AND PUBYEAR > 1969 AND SRCTYPE(j) AND DOCTYPE(ar) AND SUBJAREA(MEDI OR NURS OR VETE OR DENT OR HEAL OR MULT OR AGRI OR BIOC OR IMMU OR NEUR OR PHAR)

Gene terms: ("Dynein intermediate chain 3, ciliary" OR "DYI3" OR "RNA-directed DNA polymerase from mobile element jockey" OR "RTJK" OR "Retrovirus-related Pol polyprotein from transposon 17.6" OR "POL3" OR "Protein limb expression 1 homolog" OR "LIX1" OR "Dwarfin sma-2" OR "SMA2" OR "Mitochondrial ribonuclease P protein 1 homolog" OR "MRRP1" OR "Nicotinate phosphoribosyltransferase" OR "PNCB" OR "Putative Dol-P-Glc:Glc(2)Man(9)GlcNAc(2)-PP-Dol alpha-1,2-glucosyltransferase" OR "ALG10" OR "Protein kinase C alpha type" OR "KPCA" OR "Mitogen-activated protein kinase kinase kinase kinase 3" OR "M4K3" OR "DNA-dependent protein kinase catalytic subunit" OR "PRKDC" OR "Ribosomal protein S6 kinase alpha-5" OR "KS6A5" OR "Actin-related protein 2" OR "ARP2" OR "Tyrosine-protein kinase-like otk" OR "PTK7" OR "Integrin alpha-3" OR "ITA3" OR "Poly(A) polymerase gamma" OR "PAPOG" OR "Cofilin/actin-depolymerizing factor homolog" OR "CADF" OR "Sex peptide receptor" OR "SPR" OR "Seipin" OR "BSCL2" OR "5-formyltetrahydrofolate cyclo-ligase" OR "MTHFS" OR "MAP/microtubule affinity-regulating kinase 3" OR "MARK3" OR "Serine protease HTRA2, mitochondrial" OR "HTRA2" OR "High affinity cAMP-specific and IBMX-insensitive 3',5'-cyclic phosphodiesterase 8" OR "PDE8" OR "Guanine nucleotide-releasing factor 2" OR "C3G" OR "LINE-1 retrotransposable element ORF2 protein" OR "LORF2" OR "Peroxidase" OR "PERO" OR "Netrin receptor UNC5C" OR "UNC5C" OR "Parafibromin" OR "CDC73" OR "Sus scrofa OX=9823 GN=PMVK PE=1 SV=4" OR "PMVK" OR "Transposon Ty3-I Gag-Pol polyprotein" OR "YI31B" OR "Craniofacial development protein 2" OR "CFDP2" OR "Histamine H2 receptor" OR "HRH2" OR "Plasma membrane calcium-transporting ATPase 2" OR "AT2B2" OR "60S ribosomal protein L35a" OR "RL35A" OR "Probable RNA-directed DNA polymerase from transposon X-element" OR "RTXE" OR "GATA-binding factor A" OR "PNR" OR "Transcription factor BCFI" OR "GATAB" OR "Inducible metalloproteinase inhibitor protein" OR "IMPI" OR "Putative glutamate synthase" OR "GLT1" OR "Guanine nucleotide-binding protein G(q) subunit alpha" OR "GNAQ" OR "Hemolymph lipopolysaccharide-binding protein" OR "LPSBP" OR "Replication factor C subunit 4" OR "RFC4" OR "Tyrosine-protein phosphatase non-receptor type 4" OR "PTN4" OR "Serine/threonine-protein kinase RIO2" OR "RIOK2" OR "Sorbitol dehydrogenase" OR "DHSO" OR "Copia protein" OR "COPIA" OR "Cytochrome P450 6k1" OR "CP6K1" OR "Breast cancer type 1 susceptibility protein homolog" OR "BRCA1" OR "Transcription factor kayak" OR "FOSL" OR "Unc-112-related protein" OR "UN112" OR "Switch 2" OR "CHR9" OR "Zinc phosphodiesterase ELAC protein 2" OR "RNZ2" OR "Serine/threonine-protein kinase LMTK2" OR "LMTK2" OR "UPF0585 protein CG18661" OR "U585" OR "Isatin hydrolase" OR "ISAHY" OR "IQIQ and ubiquitin-like domain-containing protein" OR "IQUB" OR "Putative uncharacterized transposon-derived protein F52C9.6" OR "YSM6" OR "Protein winged eye" OR "WGE" OR "Ribonuclease P/MRP protein subunit POP5" OR "POP5" OR "Rattus norvegicus OX=10116 GN=Egln1 PE=2 SV=2" OR "EGLN1" OR "Fructose-bisphosphate aldolase" OR "ALF" OR "ATP-dependent helicase brm" OR "BRM" OR "Beta-1,3-galactosyltransferase 6" OR "B3GT6" OR "Ferritin subunit" OR "FRI" OR "Microtubule-associated serine/threonine-protein kinase 3" OR "MAST3" OR "Integrator complex subunit 6" OR "INT6" OR "NGFI-RNA-binding protein homolog" OR "NAB" OR "Protein sickie" OR "SICK" OR "Heat shock protein 68" OR "HSP68" OR "ATP-binding cassette sub-family D member 3" OR "ABCD3" OR "Solute carrier family 22 member 3" OR "S22A3" OR "Phosducin-like protein" OR "PHLP" OR "Protein Cep89 homolog" OR "CEP89" OR "Methionine--tRNA ligase, cytoplasmic" OR "SYMC" OR "PIH1 domain-containing protein 1" OR "PIHD1" OR "Nuclear receptor coactivator 6" OR "NCOA6" OR "Male-specific lethal 1 homolog" OR "MSL1" OR "Potassium channel subfamily T member 1" OR "KCNT1" OR "Decapping and exoribonuclease protein" OR "DXO" OR "Allatostatin" OR "ALLS" OR "Inositol 1,4,5-trisphosphate receptor" OR "ITPR" OR "Putative 1-phosphatidylinositol 3-phosphate 5-kinase" OR "FYV1" OR "Protein Gawky" OR "GAWKY" OR "Retrovirus-related Pol polyprotein from transposon TNT 1-94" OR "POLX" OR "Mitogen-activated protein kinase kinase kinase 4" OR "M3K4" OR "Peroxiredoxin-6" OR "PRDX6" OR "NADP-dependent malic enzyme" OR "MAOX" OR "Importin-5" OR "IPO5" OR "RNA polymerase II-associated factor 1 homolog" OR "PAF1" OR "Homeodomain-interacting protein kinase 2" OR "HIPK2" OR "Talin-2" OR "TLN2" OR "Talin-1" OR "TLN1" OR "Neuropeptide CCHamide-2 receptor" OR "CCH2R" OR "Rab GTPase-activating protein 1" OR "RBGP1" OR "Protein obstructor-E" OR "OBSTE" OR "Voltage-dependent calcium channel type A subunit alpha-1" OR "CAC1A" OR "DNA-directed RNA polymerase, mitochondrial" OR "RPOM" OR "Disintegrin and metalloproteinase domain-containing protein 12" OR "ADA12" OR "Multiple C2 and transmembrane domain-containing protein" OR "MCTP" OR "Saccharopine dehydrogenase-like oxidoreductase" OR "SCPDL" OR "Calmodulin" OR "CALM" OR "Glucosidase 2 subunit beta" OR "GLU2B" OR "Synaptic vesicle glycoprotein 2C" OR "SV2C" OR "Regucalcin" OR "RGN" OR "PiggyBac transposable element-derived protein 4" OR "PGBD4" OR "Probable RNA-directed DNA polymerase from transposon BS" OR "RTBS" OR "Transmembrane protein 8B" OR "TMM8B" OR "Selenoprotein M" OR "SELM" OR "Homeobox protein cut" OR "CUT" OR "C-1-tetrahydrofolate synthase, cytoplasmic" OR "C1TC" OR "Zinc finger FYVE domain-containing protein 26 homolog" OR "ZFY26" OR "Probable cardiolipin synthase (CMP-forming)" OR "CRLS1" OR "Probable nuclear hormone receptor HR38 (Fragment)" OR "HR38" OR "Protein mahjong" OR "DCAF1" OR "Transmembrane protein 203" OR "TM203" OR "Solute carrier family 25 member 46" OR "S2546" OR "Facilitated trehalose transporter Tret1-2 homolog" OR "TRE12" OR "Irregular chiasm C-roughest protein" OR "ICCR" OR "Transformation/transcription domain-associated protein" OR "TRRAP" OR "Peroxidasin" OR "PXDN" OR "Transmembrane protein 208" OR "TM208" OR "Importin subunit alpha-7" OR "IMA7" OR "Cytoplasmic dynein 2 heavy chain 1" OR "DYHC2" OR "Fibroblast growth factor receptor homolog 1" OR "FGFR1" OR "AP-2 complex subunit mu" OR "AP2M1" OR "Required for meiotic nuclear division protein 1 homolog" OR "RMND1" OR "Cullin-2" OR "CUL2" OR "Serine/threonine-protein kinase BRSK2" OR "BRSK2" OR "Prefoldin subunit 1" OR "PFD1" OR "Forkhead box protein O" OR "FOXO" OR "Ataxin-2" OR "ATX2" OR "Retinol dehydrogenase 14" OR "RDH14" OR "Superoxide dismutase" OR "SODC" OR "Protocadherin Fat 4" OR "FAT4" OR "Protein abnormal spindle" OR "ASP" OR "Rattus norvegicus OX=10116 GN=Ttpa PE=1 SV=1" OR "TTPA" OR "Ral GTPase-activating protein subunit beta" OR "RLGPB" OR "Bloom syndrome protein homolog" OR "BLM" OR "Protein virilizer" OR "VIR" OR "General transcription factor IIF subunit 1" OR "T2FA" OR "Carbonyl reductase" OR "CBR3" OR "Polynucleotide 5'-hydroxyl-kinase NOL9" OR "NOL9" OR "Ras-like GTP-binding protein Rho1" OR "RHO1" OR "Dipeptidyl aminopeptidase-like protein 6" OR "DPP6" OR "Probable phosphoserine aminotransferase" OR "SERC" OR "Acidic juvenile hormone-suppressible protein 1" OR "AJSP1" OR "Rattus norvegicus OX=10116 GN=Arhgap20 PE=1 SV=2" OR "RHG20" OR "Protein FAM49A" OR "FA49A" OR "Beta-arrestin-1" OR "ARRB1" OR "DNA polymerase eta" OR "POLH" OR "Catalase" OR "CATA" OR "Retrovirus-related Pol polyprotein from transposon 297" OR "POL2" OR "TWiK family of potassium channels protein 18" OR "TWK18" OR "Probable 28S rRNA (cytosine-C(5))-methyltransferase" OR "NSUN5" OR "Organic cation transporter protein" OR "ORCT" OR "Hemicentin-1" OR "HMCN1" OR "Nucleoporin Ndc1" OR "NDC1" OR "Silk gland factor 3" OR "SGF3" OR "Prolyl endopeptidase" OR "PPCE" OR "Superoxide dismutase" OR "SODM1" OR "ATP synthase mitochondrial F1 complex assembly factor 2" OR "ATPF2" OR "Small nuclear ribonucleoprotein-associated protein B" OR "RSMB" OR "tRNA-dihydrouridine(16/17) synthase" OR "DUS1L" OR "Ecdysone-inducible protein E75" OR "E75" OR "Serologically defined colon cancer antigen 8 homolog" OR "SDCG8" OR "Very-long-chain (3R)-3-hydroxyacyl-CoA dehydratase" OR "HACD3" OR "Protein trapped in endoderm-1" OR "GUTR1" OR "UDP-N-acetylglucosamine transporter" OR "S35A3" OR "Probable transaldolase" OR "TALDO" OR "Protein sprint" OR "SPRI" OR "Spliceosome-associated protein CWC27 homolog" OR "CWC27" OR "Ribosome biogenesis protein NOP53" OR "NOP53" OR "Neuropeptide Y receptor type 2" OR "NPY2R" OR "cGMP-dependent protein kinase 1" OR "KGP1" OR "Exonuclease 1" OR "EXO1" OR "Protein bunched, class 2/F/G isoform" OR "BUN2" OR "Uncharacterized aarF domain-containing protein kinase 1" OR "ADCK1" OR "Steroid receptor seven-up, isoforms B/C" OR "7UP1" OR "Allergen Tha p 1" OR "THAP1" OR "ER lumen protein-retaining receptor" OR "ERD2" OR "Wiskott-Aldrich syndrome protein family member 1" OR "WASF1" OR "Protein twisted gastrulation" OR "TSG" OR "Mitotic spindle assembly checkpoint protein MAD1" OR "MD1L1" OR "Synaptic vesicle glycoprotein 2B" OR "SV2B" OR "Chymotrypsin BI" OR "CTRB1" OR "Vinculin" OR "VINC" OR "Macrophage mannose receptor 1" OR "MRC1" OR "Multidrug resistance-associated protein 4" OR "MRP4" OR "Probable sulfite oxidase, mitochondrial" OR "SUOX" OR "Deoxyhypusine hydroxylase" OR "DOHH" OR "Glucose dehydrogenase" OR "DHGL" OR "Rattus norvegicus OX=10116 GN=Ppfia4 PE=1 SV=1" OR "LIPA4" OR "Tensin-1" OR "TENS1" OR "Thyroid adenoma-associated protein homolog" OR "THADA" OR "Kelch domain-containing protein 10 homolog" OR "KLD10" OR "BTB/POPDZ domain-containing protein KCTD3" OR "KCTD3" OR "Dolichyl-diphosphooligosaccharide--protein glycosyltransferase subunit STT3B" OR "STT3B" OR "Endothelin-converting enzyme 1" OR "ECE1" OR "Probable U3 small nucleolar RNA-associated protein 11" OR "UTP11" OR "CDK5 and ABL1 enzyme substrate 2" OR "CABL2" OR "Lactosylceramide 4-alpha-galactosyltransferase (Fragment)" OR "A4GAT" OR "Phosphatidylinositol 4-kinase alpha" OR "PI4KA" OR "Beta-1,3-glucan-binding protein" OR "BGBP" OR "Carnosine N-methyltransferase" OR "CARME" OR "Myrosinase 1" OR "MYRO1" OR "E3 ubiquitin-protein ligase RNF181" OR "RN181" OR "Autophagy protein 12-like" OR "APG12" OR "Facilitated trehalose transporter Tret1" OR "TRET1" OR "Calsyntenin-1" OR "CSTN1" OR "UDP-glucuronic acid decarboxylase 1" OR "UXS1" OR "Neurexin-4" OR "NRX4" OR "Alstrom syndrome protein 1" OR "ALMS1" OR "Protein outspread" OR "OSP" OR "Glucose-6-phosphate 1-dehydrogenase" OR "G6PD" OR "Transposable element P transposase" OR "PELET" OR "Valacyclovir hydrolase" OR "BPHL" OR "Rattus norvegicus OX=10116 GN=Kcnh8 PE=2 SV=2" OR "KCNH8" OR "Rattus norvegicus OX=10116 GN=Ctsh PE=1 SV=1" OR "CATH" OR "Centromere protein X" OR "CENPX" OR "Integrator complex subunit 1" OR "INT1" OR "AMP deaminase 2" OR "AMPD2" OR "Clavesin-1" OR "CLVS1" OR "RNDNA-directed RNA polymerase" OR "RDRP" OR "Anillin" OR "ANLN" OR "Phosphoinositide 3-kinase regulatory subunit 4" OR "PI3R4" OR "Protein groucho" OR "GROU" OR "Acetylcholinesterase" OR "ACES" OR "DNA-directed RNA polymerase III subunit RPC8" OR "RPC8" OR "Intraflagellar transport protein 172 homolog" OR "OSM1" OR "Vacuolar protein sorting-associated protein 13B" OR "VP13B" OR "PAX-interacting protein 1" OR "PAXI1" OR "Transmembrane protease serine 9" OR "TMPS9" OR "Chymotrypsin-2" OR "CTR2" OR "Prostaglandin E2 receptor EP3 subtype" OR "PE2R3" OR "2-oxoglutarate dehydrogenase, mitochondrial" OR "ODO1" OR "Probable multidrug resistance-associated protein lethal(2)03659" OR "L259" OR "Transmembrane emp24 domain-containing protein bai" OR "TMEDA" OR "Zinc transporter 7" OR "ZNT7" OR NA "GFPT1" OR "Chimeric ERCC6-PGBD3 protein" OR "ERPG3" OR "Retrovirus-related Pol polyprotein from transposon 412" OR "POL4" OR "Histone-lysine N-methyltransferase 2D" OR "KMT2D" OR "Receptor-type tyrosine-protein phosphatase N2" OR "PTPR2" OR "Myocardin-related transcription factor B" OR "MRTFB" OR "Nitric oxide synthase-like protein" OR "NOSL" OR "Glyoxylate reductase/hydroxypyruvate reductase" OR "GRHPR" OR "GPI mannosyltransferase 2" OR "PIGV" OR "Dorsal-ventral patterning protein tolloid" OR "TLD" OR "Calpain-D" OR "CAND" OR "Serine hydrolase-like protein" OR "SERHL" OR "Centromere protein J" OR "CENPJ" OR "Glucose-6-phosphate exchanger SLC37A2" OR "G6PT3" OR "Gamma-secretase subunit pen-2" OR "PEN2" OR "SWI/SNF-related matrix-associated actin-dependent regulator of chromatin subfamily B member 1-A" OR "SNF5" OR "Putative serine/threonine-protein kinase haspin homolog" OR "HASP" OR "Phosphatidylinositol N-acetylglucosaminyltransferase subunit P" OR "PIGP" OR "Mitogen-activated protein kinase kinase kinase 9" OR "M3K9" OR "UNC93-like protein" OR "UN93L" OR "Origin recognition complex subunit 3" OR "ORC3" OR "ADP-dependent glucokinase" OR "ADPGK" OR "Lysosomal alpha-mannosidase" OR "MA2B1" OR "Exocyst complex component 7" OR "EXOC7" OR "Ankyrin repeat and fibronectin type-III domain-containing protein 1" OR "ANKF1" OR "Zwei Ig domain protein zig-8" OR "ZIG8" OR "Histone-lysine N-methyltransferase SETMAR" OR "SETMR" OR "Transcription factor BTF3 homolog 4" OR "BT3L4" OR "Transposon TX1 uncharacterized 149 kDa protein" OR "YTX2" OR "A-kinase anchor protein 9" OR "AKAP9" OR "Dynein heavy chain 10, axonemal" OR "DYH10" OR "26S proteasome regulatory subunit 8" OR "PRS8" OR "Fatty acid 2-hydroxylase" OR "FA2H" OR "Neuropathy target esterase sws" OR "SWS" OR "Ornithine decarboxylase" OR "DCOR" OR "Uncharacterized 91 kDa protein in cob intron" OR "YMC6" OR "Phosphatidylinositol-binding clathrin assembly protein LAP" OR "PICAL" OR "Rattus norvegicus OX=10116 GN=Arhgef11 PE=1 SV=1" OR "ARHGB" OR "Solute carrier family 2, facilitated glucose transporter member 3" OR "GTR3" OR "Serine/threonine-protein kinase N2" OR "PKN2" OR "Glucose-induced degradation protein 4 homolog" OR "GID4" OR "Putative nicotine oxidoreductase" OR "NICA" OR "Sex-specific storage-protein 1" OR "SSP1" OR "NUAK family SNF1-like kinase 1" OR "NUAK1" OR "Peptidoglycan-recognition protein LB" OR "PGPLB" OR "TM2 domain-containing protein CG10795" OR "TM2D1" OR "Phosphatidylinositol 4-phosphate 3-kinase C2 domain-containing subunit alpha" OR "P3C2A" OR "Putative ferric-chelate reductase 1 homolog" OR "FRRS1" OR "Peroxisomal acyl-coenzyme A oxidase 3" OR "ACOX3" OR "Structure-specific endonuclease subunit SLX1 homolog" OR "SLX1" OR "Inosine triphosphate pyrophosphatase" OR "ITPA" OR "Cytoplasmic FMR1-interacting protein" OR "CYFIP" OR "Alpha-(1,3)-fucosyltransferase C" OR "FUCTC" OR "Cholinesterase" OR "CHLE" OR "Rattus norvegicus OX=10116 GN=Tmem39a PE=2 SV=1" OR "TM39A" OR "Protein tipE" OR "TIPE" OR "Hydroxyacylglutathione hydrolase, mitochondrial" OR "GLO2" OR "Sodium/potassium-transporting ATPase subunit beta-2" OR "ATPB2" OR "Aldo-keto reductase AKR2E4" OR "AK2E4" OR "Protein stoned-B" OR "STNB" OR "General transcription factor IIH subunit 1" OR "TF2H1" OR "Tyrosine-protein kinase hopscotch" OR "JAK" OR "Nuclear pore complex protein Nup58" OR "NUP58" OR "Tubulin beta-4 chain" OR "TBB4" OR "Dachshund homolog 1" OR "DACH1" OR "Homeobox protein Hox-A1 (Fragment)" OR "HXA1" OR "CCAAT/enhancer-binding protein zeta" OR "CEBPZ" OR "Putative OPA3-like protein CG13603" OR "OPA32" OR "DNA repair protein XRCC3" OR "XRCC3" OR "Digestive organ expansion factor homolog" OR "DIEXF" OR "3'(2'),5'-bisphosphate nucleotidase 1" OR "BPNT1" OR "Protein prenyltransferase alpha subunitWD repeat-containing protein 1-B (Fragment)" OR "PTR1B" OR "Homeotic protein distal-less" OR "DLL" OR "Tight junction-associated protein 1" OR "TJAP1" OR "Multiple inositol polyphosphate phosphatase 1" OR "MINP1" OR "Ras-related protein Rac1" OR "RAC1" OR "Transmembrane protein 132B" OR "T132B" OR "yemanuclein" OR "YEMA" OR "Clathrin interactor 1" OR "EPN4" OR "Rattus norvegicus OX=10116 GN=Shtn1 PE=1 SV=1" OR "SHOT1" OR "Anamorsin homolog" OR "DRE2" OR "Leukocyte tyrosine kinase receptor" OR "LTK" OR "Activin receptor type-1" OR "ACVR1" OR "Calmodulin-like protein 4" OR "CALL4" OR "Toll-like receptor 4" OR "TLR4" OR "Catenin alpha" OR "CTNA" OR "E3 ubiquitin-protein ligase CBL" OR "CBL" OR "Protein 5NUC" OR "5NTD" OR "26S proteasome non-ATPase regulatory subunit 8" OR "PSMD8" OR "Nuclear pore complex protein Nup98-Nup96" OR "NUP98" OR "tRNA pseudouridine synthase-like 1" OR "PUSL1" OR "Rho guanine nucleotide exchange factor 26" OR "ARHGQ" OR "Protein gooseberry-neuro" OR "GSBN" OR "Phenoloxidase-activating enzyme" OR "PPAE" OR "Fumarylacetoacetase" OR "FAAA" OR "Transcription factor HNF-4 homolog" OR "HNF4" OR "Fanconi-associated nuclease 1" OR "FAN1" OR "Protein FAM122A" OR "F122A" OR "Cilia- and flagella-associated protein 44" OR "CFA44" OR "Homeobox protein six1b" OR "SIX1B" OR "Transcription factor MafK" OR "MAFK" OR "Serine/threonine-protein kinase PAK mbt" OR "PAKM" OR "Protein vav" OR "VAV" OR "Actin-binding protein IPP" OR "IPP" OR "Multidrug resistance protein homolog 49" OR "MDR49" OR "Rattus norvegicus OX=10116 GN=Fam98a PE=2 SV=1" OR "FA98A" OR "Rho GTPase-activating protein 190" OR "RG190" OR "Glucose-6-phosphate isomerase" OR "G6PI" OR "Serine/threonine-protein kinase Genghis Khan" OR "GEK" OR "Aryl hydrocarbon receptor" OR "AHR" OR "Serine protease gd" OR "GD" OR "Rattus norvegicus OX=10116 GN=Tpcn1 PE=1 SV=2" OR "TPC1" OR "t-SNARE domain-containing protein 1" OR "TSNA1" OR "RISC-loading complex subunit tarbp2" OR "TRBP2" OR "Cytochrome P450 4C1" OR "CP4C1" OR "Transmembrane 9 superfamily member 3" OR "TM9S3" OR "High mobility group protein DSP1" OR "HMG2" OR "Histone H1" OR "H1" OR "Ceramide-1-phosphate transfer protein" OR "CPTP" OR "Transforming growth factor-beta-induced protein ig-h3" OR "BGH3" OR "Puff-specific protein Bx42" OR "BX42" OR "Putative 115 kDa protein in type-1 retrotransposable element R1DM" OR "Y2R2" OR "Cell death activator CIDE-A" OR "CIDEA" OR "Endoplasmic reticulum-Golgi intermediate compartment protein 2" OR "ERGI2" OR "Tubulin polyglutamylase TTLL4" OR "TTLL4" OR "Hemicentin-2" OR "HMCN2" OR "TBP-related factor" OR "TRF" OR "Rattus norvegicus OX=10116 GN=Chdh PE=1 SV=1" OR "CHDH" OR "Cystathionine beta-synthase" OR "CBS" OR "Syntaxin-5" OR "STX5" OR "Transmembrane protein 179" OR "T179A" OR "DNA transposase THAP9" OR "THAP9" OR "Ras-specific guanine nucleotide-releasing factor 2" OR "RGRF2" OR "Alpha-tocopherol transfer protein-like" OR "TTPAL" OR "Calcium-independent phospholipase A2-gamma" OR "PLPL8" OR "Rattus norvegicus OX=10116 GN=Capn11 PE=2 SV=2" OR "CAN11" OR "Phosphatidylinositol 5-phosphate 4-kinase type-2 beta" OR "PI42B" OR "Sus scrofa OX=9823 GN=PIP4K2A PE=2 SV=1" OR "PI42A" OR "Cyclin-dependent kinase 2" OR "CDK2" OR "Protein-L-isoaspartate(D-aspartate) O-methyltransferase" OR "PIMT" OR "UPF0587 protein GA18326" OR "U587" OR "Transcription elongation factor, mitochondrial" OR "TEFM" OR "Retinal dehydrogenase 1" OR "AL1A1" OR "Protein son of sevenless" OR "SOS" OR "Porphobilinogen deaminase" OR "HEM3" OR "Rattus norvegicus OX=10116 GN=Tomm20 PE=1 SV=2" OR "TOM20" OR "GPI mannosyltransferase 1" OR "PIGM" OR "Calpain-A" OR "CANA" OR "Aquaporin" OR "AQP" OR "Deubiquitinase DESI2" OR "DESI2" OR "Three prime repair exonuclease 2" OR "TREX2" OR "Protein pelota" OR "PELO" OR "Latrophilin Cirl" OR "LPHN" OR "Protein takeout" OR "TAKT" OR "Glutamate receptor ionotropic, kainate 2" OR "GRIK2" OR "Phosphoglycolate phosphatase 2" OR "PGP2" OR "Translocation protein SEC62" OR "SEC62" OR "Probable serine hydrolase" OR "KRAK" OR "Synaptic vesicle glycoprotein 2A" OR "SV2A" OR "Conserved oligomeric Golgi complex subunit 5" OR "COG5" OR "Syntaxin-18" OR "STX18" OR "Retinol-binding protein pinta" OR "PINTA" OR "Charged multivesicular body protein 2a" OR "CHM2A" OR "Probable E3 ubiquitin-protein ligase sinah" OR "SINAL" OR "Alpha-1,3-mannosyl-glycoprotein 2-beta-N-acetylglucosaminyltransferase" OR "MGAT1" OR "Probable DNA mismatch repair protein Msh6" OR "MSH6" OR "60S ribosomal protein L4" OR "RL4" OR "Rattus norvegicus OX=10116 GN=Pex3 PE=1 SV=1" OR "PEX3" OR "Centrobin" OR "CNTRB" OR "E3 ubiquitin-protein ligase SH3RF1" OR "SH3R1" OR "Cytochrome P450 6B4" OR "CP6B4" OR "Esterase E4" OR "ESTE" OR "Isocitrate dehydrogenase" OR "IDH3B" OR "Solute carrier family 46 member 3" OR "S46A3" OR "Monocarboxylate transporter 7" OR "MOT7" OR "Conserved oligomeric Golgi complex subunit 7" OR "COG7" OR "Hexokinase-2" OR "HXK2" OR "Alpha-(1,6)-fucosyltransferase" OR "FUT8" OR "Rattus norvegicus OX=10116 GN=Impact PE=1 SV=1" OR "IMPCT" OR "Transposon Tf2-6 polyprotein" OR "TF26" OR "Bifunctional peptidase and (3S)-lysyl hydroxylase Jmjd7" OR "JMJD7" OR "Actin-like protein 6A" OR "ACL6A" OR "15-hydroxyprostaglandin dehydrogenase" OR "PGDH" OR "Mothers against decapentaplegic homolog 3" OR "SMAD3" OR "Endothelin-converting enzyme homolog" OR "ECE" OR "CLIP-associating protein" OR "CLASP" OR "Probable alpha-aspartyl dipeptidase" OR "PEPE" OR "Visual pigment-like receptor peropsin" OR "OPSX" OR "Collagen alpha-1(XV) chain" OR "COFA1" OR "Pleckstrin homology-like domain family B member 2" OR "PHLB2" OR "Nucleolar complex protein 3 homolog" OR "NOC3L" OR "SID1 transmembrane family member 1" OR "SIDT1" OR "Receptor-type guanylate cyclase Gyc76C" OR "GC76C" OR "Rattus norvegicus OX=10116 GN=Bloc1s5 PE=1 SV=1" OR "BL1S5" OR "Lactase-phlorizin hydrolase" OR "LPH" OR "Protein adenylyltransferase Fic" OR "FICD" OR "5-demethoxyubiquinone hydroxylase, mitochondrial" OR "COQ7")

# Figure S1. Summary distributions of quality-filtered variants used in GWAS analyses.

(a) Site mean proportion missing data; (b) site average mean read depth (c) individual mean proportion missing data; (d) site mean minor allele frequency.

(a) (b) (c) (d)


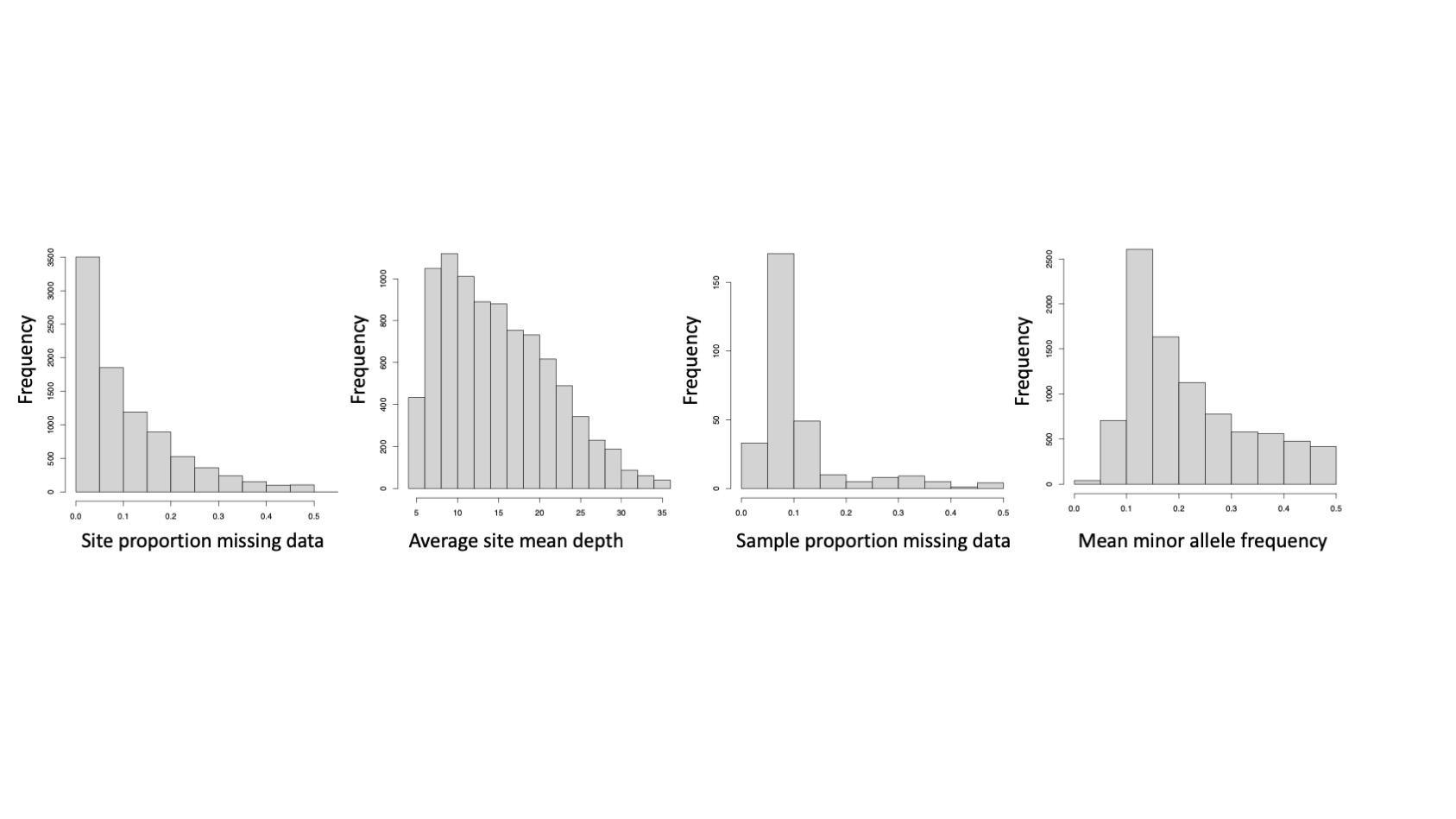


# Figure S2. GWAS model setup.

(a) First six GWAS principle components of population structure, and variance explained across all PCs. (b) GWAS kinship structure within populations (approximate relationships: cousin (above green line); half-sibs (above blue line); siblings (above yellow line); mono-zygotic twins (above red line). (c) Forewing measurement. The two lines indicated were highly correlated across samples. We focused analyses on the shorter of the two measures since it was available across all samples.

(a)


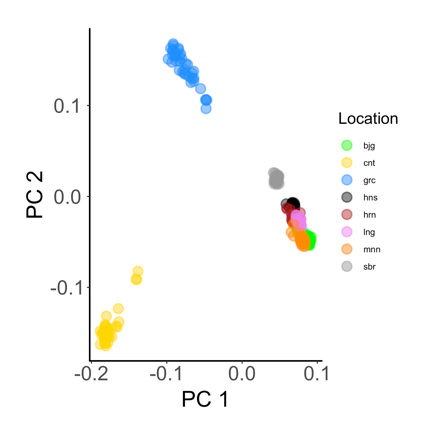

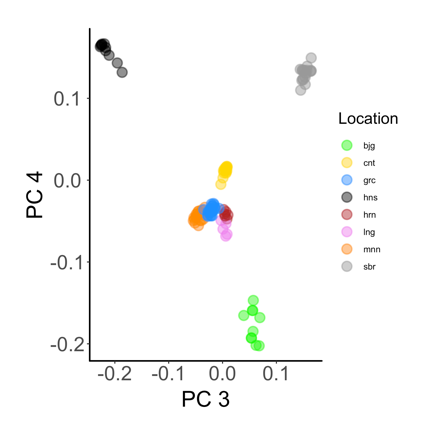

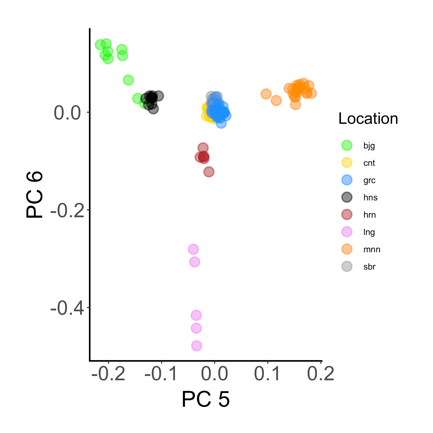

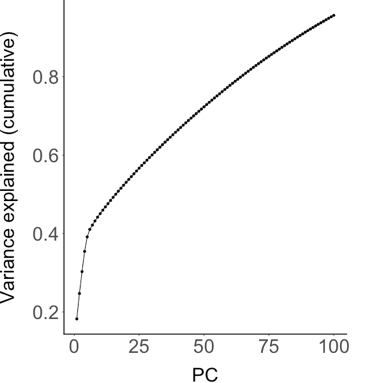


(b)


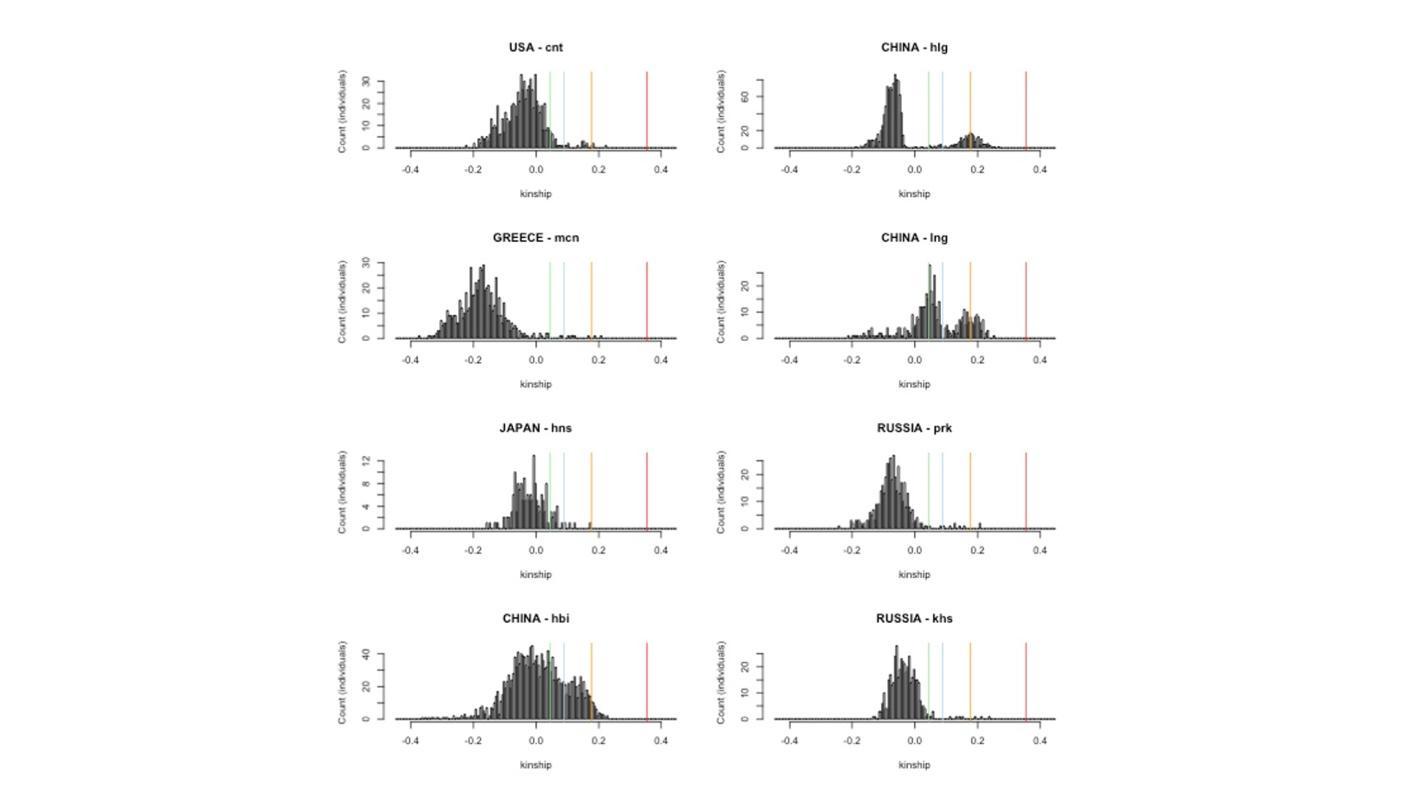


(c)


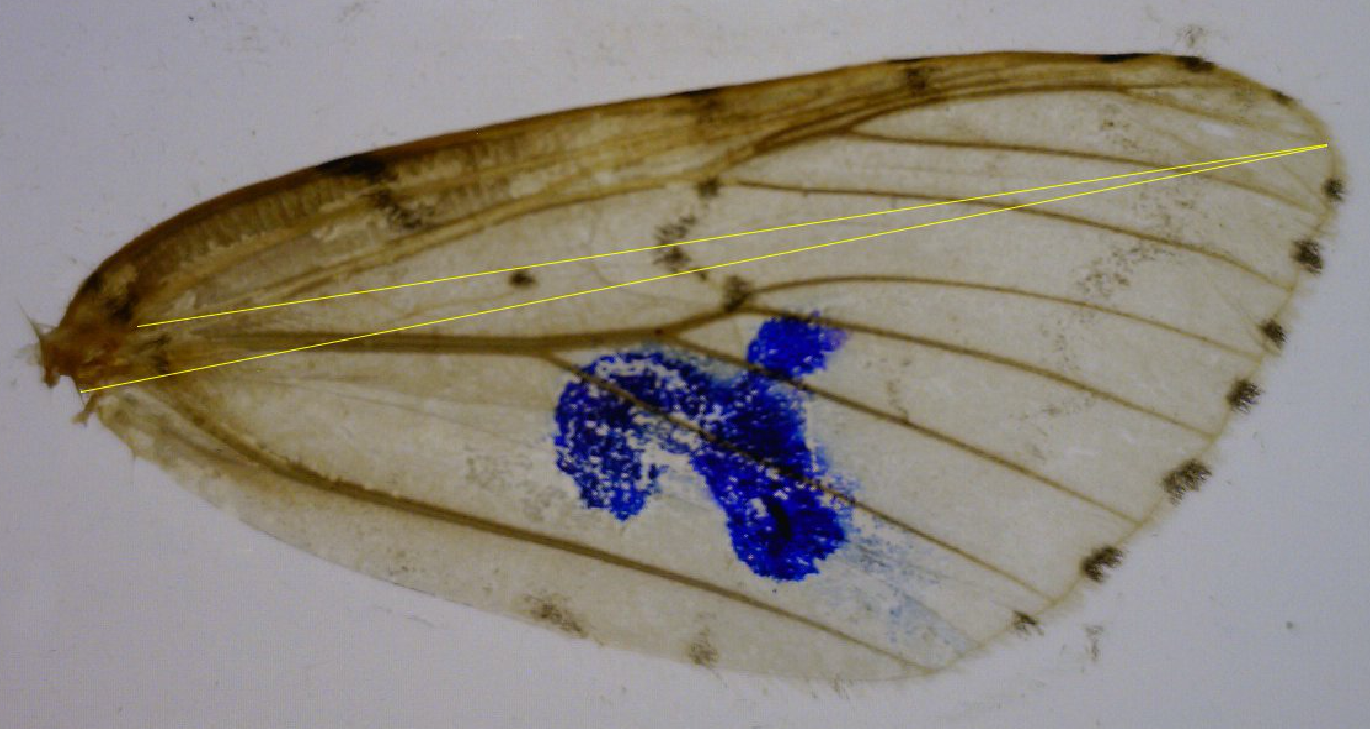


# Figure S3. GWAS model output.

Statistical diagnostics of converged models for flight propensity and forewing length: (a) Genomic inflation factor (b) Akaike’s information criterion (c) p-value distribution (d) quantile-quantile plots (e) pairwise model comparisons of locus probability rankings across loci. In all images “Q” reflects inclusion of PCs of population structure, and “K” reflects inclusion of a kinship matrix. “stepwise” reflects stepwise model choice of an optimum number of population structure PCs (this produced a convergent GWAS model only for the forewing length data, based on the kinship matrix and PCs 1, 3 and 4). Numbers reflect the number of manually included PCs.

(a)

FLIGHT CAPABILITY FOREWING LENGTH


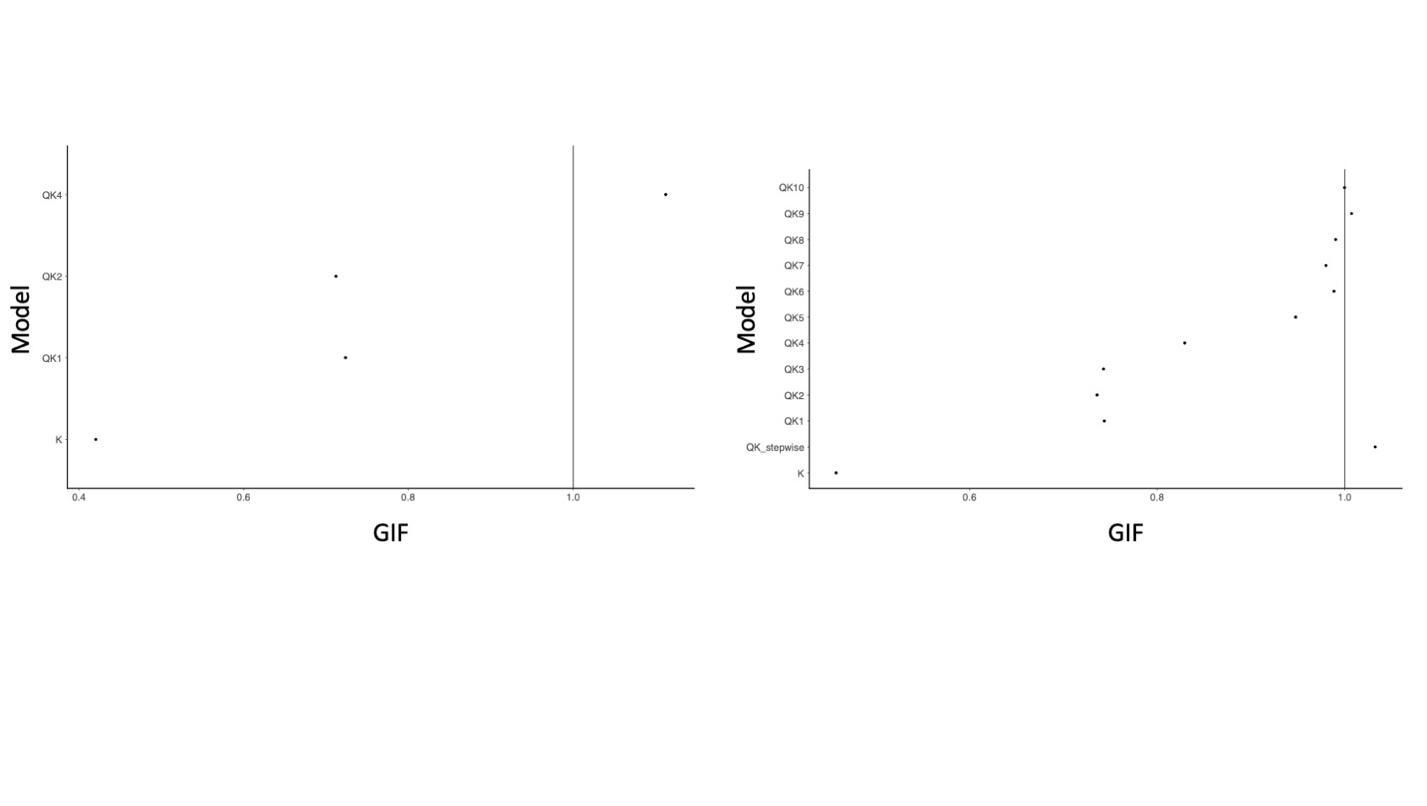


(b)

FLIGHT CAPABILITY FOREWING LENGTH


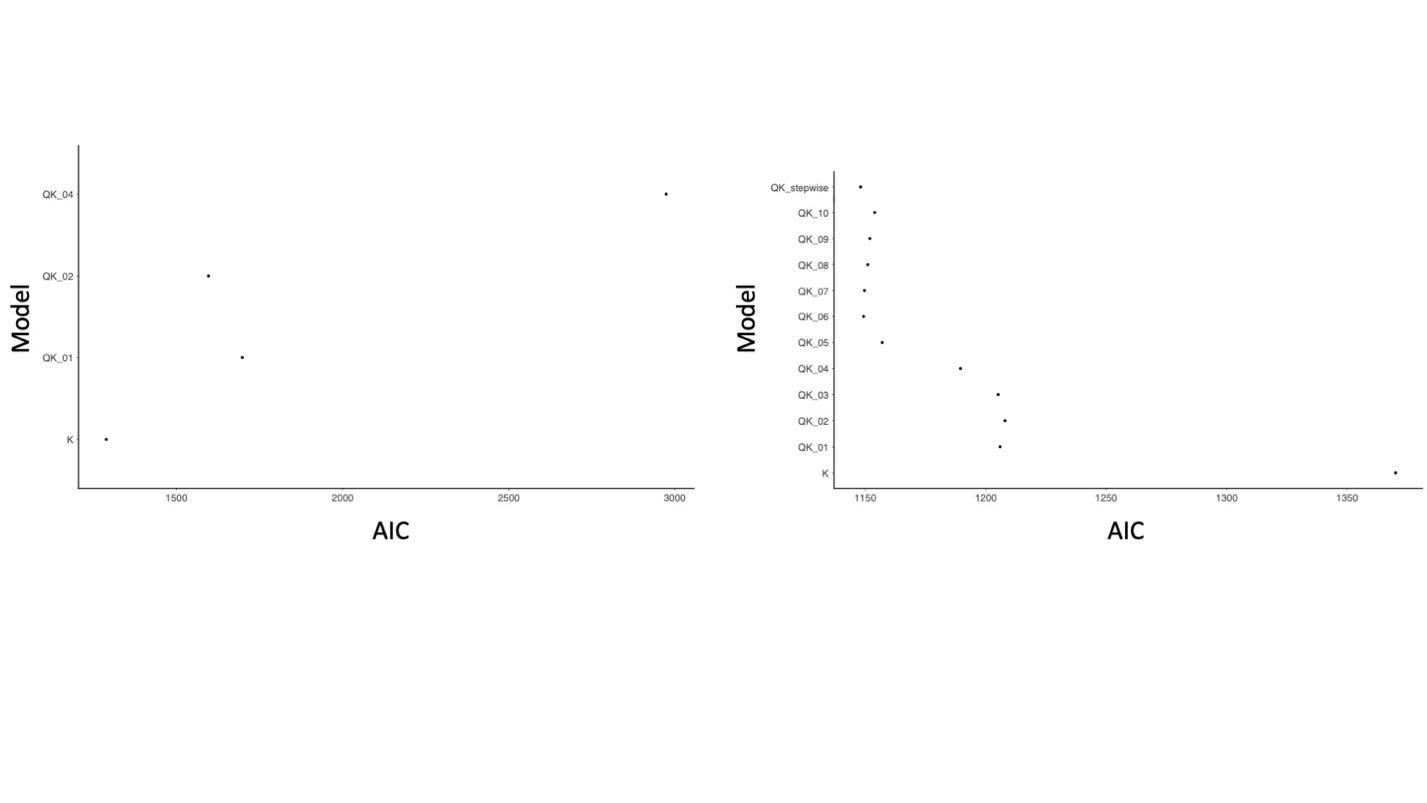


(c)

FLIGHT CAPABILITY


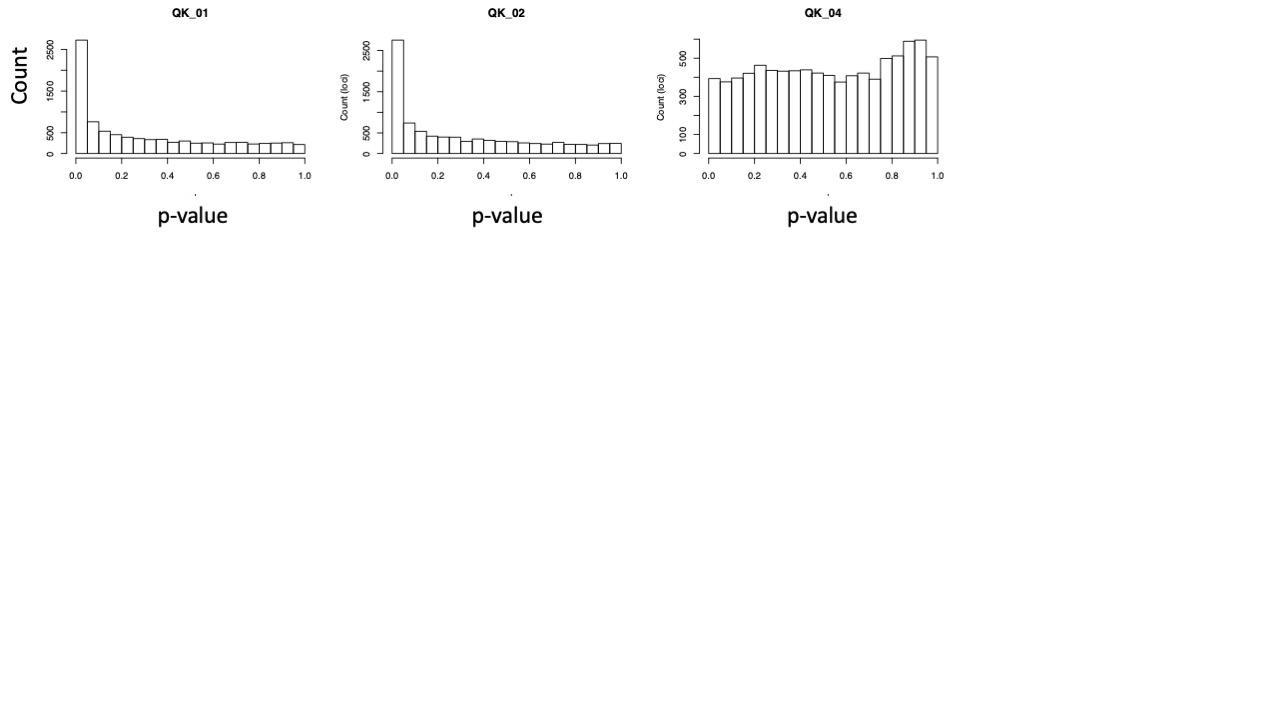


FOREWING LENGTH


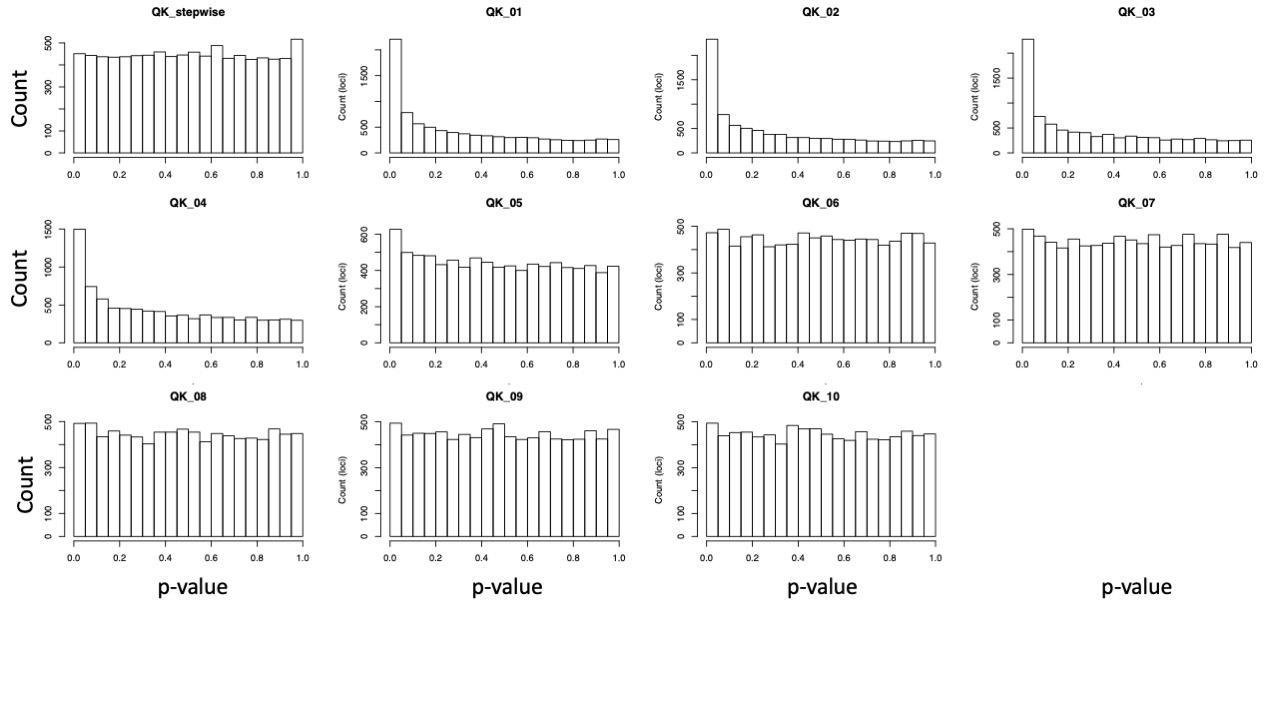


(d)

FLIGHT FOREWING LENGTH

CAPABILITY


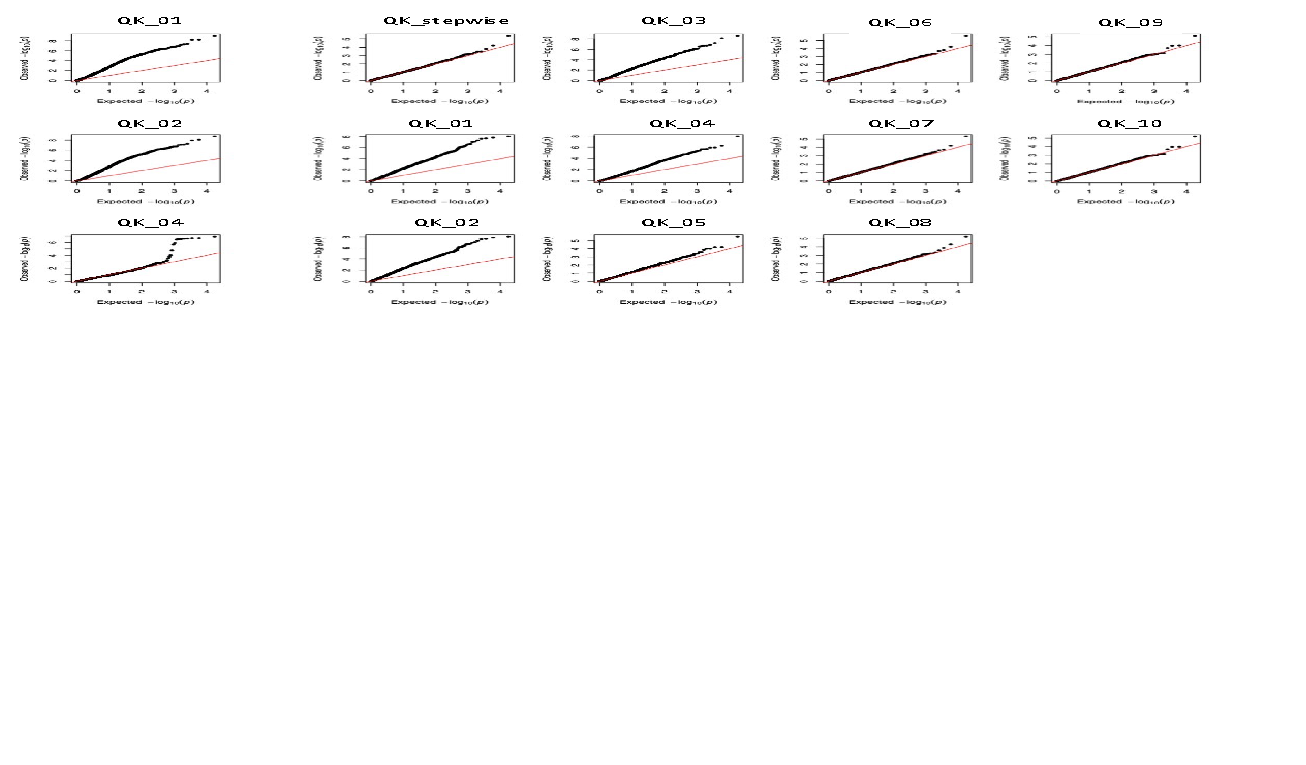


(e)

FLIGHT CAPABILITY FOREWING LENGTH


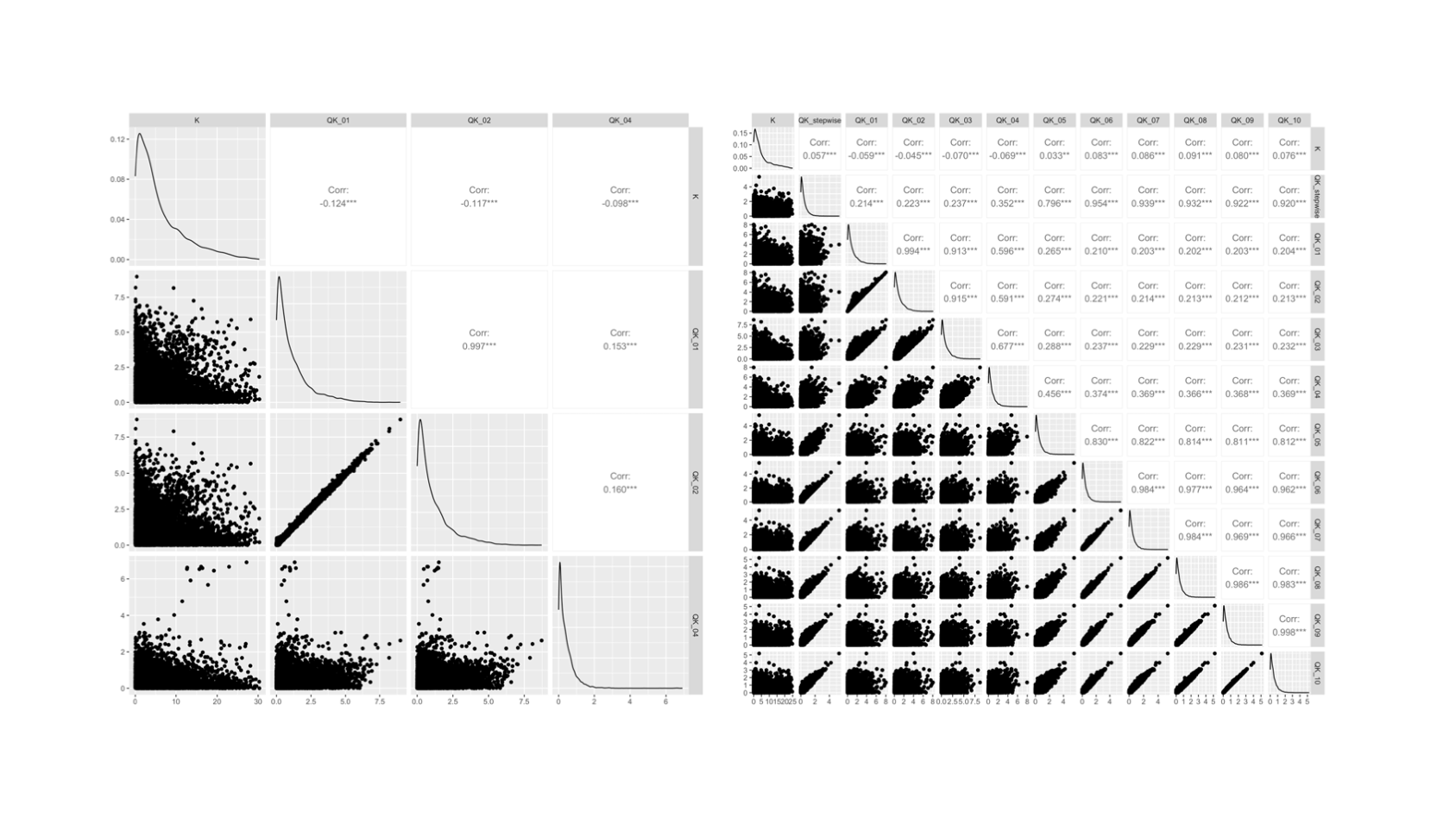


# Figure S4. qPCR output.

**
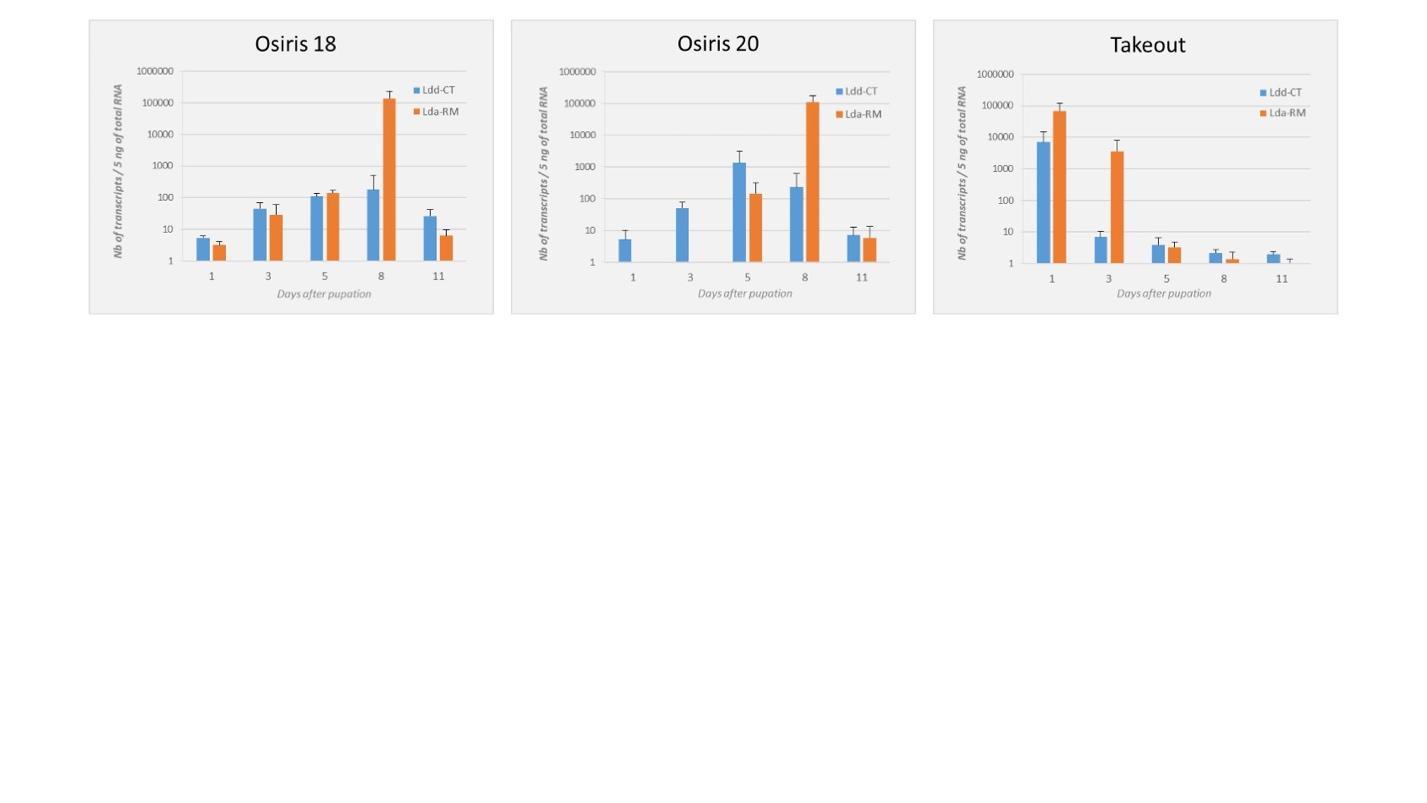
**

# Figure S5. Promoter analysis.

1. Osiris 18.

CLUSTAL O(1.2.4) multiple sequence alignment

Lda_tig00037198_rc_pom_O18 CAATCGCACTATGTTTAGTACAATTTAATATAATATGTATTACTTGTGAGGTCTGTTTTC 60

HiC_scaffold_154_rc_prom_O18 CAATCGCACTATGTTTAGTACAATTTAATATAATATGTATTACTTGTGAGGTCTGTTTTC 60

Ldj_tig00077247_prom_O18 CAATCGCACTATGTTTAGTACAATTTAATATAATATGTATTACTTGTGAGGTCTGTTTTC 60

Lda_tig00037198_rc_pom_O18 ATGCGATTATCAAGTCATTCTGTTTATTTAAAGCATAAAAAATCTTTAATATTGATATAG 120

HiC_scaffold_154_rc_prom_O18 ATGCGATTACCAAGTCATTCTGTTTATTTAAAGCATAAAAAATCTTTAATATTGATATAG 120

Ldj_tig00077247_prom_O18 ATGCGATTACCAAGTCATTCTGTTTATTTAAAGCATAAAAAATCTTTAATATTGATATAG 120

Lda_tig00037198_rc_pom_O18 TAGTATATATAGATAAGAAGTTGTTAGGTTAGTTATTTACGTAATATCTTAAGAAGAAAG 180

HiC_scaffold_154_rc_prom_O18 TAGTATATATAGATAAGAAGTTGTTAGGTTAGTTATTTACGTAATATCTTAAGAAGAAAG 180

Ldj_tig00077247_prom_O18 TAGTATATATAGATAAGAAGTTGTTAGGTTAGTTATTTACGTAATATCTTAAGAAGAAAG 180

Lda_tig00037198_rc_pom_O18 GTCCCTTAAGCAGAGACTGAATGAGTTAACCGACTTGGTATAACATGGATCTCACAAATT 240

HiC_scaffold_154_rc_prom_O18 GTCCCTTAAGCAGAGACTGAATGAGTTAACCGACTTGGTATAACATGGATCTCACAAATT 240

Ldj_tig00077247_prom_O18 GTCCCTTAAGCAGAGACTGAATGAGTTAACCGACTTGGTATAACATGGATCTCACAAATT 240

Lda_tig00037198_rc_pom_O18 TTTATGGTAGAAGAACTCAGTTGCGAGGCTTGGCTTTTTTACAAGTCTTGTTTTTGATGG 300

HiC_scaffold_154_rc_prom_O18 TTTATGGTAGAAGAACTCAGTTGCGAGGCTTGGCTTTTTTACAAGTCTTGTTTTTGATGG 300

Ldj_tig00077247_prom_O18 TTTATGGTAGAAGAACTCAGTTGCGAGGCTTGGCTTTTTTACAAGTCTTGTTTTTGATGG 300

Lda_tig00037198_rc_pom_O18 TATTTGACTTAATCGACGAGAGTCGAATTCATTTAGTTTAGAAACGTTCCACCGATTTTG 360

HiC_scaffold_154_rc_prom_O18 TATTTGACTTAATCGACGAGAGTCGAATTCATTTAGTTTAGAAACGTTCCACCGGTTTTG 360

Ldj_tig00077247_prom_O18 TATTTGACTTAATCGACGAGAGTCGAATTCATTTAGTTTAGAAACGTTCCACCGATTTTG 360

Lda_tig00037198_rc_pom_O18 ATTCCAATCGTGCGCTAGTGTCGCTTTCAAGAGTACCAAATTTGCTCAAATTCAAGCTGC 420

HiC_scaffold_154_rc_prom_O18 ATTCCAATCGTGCGCTAGTGTCGCTTTCAAGAGTACCAAATTTGCTCAAATTCAAGCTGC 420

Ldj_tig00077247_prom_O18 ATTCCAATCGTGCGCTAGTGTCGCTTTCAAGAGTACCAAATTTGCTCAAATTCAAGCTGC 420

Lda_tig00037198_rc_pom_O18 AATTGGATTTAGGATTGGAATCAAGATTCAAGAGTAGGCACACAGGTTGCACACCATCTT 480

HiC_scaffold_154_rc_prom_O18 AATTGGATTTAGGATTGGAATCAAGATTCAAGAGTAGGCACACAGGTTGCACACCATCTT 480

Ldj_tig00077247_prom_O18 AATTGGATTTAGGATTGGAATCAAGATTCAAGAGTAGGCACACAGGTTGCACACCATCTT 480

Lda_tig00037198_rc_pom_O18 TTCTGAGATCCCGGGCGGTTTGAGTAGGATGTGACTGAACCATAGTCTGCCAAAGAAAGG 540

HiC_scaffold_154_rc_prom_O18 TTCTGAGATCCCGGGCGGTTTGAGTAGGATGTGACTGAACCATAGTCTGCCAAAGAAAGG 540

Ldj_tig00077247_prom_O18 TTCTGAGATCCCGGGCGGTTTGAGTAGGATGTGACTGAACCATAGTCTGCCAAAGAAAGG 540

Lda_tig00037198_rc_pom_O18 ACGATAAATTCTATCGATGTTGGCAATTTGCATAACAAGTGCAAAGGATAAAACGGCAGG 600

HiC_scaffold_154_rc_prom_O18 ACGATAAATTCTATCGATGTTGGCAATTTGCATAACAAGTGCAAAGGATAAAACGGCAGG 600

Ldj_tig00077247_prom_O18 ACGATAAATTCTATCGATGTTGGCAATTTGCATAACAAGTGCAAAGGATAAAACGGCAGG 600

Lda_tig00037198_rc_pom_O18 TTTCAAGATCCTATTGCACCTTTACTTTGCTCCAGGTCATACGGAGTGTGATCAAATTAT 660

HiC_scaffold_154_rc_prom_O18 TTTCAAGATCCTATTGCACCTTTACTTTGCTCCAGGTCATACGGAGTGTGATCAAATTAT 660

Ldj_tig00077247_prom_O18 TTTCAAGATCCTATTGCACCTTTACTTTGCTCCAGGTCATACGGAGTGTGATCAAATTAT 660

Lda_tig00037198_rc_pom_O18 AGACAACCTAAAGAAGATAAAGTAAAAATTTTAATGTCGTAGTCATTTGGTAACATTATA 720

HiC_scaffold_154_rc_prom_O18 AGACAACCTAAAGAAGATAAAGTAAAAATTTTAATGTCGTAGTCATTTGGTAACATTATA 720

Ldj_tig00077247_prom_O18 AGACAACCTAAAGAAGATAAAGTAAAAATTTTAATGTCGTAGTCATTTGGTAACATTATA 720

Lda_tig00037198_rc_pom_O18 TTTTTGTGTCTAGTTTGAAATATTGAAAATATATAAAGAACATTTGAGACAATTTAATTT 780

HiC_scaffold_154_rc_prom_O18 TTTTTGTGTCTAGTTTGAAATATTGAAAATATATAAAGAACATTTGAGACAATTTAATTT 780

Ldj_tig00077247_prom_O18 TTTTTGTGTCTAGTTTGAAATATTGAAAATATATAAAGAACATTTGAGACAATTTAATTT 780

Lda_tig00037198_rc_pom_O18 CAGGAAGTGCCGTAATTAAGCTTTCCTAATGCTTAGTTTTTAGTATCATATCTTATGTAC 840

HiC_scaffold_154_rc_prom_O18 CAGGAAGTGCCGTAATTAAGCTTTCCTAATGCTTAGTTTTTAGTATCATATCTTATGTAC 840

Ldj_tig00077247_prom_O18 CAGGAAGTGCCGTAATTAAGCTTTCCTAATGCTTAGTTTTTAGTATCATATCTTATGTAC 840

Lda_tig00037198_rc_pom_O18 GAAATTTCTTATTAGTAATTTTATACTTCATACGTAAGGCCAACCAAATTAATATCTCTG 900

HiC_scaffold_154_rc_prom_O18 GAAATTTCTCATTAGTAATTTTATACTTCATACGTAAGGCCAACCAAATTAATATCTCTG 900

Ldj_tig00077247_prom_O18 GAAATTTCTCATTAGTAATTTTATACTTCATACGTAAGGCCAACCAAATTAATATCTCTG 900

Lda_tig00037198_rc_pom_O18 TTTTCTAAAATAAAGAAACGCAACAATCGAAGTACATTGCCAGTATGATACCTCATAGAA 960

HiC_scaffold_154_rc_prom_O18 TTTTCTAAAATAAAGAAACGCAACAATCGAAGTACATTGCCAGTATGATACCTCATAGAA 960

Ldj_tig00077247_prom_O18 TTTTCTAAAATAAAGAAACGCAACAATCGAAGTACATTGCCAGTATGATACCTCATAGAA 960

Lda_tig00037198_rc_pom_O18 CATCAATATGAGTAATTCAGAATGACACCCCTGATGTGAACTAGCCACTGACCGCTGCAG 1020

HiC_scaffold_154_rc_prom_O18 CATCAATATGAGTAATTCAGAATGACACCCCTGATGTGAACTAGCCACTGACCGCTGCAG 1020

Ldj_tig00077247_prom_O18 CATCAATATGAGTAATTCAGAATGACACCCCTGATGTGAACTAGCCACTGACCGCTGCAG 1020

Lda_tig00037198_rc_pom_O18 TAACTCTCTGTAACAGGCTATTTATATGCGTTTGCCATTTTATCGAGTAATAACATTGTT 1080

HiC_scaffold_154_rc_prom_O18 TAACTCTCTGTAACAGGCTATTTATATGCGTTTGCCATTTTATCGAGTAATAACATTGTT 1080

Ldj_tig00077247_prom_O18 TAACTCTCTGTAACAGGCTATTTATATGCGTTTGCCATTTTATCGAGTAATAACATTGTT 1080

Lda_tig00037198_rc_pom_O18 GTAATGGCTATCCGTTCAATTTGAGATCGTATTCATTTTAATACATACGATTTTGCATAG 1140

HiC_scaffold_154_rc_prom_O18 GTAATGGCTATCCGTTCAATTTGAGATCGTGTTCATTTTAATACATACGATTTTGCATAG 1140

Ldj_tig00077247_prom_O18 GTAATGGCTATCCGTTCAATTTGAGATCGTGTTCATTTTAATACATACGATTTTGCATAG 1140

Lda_tig00037198_rc_pom_O18 ATATCTATTGACTTGCCAATCATTGTGACTTTATAAAAAACCTTTCATATTTGAATCTTT 1200

HiC_scaffold_154_rc_prom_O18 ATATCTATTGACTTGCCAATAATTGTGACTTTATAAAAAACCTTTCATATTTGAATCTTT 1200

Ldj_tig00077247_prom_O18 ATATCTATTGACTTGCCAATAATTGTGACTTTATAAAAAACCTTTCATATTTGAATCTTT 1200

Lda_tig00037198_rc_pom_O18 AAAAATGTATTACGAATTAATATTCGTCATGTAAGTTAAGTATTTATGAAAGAAGAAGTT 1260

HiC_scaffold_154_rc_prom_O18 AAAAATGTATTACGAATTAATATTCGTCATGTAAGTTAAGTATTTATGAAAGAAGAAGTT 1260

Ldj_tig00077247_prom_O18 AAAAATGTATTACGAATTAATATTCGTCATGTAAGTTAAGTATTTATGAAAGAAGAAGTT 1260

Lda_tig00037198_rc_pom_O18 GCGATCTCGTTATTTAGTTATAAAGAAGTATATGTATTTTAGAACAATATGCCGTCGATA 1320

HiC_scaffold_154_rc_prom_O18 GCGATCTCGTTATTTAGTTATAAAGAAGTATATGTATTTTAGAACAATATGCCGTCGATA 1320

Ldj_tig00077247_prom_O18 GCGATCTCGTTATTTAGTTATAAAGAAGTATATGTATTTTAGAACAATATGCCGTCGATA 1320

Lda_tig00037198_rc_pom_O18 CTGAAAAATTAAATGTTTGGCTAATTTTAATATATTTTCATAAACACAAACTTCAGGTGA 1380

HiC_scaffold_154_rc_prom_O18 CTGAAAAATTAAATGTTTGGCTAATTTTAATATATTTTCATAAACACAAACTTCAGGTGA 1380

Ldj_tig00077247_prom_O18 CTGAAAAATTAAATGTTTGGCTAATTTTAATATATTTTCATAAACACAAACTTCAGGTGA 1380

Lda_tig00037198_rc_pom_O18 AGCAAAAGATAGATAAAGGTTAAACTAAAAAATATTTAAACTTTAAATATAGATTATAAA 1440

HiC_scaffold_154_rc_prom_O18 AGCAAAAGATAGATAAAGGTTAAACTAAAAAATATTTAAACTTTAAATATAGATTATAAA 1440

Ldj_tig00077247_prom_O18 AGCAAAAGATAGATAAAGGTTAAACTAAAAAATATTTAAACTTTAAATATAGATTATAAA 1440

Lda_tig00037198_rc_pom_O18 TAAAACCACTTTACGGATTTTATGGTGTTTGTATTTGATTTTTAAATATAGATTAAATTT 1500

HiC_scaffold_154_rc_prom_O18 TAAAACCACTTTACGGATTTTATGGTGTTTGTATTTGATTTTTAAATATAGATTAAATTT 1500

Ldj_tig00077247_prom_O18 TAAAACCACTTTACGGATTTTATGGTGTTTGTATTTGATTTTTAAATATAGATTAAATTT 1500

Lda_tig00037198_rc_pom_O18 GTGTCGAAGAATTAAAAATATTTATAAATGATGCTGCCCATAATCTGAATCTGTTAATAT 1560

HiC_scaffold_154_rc_prom_O18 GTGTCGAAGAATTAAAAATATTTATAAATGATGCTGCCCATAATCTGAATCTGTTAATAT 1560

Ldj_tig00077247_prom_O18 GTGTCGAAGAATTAAAAATATTTATAAATGATGCTGCCCATAATCTGAATCTGTTAATAT 1560

Lda_tig00037198_rc_pom_O18 TAAAAAAAAAACTGCAACTTAAAAATAATTTCTGAAATGCTTGTAACTTTATCACTGAGC 1620

HiC_scaffold_154_rc_prom_O18 TAAAAAAAAAACTGCAACTTAAAAATAATTTCTGAAATGCTTGTAACTTTATCACTGAGC 1620

Ldj_tig00077247_prom_O18 TAAAAAAAAAACTGCAACTTAAAAATAATTTCTGAAATGCTTGTAACTTTATCACTGAGC 1620

Lda_tig00037198_rc_pom_O18 AGTCCGGACACAAGTAACACATAATGACATAAGTGACAGATTGTCTACTATCGATTTCCC 1680

HiC_scaffold_154_rc_prom_O18 AGTCCGGACACAAGTAACACATAATGACATAAGTGACAGATTGTCTACTATCGATTTCCC 1680

Ldj_tig00077247_prom_O18 AGTCCGGACACAAGTAACACATAATGACATAAGTGACAGATTGTCTACTATCGATTTCCC 1680

Lda_tig00037198_rc_pom_O18 TTCGCACATTTCAAGTCCACACGAAGGGAACTCGCTTAGGCAACAAAGTGATAGTTTTCA 1740

HiC_scaffold_154_rc_prom_O18 TTCGCACATTTCAAGTCCACACGAAGGGAACTCGCTTAGGCAACAAAGTGATAGTTTTCA 1740

Ldj_tig00077247_prom_O18 TTCGCACATTTCAAGTCCACACGAAGGGAACTCGCTTAGGCAACAAAGTGATAGTTTTCA 1740

Lda_tig00037198_rc_pom_O18 ATACAGTATGGCGCGCACGCACACATACGCACTCTAATGAATACAATGCACCACATGCGT 1800

HiC_scaffold_154_rc_prom_O18 ATACAGTATGGCGCGCACGCACACATACGCACTCTAATGAATACAATGCACCACATGCGT 1800

Ldj_tig00077247_prom_O18 ATACAGTATGGCGCGCACGCACACATACGCACTCTAATGAATACAATGCACCACATGCGT 1800

Lda_tig00037198_rc_pom_O18 AGGAGCGCAGGCACCACCGAAGATAGTAACCATCGTGCATTCCGATACCACGTCACGTAT 1860

HiC_scaffold_154_rc_prom_O18 AGGAGCGCAGGCACCACCGAAGATAGTAACCATCGTGCATTCCGATACCACGTCACGTAT 1860

Ldj_tig00077247_prom_O18 AGGAGCGCAGGCACCACCGAAGATAGTAACCATCGTGCATTCCGATACCACGTCACGTAT 1860

BREu TATAbox BREd TSS+1 ⌐>

Lda_tig00037198_rc_pom_O18 TAATGCTGGTATATAAACCAAGCCCAGGACTATCCTGGCATACATTGTGATCAGTTACCT 1920

HiC_scaffold_154_rc_prom_O18 TAATGCTGGTATATAAACCAAGCCCAGGACTATCCTGGCATACATTGTGATCAGTTACCT 1920

Ldj_tig00077247_prom_O18 TAATGCTGGTATATAAACCAAGCCCAGGACTATCCTGGCATACATTGTGATCAGTTACCT 1920

Bridge1 DPE/Bridge2

Lda_tig00037198_rc_pom_O18 GGCGGCAGTAAGTCGTCTGCCGTCAAAACATTATCAATTAATTAAGTGAAAAGTGAAAT 1979

HiC_scaffold_154_rc_prom_O18 GGCGGCAGTAAGTCGTCTGCCGTCAAAACATTATCAATTAATTAAGTGAAAAGTGAAAT 1979

Ldj_tig00077247_prom_O18 GGCGGCAGTAAGTCGTCTGCCGTCAAAACATTATCAATTAATTAAGTGAAAAGTGAAAT 1979

| **Element** | **Sequence** | **Score** | **Consensus match** |
| --- | --- | --- | --- |
| GAF motif | AGGAGCGCAG | 0.0296 | NA |
| BRE upstream | AATGCTG | NA | 3/7 |
| TATA box | TATATAAA | 1.000 | 8/8 |
| BRE downstream | CCAAGCC ^1^ | NA | 4/7 |
| lnr/TSS | ACATTG ^2^ | 0.0248 | 4/6 |
| Bridge 1 | GGCGG | 0.0966 | 2/5 |
| DPE | AGTCGT | 0.6301 | 6/6 |
| Bridge 2 | TCGT | 0.0966 | 4/4 |

1. Osiris 20.

CLUSTAL O(1.2.4) multiple sequence alignment

Lda_tig00036830_rc_O20 AATTTATTTAAACATTTAATAGTGTGGATACCCGAATATATTTTATAAACAAAATTA--- 57

Ldd_HiC_scaffold_36236_rc_O20 ------------------------------------------------------------ 0

Ldj_tig00006821_O20 AATTTATTTAAACATTTAATAGTGTGGATACCCGAATATATTTTATAAACAAAATTATAA 60

Lda_tig00036830_rc_O20 ---------TAAACAAATTTAATAACGACTAAATCTCTTTTTAATTGTAACTATTGATAT 108

Ldd_HiC_scaffold_36236_rc_O20 ------------------------------------------------------------ 0

Ldj_tig00006821_O20 TATATTTTATAAACAAATTTAATAACGACTAAATCGCCTTTTAATTGTAACTATTGATAT 120

Lda_tig00036830_rc_O20 -TTTTTTCGCACGTTCACTACAAAGGACCCGTTTGTCATAGCGCTTCAGTTTTAAGGAGT 167

Ldd_HiC_scaffold_36236_rc_O20 ------------------------------------------------------------ 0

Ldj_tig00006821_O20 TTTTTTTCGCACGTTCACTACAAAGGACCCGTTTGTCATAGCGCTTCAGTTTTAAGGAGT 180

Lda_tig00036830_rc_O20 AAGTCTTTAAATTTCCGTTAATATTGGGATCCCCGGCGTCGAAATATCGATTAAAGTCTA 227

Ldd_HiC_scaffold_36236_rc_O20 ------------------------------------------------------------ 0

Ldj_tig00006821_O20 AAGTCTTTAAATTTCCGTTAATATTGGGATCCCCGGCGTCGAAATATCGATTAAAGTCTA 240

Lda_tig00036830_rc_O20 GTTTGGGAGTCACTTAGAGGACAAAGTCAAATTGATAAGCAAAAGCTAGACGTCCTAAAT 287

Ldd_HiC_scaffold_36236_rc_O20 ------------------------------------------------------------ 0

Ldj_tig00006821_O20 GTTTGGGAGTCACTTAGAGGACAAAGTCAAATTGATAAGCAAAAGCTAGGCGTCCTAAAT 300

Lda_tig00036830_rc_O20 AGAGCCAGTCGGTGCTTCACATCGAGACAATGTCTTGTGTTCTATAAAGCACAAATTCGT 347

Ldd_HiC_scaffold_36236_rc_O20 ------------------------------------------------------------ 0

Ldj_tig00006821_O20 AGAGCCAGTCGGTGCTTCACATCGAGACAATGTCTTGTGTTCTATAAAGCACAAATTCGT 360

Lda_tig00036830_rc_O20 CCCCATGTGGATATCTCTGGACAGACACGCCTATATATCAATTAGATCCACTTGACTCCC 407

Ldd_HiC_scaffold_36236_rc_O20 ------------------------------------------------------------ 0

Ldj_tig00006821_O20 CCCCATGTGGATATCTCTGGACAGACACGCCTAAATATCAATTAGATCCACTTGACTCCC 420

Lda_tig00036830_rc_O20 TGCAAATACGTGCTGTTCGAATCGTCGGTGACCCTAATCTGACGGATAGATTGGAGTCGC 467

Ldd_HiC_scaffold_36236_rc_O20 ------------------------------------------------------------ 0

Ldj_tig00006821_O20 TGCAAATACGTGCTGTTCGAATCGTCGGTGACCCTAATCTGACGGATAGATTGGAGTCGC 480

Lda_tig00036830_rc_O20 TGGAGTGACAGAGAGACTTCGGGTCACTCTGTGTGAGTACACACTGGCTCTTGCATGGGG 527

Ldd_HiC_scaffold_36236_rc_O20 ------------------------------------------------------------ 0

Ldj_tig00006821_O20 TGGAGTGACAGAGAGACTTCGGGTCACTCTGTGTGAGTACACACTGGCTCTTGCATGGGG 540

Lda_tig00036830_rc_O20 AATGGTCCTTGCATGGGGAATGAAACTGTTTGCGTTGGTACCACCACTACCATACTACCA 587

Ldd_HiC_scaffold_36236_rc_O20 ------------------------------------------------------------ 0

Ldj_tig00006821_O20 AATGCTCCTTGCATGGGGAATGAAACTGTTTGCGTTGGTACCACCACTACCATACTACCA 600

Lda_tig00036830_rc_O20 ATGTACTATTCGACACTGTAGCAAGGCGGGGTTCATCCATTTCACCTGGAAACATTATGG 647

Ldd_HiC_scaffold_36236_rc_O20 ------------------------------------------------------------ 0

Ldj_tig00006821_O20 ATGTACTATTCGACACTGTAGCAAGGCAGGGTTCATCCATTTCACCTGGAAACATTATGG 660

Lda_tig00036830_rc_O20 TCATTAAATGTATGTTTCCATTGATTATTTCTCCCACGTACATGCAAGATCTGTAATGAA 707

Ldd_HiC_scaffold_36236_rc_O20 ------------------------------------------------------------ 0

Ldj_tig00006821_O20 TCATTAAATGTATGTTTCCATTGATTATTTCTCCCACGTACATGCAAGATCTGTAATGAA 720

Lda_tig00036830_rc_O20 ACACCTGCTGCTATATATCTTGAGCGCTGTTGCATGTCTTTCTTTAAACAAGGTTTAAGA 767

Ldd_HiC_scaffold_36236_rc_O20 ------------------------------------------------------------ 0

Ldj_tig00006821_O20 ACACCTGCTGCTATATATCTTGAGCGCTGTTGCATGTCTTTCTTTAAACAAGGTTTAAGA 780

Lda_tig00036830_rc_O20 AGAGCACTTTCTTCAAGGTACGCAGCAGCCTAGCTATGCCCCTGGTATTACAAAGGT-CC 826

Ldd_HiC_scaffold_36236_rc_O20 ------------------------------------------------------------ 0

Ldj_tig00006821_O20 AGAGCACTTTCTTCAAGGTACGCAGCAGCCTAGCTATGCCCCTGGTATTACAAAGGTCTC 840

Lda_tig00036830_rc_O20 ATGTACGGCAGTATCACTTACCATTAGGTGAGTTGCTAACTCGTTTGGCACCATCAGGCA 886

Ldd_HiC_scaffold_36236_rc_O20 ------------------------------------------------------------ 0

Ldj_tig00006821_O20 ATGTACGGCAGTATCACTTACCATTAGGTGAGTTGCTAACTCGTTTGGCACCATCAGGCA 900

Lda_tig00036830_rc_O20 CCACAAAATCAGTACACGCGCCTGAGCCAGCTCAGGTGTCGACTGCAACTTGAGCCCGAA 946

Ldd_HiC_scaffold_36236_rc_O20 ------------------------------------------------------------ 0

Ldj_tig00006821_O20 CCACAAAATCAGTACACGCGCCTGAGCCAGCTCAGGTGTCGACTGCAACTTGAGCCCGAA 960

Lda_tig00036830_rc_O20 CAGAAGGTCTTCTTCTCTTTGACTAAATAGTGCAATAGCGCTTATGGTGATGTATAAAGA 1006

Ldd_HiC_scaffold_36236_rc_O20 ------------------------------------------------------------ 0

Ldj_tig00006821_O20 CAGAAGGTCTTCTTCTCTTTGACTAAATAGTGCAATAGCACTTATGGTGATGTATAAAGA 1020

Lda_tig00036830_rc_O20 TCAGCATTATTTGAAGACATCCAAAAACATTAAAATTATTCTCATAAATGACAATTTTTG 1066

Ldd_HiC_scaffold_36236_rc_O20 ------------------------------------------------------------ 0

Ldj_tig00006821_O20 TCAGCATTATTTGAAAATATCCAAAAACATTAAAATTATTCTCATAAATGACAATTTTTA 1080

Lda_tig00036830_rc_O20 ATTCTTTTATTTTATTACCCTCATCTCAGCCCATATATGTCCCATTGTTGGGCTCAGGCC 1126

Ldd_HiC_scaffold_36236_rc_O20 ------------------------------------------------------------ 0

Ldj_tig00006821_O20 ATTCTTTTATTTTATTACCCTCATCTCAGCCCATATACGTCCCATTGTTGGGCTCAGGCC 1140

Lda_tig00036830_rc_O20 TTCTCCATTGATCTACTATACCAAGCTGGTCAGATGCATCGGCATAGGGATTTGTATTGA 1186

Ldd_HiC_scaffold_36236_rc_O20 ------------------------------------------------------------ 0

Ldj_tig00006821_O20 TTCTCCATTGACCTACTATACCAAGCTGGTCAGATGCATCGGCATAGGGATTTGTATTGA 1200

Lda_tig00036830_rc_O20 GTTCGCCCATGTTCTCTCATATGGCGAAGGAAAAGAGTACAGCGATCACGACTTCTTCCC 1246

Ldd_HiC_scaffold_36236_rc_O20 ------------------------------------------------------------ 0

Ldj_tig00006821_O20 GTTCGCCCATGTTCTCTCATATGGCGAAGGAAAAGAGTACAGCGATCACGACTTCTTCCC 1260

Lda_tig00036830_rc_O20 TTTCTGAGACATAACCCTGCACTAAGGAAATAATCCGTGTAATGTGGGGGACTTTACAAA 1306

Ldd_HiC_scaffold_36236_rc_O20 ------------------------------------------------------------ 0

Ldj_tig00006821_O20 TTTCTGAGACATAACCCTGCACTAAGGAAATAATCCGTGTAATGTGGGGGACTTTACAAA 1320

Lda_tig00036830_rc_O20 AATATAATTTAACAGACAAAATTCCACCCAGACTTAGAACAACAATCTGTAAAACCCACA 1366

Ldd_HiC_scaffold_36236_rc_O20 ------------------------------------------------------------ 0

Ldj_tig00006821_O20 AATATAATTTAACAGACAAAATTCCACCCAGACTTAGAACAACAATCTGTAAAACCCACA 1380

Lda_tig00036830_rc_O20 AAGTATTGCCTTGCAACCGAACCAACACTGGCATGATAGGTCGGTATCTTAACCAACAAA 1426

Ldd_HiC_scaffold_36236_rc_O20 ------------------------------------------------------------ 0

Ldj_tig00006821_O20 AAGTATTGCCTTGCCATCGAACCAACACTGGCATGGTAGGTCGGTATCTTAACCAACAAA 1440

Lda_tig00036830_rc_O20 CCACTTTGTGCAAATCAAATTGATAGTATAAGAAAGAAGTCATCAATTTTAAGAAAGGCC 1486

Ldd_HiC_scaffold_36236_rc_O20 ------------------------------------------------------------ 0

Ldj_tig00006821_O20 CCACTTTGTGCAAATAAAATTGATAGTATAAGAAAGAAGTCATCAATTTTAAGAAAGGCC 1500

Lda_tig00036830_rc_O20 AATCGTAAAATAAAATCTTTTACCAAACCGTCTAAAGACCATAGTATATTGTAATCTAAT 1546

Ldd_HiC_scaffold_36236_rc_O20 ------------------------------------------------------------ 0

Ldj_tig00006821_O20 AATTGTAAAATAAAATCTTTTACCAAACCGTCTAACGACCATAGTATATTGTAATCTAAT 1560

Lda_tig00036830_rc_O20 TGTAATTTATCGTACTCAACAGTGGAGTGCATATAGGCTAAAGCTAAGAAGAAGAAAAAC 1606

Ldd_HiC_scaffold_36236_rc_O20 ------------------------------------------------------------ 0

Ldj_tig00006821_O20 TGTAATTTACCGTACTCAACAGTGGAGTGCATATAGGCTAAAGCTAAGAAGAAGAAAAAC 1620

Lda_tig00036830_rc_O20 ATACAGAGATATAATTTACTCAGAAAATAATTGTAATTCCTAGAATTATTTCGATCAGTT 1666

Ldd_HiC_scaffold_36236_rc_O20 ------------------------------------------------------------ 0

Ldj_tig00006821_O20 ATACAGAGATATAATTTACTCAGAAAATAATTGTAATTCCTAGAATTATTTCGATCAGTT 1680

Lda_tig00036830_rc_O20 CTTCGTGATCACAACTTCGTTGGATTGAAGTCAGTCGTCAAAGAGTACAACTGTTAGCAA 1726

Ldd_HiC_scaffold_36236_rc_O20 ------------------------------------------------------------ 0

Ldj_tig00006821_O20 CTTCGTGAGCACAACTTCGTTGGATTGAAGTCAGTCGTCAAAGAGTACAACTGTTAGCAA 1740

Lda_tig00036830_rc_O20 GTGAACACATAAACTCAGATTAATATT-TTAATATCATAACCGAAAATCACTCACTCTCA 1785

Ldd_HiC_scaffold_36236_rc_O20 -----------AACTCAGATTAATATTTTTAATATCATAACCGAAAATCACTCACTCTCA 49

Ldj_tig00006821_O20 GTGAACACATAAACTCAGATTAATATTTTTAATATCATAACCGAAAATCACTCACTCTCA 1800

Lda_tig00036830_rc_O20 CCAACAGGACTTTCTTCAATTGAAAATAAATAGTTTGCCAAGTACAAGTATGAAATCCAT 1845

Ldd_HiC_scaffold_36236_rc_O20 CCAACAGGACTTTCTTCAATTGAAAATAAATAGTTTGCCAAGTACAAGTATGAAATCCAT 109

Ldj_tig00006821_O20 CCAACAGGACTTTCTTCAATTGAAAATAAATAGTTTGCCAAGTACAAGTATGAAATCCAT 1860

Lda_tig00036830_rc_O20 TGGTATGCGAATCAGGCTTGCGCATCGCGTGCCTTGCGACCCAAAGGATCACGTATAAAA 1905

Ldd_HiC_scaffold_36236_rc_O20 TGGTATGCGAATCAGGCTTGCGCATCGCGTGCCTTGCGACCCAAAGGATCACGTATAAAA 169

Ldj_tig00006821_O20 TGGTATGCGAATCAGGCTTGCGCATCGCGTGCCTTGCGACCCAAAGGATCACGTATAAAA 1920

Lda_tig00036830_rc_O20 ACCTAGCCCATTGAAAAATTAAATCATTGTTGTTCCACTAACTGGCCGGGCCGTACGTCT 1965

Ldd_HiC_scaffold_36236_rc_O20 ACCTAGCCCATTGAAAAATTAAATCATTGTTGCTCCACTAACTGGCCGGGCCGTACGTCT 229

Ldj_tig00006821_O20 ACCTAGCCCATTGAAAAATTAAATCATTGTTGCTCCACTAACTGGCCGGGCCGTACGTCT 1980

Lda_tig00036830_rc_O20 AATTAATTAAATCTAATGATCAAGTGTTAAATTAAA 2001

Ldd_HiC_scaffold_36236_rc_O20 AATTAATTAAATCTAATGATCAAGTGTTAAATTAAA 265

Ldj_tig00006821_O20 AATTAATTAAATCTAATGATCAAGTGTTAAATTAAA 2016

| **Element** | **Sequence** | **Score** | **Consensus match** |
| --- | --- | --- | --- |
| BRE upstream | GATCACG | NA | 4/7 |
| TATA box | TATAAAAA | 1.0000 | 8/8 |
| BRE downstream | TAGCCCA | NA | 4/7 |
| lnr/TSS | TCATTG | 0.1850 | 5/6 |
| Bridge 1 | TGGCC | 0.4585 | 3/5 |
| DPE | GTACGT | NA | 5/6 |
| Bridge 2 | ACGT | 0.4585 | 4/4 |

(c) Takeout.

CLUSTAL O(1.2.4) multiple sequence alignment

Lda_tig00032100_prom_TO CGAGATGCGAAAATTTATAGCCATGCTGGTGGAATGTATAGTATATATAGACTATGGCAC 60

Ldd_HiC_scaffold_45_rc_prom_TO ------------------------------------------------------------ 0

Ldj_tig00021666_prom_TO CGAGATGCGAAAATTTATAGCCATGCTGGTGGAATGTATAGTATATATAGACTATGGCAC 60

Lda_tig00032100_prom_TO TAACCTATCCTCTCGCAGAAAACTTAAGTCTATGTAGTTTCAATTTGCAAATAAGTTTTA 120

Ldd_HiC_scaffold_45_rc_prom_TO ------------------------------------------------------------ 0

Ldj_tig00021666_prom_TO TAACCTATCCTCTCGCAGGAAACTTAAGTCTATGTAGTTTCAATTTGCAAATAAGTTTTA 120

Lda_tig00032100_prom_TO TTTGGTAAGTGACAGAGCAGGAGGCCCAAACCTACCTTATCTTAAACGTCCATGACCGTT 180

Ldd_HiC_scaffold_45_rc_prom_TO ------------------------------------------------------------ 0

Ldj_tig00021666_prom_TO TTTGGTAAGTGACAGAGCAGGAGGCCCAAACCTACCTTATCTTAAACGTCCATGACCGTT 180

Lda_tig00032100_prom_TO AGTCACTACTTACCATCAGGTTGCATCGTACAAAAAAAATTGTAAACAAATATTATAAAA 240

Ldd_HiC_scaffold_45_rc_prom_TO ------------------------------------------------------------ 0

Ldj_tig00021666_prom_TO AGTCACTACTTACCATCAGGTTACTTCGTTAAAAAAAAATTGTAAACAAATATTGTAAAA 240

Lda_tig00032100_prom_TO AAGTTTCAATATCGTTATTTTTCACATGCTTGGTTATTAGTTGTAAATATGAATTTGTAT 300

Ldd_HiC_scaffold_45_rc_prom_TO ------------------------------------------------------------ 0

Ldj_tig00021666_prom_TO AAGTTTCAATATCATTATTTTTCACATGCTTGGTTATTAGTTGTAAATATGAATTTGTAT 300

Lda_tig00032100_prom_TO CCGCAATTATGATGTCATTAAGTCTTAAACAATGAATATAATTTTAACTCTAAAGGTATA 360

Ldd_HiC_scaffold_45_rc_prom_TO ------------------------------------------------------------ 0

Ldj_tig00021666_prom_TO CCGCAATTATGATGTCATTGAGTCTTAAACAATGAATATAATTTTAACTCTAAAGGTATA 360

Lda_tig00032100_prom_TO ATAAATGCAAATAAGTAATCCGTGTGCATTTGTTAATAAAAAAATAACGATGAACTTGAG 420

Ldd_HiC_scaffold_45_rc_prom_TO --------------------------------------AAAAAATAACGATGAACTTGAA 22

Ldj_tig00021666_prom_TO ATAAATGCAAATAAGTAATCCGTGTGCATTTGTTAATAAAAAAATAACGATGAACTTGAG 420

Lda_tig00032100_prom_TO CGAAAAATGTAATGAAATTGATGTGCCCAGAGATACTCAAAATTAACACAAAAACCATTT 480

Ldd_HiC_scaffold_45_rc_prom_TO CGAAAAATGTAATGAAATTGATGTGCCCAGAGATACTCAAAATTAACACAAAAACCATTT 82

Ldj_tig00021666_prom_TO CGAAAAATGTAATGAAATTGATGTGCCCAGAGATACTCAAAATTAACACAAAAACCATTT 480

Lda_tig00032100_prom_TO TAACGAATACTACTACTTCCTCCATTAAAAATTAGAACTAAATATGTAATAATAATTAAG 540

Ldd_HiC_scaffold_45_rc_prom_TO TAACGAATACTACTACTTCGTACATTAAAAATTAGAACTAAATATGTAATAATAATTAAG 142

Ldj_tig00021666_prom_TO TAACGAATACTACTACTTCGTACATTAAAAATTAGAACTAAATATGTAATAATAATTAAG 540

Lda_tig00032100_prom_TO GTCTAAAATAGTAATGGGATGGTTTGAAAAAGGTTATCTAAAAATATTATCCCTATTCAT 600

Ldd_HiC_scaffold_45_rc_prom_TO GTCTAAAATAGTAATGGGATGGTTTGAAAAAGGTTATCTAAAAATATTATCCCTATTCAT 202

Ldj_tig00021666_prom_TO GTCTAAAATAGTAATGGGATGGTTTGAAAAAGGTTATCTAAAAATATTATCCCTATTCAT 600

da_tig00032100_prom_TO TAATACAACTTGCAAGAATAAATAACTCTCTAATCCCATATAACTATATAGTACTACAAA 660

Ldd_HiC_scaffold_45_rc_prom_TO TAATACAACTTGCAAGAATAAATAACTCTCTAATCCCATATAACTATATAGTACTACAAA 262

Ldj_tig00021666_prom_TO TAATACAACTTGCAAGAATAAATAACTCTCTAATCCCATATAACTATATAGTACTACAAA 660

Lda_tig00032100_prom_TO GTTATATTTGCAGCTAACGAACCAGTGTACCTGTGTTCAAAGTTAATTTGTAAAATGGTC 720

Ldd_HiC_scaffold_45_rc_prom_TO GTTATATTTGCAGCTAACGAACCAGTGTACCTGTGTTCAAAGTTAATTTGTAAAATGGTC 322

Ldj_tig00021666_prom_TO GTTATATTTGCAGCTAACGAACCAGTGTACCTGTGTTCAAAGTTAATTTGTAAAATGGTC 720

Lda_tig00032100_prom_TO AATCAACGAAATAAAAATGCAAGACTTAGAATAAGACTATCGCTAAGCATTATGTAGCGA 780

Ldd_HiC_scaffold_45_rc_prom_TO AATCAACGAAATAAAAATGCAAGACTTAGAATAAGACTATCGCTAAGCATTATGTAGCGA 382

Ldj_tig00021666_prom_TO AATCAACGAAATAAAAATGCAAGACTTAGAATAAGACTATCGCTAAGCATTATGTAGCGA 780

Lda_tig00032100_prom_TO AATTTTCCGAAATTGATATGAAAGTAGGTTTCTACCTACAAAAAGAAATTAGATGCCATC 840

Ldd_HiC_scaffold_45_rc_prom_TO AATTTTCCGAAATTGATATGAAAGTAGGTTTCTACCTACAAAAAGAAATGAGATGCCATC 442

Ldj_tig00021666_prom_TO AATTTTCCGAAATTGATATGAAAGTAGGTTTCTACCTACAAAAAGAAATGAGATGCCATC 840

Lda_tig00032100_prom_TO AAAAATATAATTGTACTAGCTGACCCAACAGACGTTGTTCTGTTCAAATAAAGCGCCACC 900

Ldd_HiC_scaffold_45_rc_prom_TO AAAAATATAATTGTACTAACTGACCCAACAGACGTTGTTCTGTTCAAATAAAGCGCCACC 502

Ldj_tig00021666_prom_TO AAAAATATAATTGTACTAGCTGACCCAACAGACGTTGTTCTGTTCAAATAAAGCGCCACC 900

Lda_tig00032100_prom_TO TACCGGGTCCAATTGTGAATCTAAACCATTCTCGAATCTCATTGAGCACACAC--AAAAA 958

Ldd_HiC_scaffold_45_rc_prom_TO TACCGGGTCCAATTGTGAATCTAAACCATTCTCGAATCTCATTGAGCACACACAAAAAAA 562

Ldj_tig00021666_prom_TO TACCGGGTCCAATTGTGGATCTAAACCATTCTCGAATCTCATTGAGCACACACAAAAAAA 960

Lda_tig00032100_prom_TO ATTACTTAAATTACTTAAATCCAGTCAACAGATATCGCCATCTGTTAGAATCTTTTGGAG 1018

Ldd_HiC_scaffold_45_rc_prom_TO ATTACTTAAATTACTTAAATCCAGTCAACAGATATCGCCATCTGTTAGAATCTTTTGGAG 622

Ldj_tig00021666_prom_TO ATTACTTAAATTACTTAAATCCAGTCAACAGATATCGCCATCTGTTAGAATCTTTTGGAG 1020

Lda_tig00032100_prom_TO TTAACAGATAATTGTGACTGTCAATTAATTATAGACAAATAATTTGTAATAAAATAAAAT 1078

Ldd_HiC_scaffold_45_rc_prom_TO TTAACAGATAATTGTGACTGTCAATTAATTATAGACAAATAATTTGTAATAAAATAAAAT 682

Ldj_tig00021666_prom_TO TTAACAGATAATTGTGACTGTCAATTAATTATAGACAAATAATTTGTAATAAAATAAAAT 1080

Lda_tig00032100_prom_TO TGCGACTATAATTAAAGATTTAAGCTATCCTATGTTTCAAGTTGGAGCAAACTGCACACG 1138

Ldd_HiC_scaffold_45_rc_prom_TO TGCGACTATAATTAAAGATTTAAGCTATCCTATGTTTCAAGTTGGAGCAAACTGCACACG 742

Ldj_tig00021666_prom_TO TGCGACTATAATTAAAGATTTAAGCTATCCTATGTTTCAAGTTGGAGCAAACTGCACACG 1140

Lda_tig00032100_prom_TO GTGTGCAAATTTGATTAAAATCGGTTAAGTAGTTTAGGAGTCCATCGCGGACAAACATCG 1198

Ldd_HiC_scaffold_45_rc_prom_TO GTGTGCAAATTTGATTAAAATCGGTTAAGTAGTTTAGGAGTCCATCGCGGACAAACATCG 802

Ldj_tig00021666_prom_TO GTGTGCAAATTTGATTAAAATCGGTTAAGTAGTTTAGGAGTCCATCGCGGACAAACATCG 1200

Lda_tig00032100_prom_TO TGACAGGAGATTTATATATATTAAGATTACAACAAAGCAGATTTTTAATATTGATATGAT 1258

Ldd_HiC_scaffold_45_rc_prom_TO TGACAGGAGATTTATATATATTAAGATTACAACAAAACAGATTTTTAATATTGATATGAT 862

Ldj_tig00021666_prom_TO TGACAGGAGATTTATATATATTAAGATTACAACAAAACAGATTTTTAATATTGATATGAT 1260

**Element 1:**

TATAbox BREd +1 ⌐>

Lda_tig00032100_prom_TO CATTATAAGATGTTATTAAGTTTGCTAGTTACGTAATAACTTCAGACAAAAGGTCCTTTA 1318

Ldd_HiC_scaffold_45_rc_prom_TO CATTATAAGATGTTATTAAGTTTGCTAGTTACGTAATAACTTCAGACAAAAGGTCCTTTA 922

Ldj_tig00021666_prom_TO CATTATAAGATGTTATTAAGTTTTCTAGTTACGTAATAACTTCAGACAAAAGGTCCTTTA 1320

Bridge1 Bridge2/DPE

Lda_tig00032100_prom_TO AGTAGGGACTGAATGAATTAACTGACTTGATGTTTTAAGGATATCACAACTTTTTACGGT 1378

Ldd_HiC_scaffold_45_rc_prom_TO AGTAGGGACTGAATGAATTAACTGACTTGATGTTTTAAGGATATCACAACTTTTTACGGT 982

Ldj_tig00021666_prom_TO AGTAGGGACTGAATGAATTAACTGACTTGATGTTTTAAGGATATCACAACTTTTTACGGT 1380

Lda_tig00032100_prom_TO AGAAGATCTATCTTTGCGAGACTTGTTTTTTTTTAGTTATGTTTTTCATGGTATCCCTAT 1438

Ldd_HiC_scaffold_45_rc_prom_TO AGAAGATCTAAGT-TGCGAGACTTGTTTTTTTTTAGTTATGTTTTTCATGGTATCCCTAT 1041

Ldj_tig00021666_prom_TO AGAAGATCTAAGT-TGCGAGACTTGTTTTTTTTTAGTTATGTTTTTCATGGTATCCCTAT 1439

Lda_tig00032100_prom_TO AAAACTAAAATATAAGAATAAATACTAAAGTTATCACACAGCCTTGTCTGAGAGTGCAGG 1498

Ldd_HiC_scaffold_45_rc_prom_TO AAAACTAAAATATAAGAATAAATACTAAAGTTATCACACAGCCTTGTCTGAGAGTGCAAG 1101

Ldj_tig00021666_prom_TO AAAACTAAAATATAAGAATAAATACTAAAGTTATCACACAGCCTTGTCTGAGAGTGCAGG 1499

Lda_tig00032100_prom_TO TGGTTGGGCCAGAAGGCACTTCATCCCCAGTAGGAGGCTGAACCTGTTAATATGATTAAA 1558

Ldd_HiC_scaffold_45_rc_prom_TO TGGTTGGGCCAGAAGGCACTTCATCCCCAGTAGGAGGCTGAACCTGTTAATATGATTAAA 1161

Ldj_tig00021666_prom_TO TGGTTGGGCCAGAAGGCACTTCATCCCCAGTAGGAGGCTGAACCTGTTAATATGATTAAA 1559

Lda_tig00032100_prom_TO AAAACCTTCACCTAACTTAAAACAAAAACTCTTCTACAATAGATAAAACAAACCGCCCTT 1618

Ldd_HiC_scaffold_45_rc_prom_TO AAAACCTTCACCTAACTTAAAACAAAAACTCTTCTACAATAGATAAAACAAACCGCCCTT 1221

Ldj_tig00021666_prom_TO AAAACCTTCACCTAACTTAAAACAAAAACTCTTCTACAATAGATAAAACAAACCGCCCTT 1619

Lda_tig00032100_prom_TO TTTTGCAGGCTATGGTGTGGTCGCACGCCGTCTGTTCCGTCCGGGGCGATGAAAGAGCGG 1678

Ldd_HiC_scaffold_45_rc_prom_TO TTTTGCAGGCTATGGTGTGGTCGCACGCCGTCTGTTCCGTCCGGGGCGATGAAAGAGCGG 1281

Ldj_tig00021666_prom_TO TTTTGCAGGCTATGGTGTGGTCGCACGCCGTCTGTTCCGTCCGGGGCGATGAAAGAGCGG 1679

Lda_tig00032100_prom_TO CGGCCGTCTTATACCCGCAGTGTTCCCGCCCCATCGTCATTTAACAGACAGCTGGTATCG 1738

Ldd_HiC_scaffold_45_rc_prom_TO CGGCCGTCTTATACCCGCAGTGTTCCCGCCCCATCGTCATTTAACAGACAGCTGGTATCG 1341

Ldj_tig00021666_prom_TO CGGCCGTCTTATACCCGCAGTGTTCCCGCCCCATCGTCATTTAACAGACAGCTGGTATCG 1739

Lda_tig00032100_prom_TO AACCCTTGACATCCGTGGCTCTACGGAACGCTTGACTTCATATGCAAGTTGATCCGTCTC 1798

Ldd_HiC_scaffold_45_rc_prom_TO AACCCTTGACATCCGTGGCTCTACGGAACGCTTGACTTCATATGCAAGTTGATCCGACTC 1401

Ldj_tig00021666_prom_TO AACCCTTGACATCCGTGGCTCTACGGAACGCTTGACTTCATATGCAAGTTGATCCGTCTC 1799

Lda_tig00032100_prom_TO TCTGAGATACATCGTATCCATTTACTCAGACGTAATTTTTATTCCTTTTTAAGTACCGAC 1858

Ldd_HiC_scaffold_45_rc_prom_TO TCTGAGATACATCGTATCCATTTACTCAGACGTAATTTTTATTCCTTTTTAAGTACCGAC 1461

Ldj_tig00021666_prom_TO TCTGAGATACATCGTATCCATTTACTCAGACGTAATTTTTATTCCTTTTTAAGTACCGAC 1859

Lda_tig00032100_prom_TO GGTCTGTTTCAGTAAAAACAGATAGGTATATATAAAAAACTCATTTCAATTAACATTTTT 1918

Ldd_HiC_scaffold_45_rc_prom_TO GGTCTGTTTCAGTAAAAACAGATAGGTATATATAAAAAACTCATTTCAATTAACATTTTT 1521

Ldj_tig00021666_prom_TO GGTCTGTTTCAGTAA-AACAGATAGGTATAtataaaAAACTCATTTCAATTAACATTTTT 1918

**Element 2:**

BREu TATA box BREd +1 ⌐>

Lda_tig00032100_prom_TO AATTAATAATTTCCACATGAGTATATAAATGGTTAAAGATTGCAATAATCATTCGCAAGT 1978

Ldd_HiC_scaffold_45_rc_prom_TO AATTAATAATTTCCACACGAGTATATAAATGGTTAAAGATTGCAATAATCATTCGCAAGT 1581

Ldj_tig00021666_prom_TO AATTAATAATTTCCACACGAGTAtataaaTGGTTAAAGATTGCAATAATCATTCGCAAGT 1978

Bridge1 Bridge2/DPE

Lda_tig00032100_prom_TO TGCGTATGTACTTTGAGGTATAATACACGTTAATC 2013

Ldd_HiC_scaffold_45_rc_prom_TO TGCGTATGTACTTTGAGGTATAATACACGTTAATC 1616

Ldj_tig00021666_prom_TO TGCGTATGTACTTTGAGGTATAATACACGTTAATC 2013

| **Element ①** | **Sequence** | **Score** | **Consensus match** |
| --- | --- | --- | --- |
| BRE upstream |  |  |  |
| TATA box | TATAAGAT | 0.0357 | 6/8 |
| BRE downstream | GTTATTA | NA | 3/5 |
| lnr/TSS | TCAGAC | 0.0596 | 5/6 |
| Bridge 1 | AGTAG | 0.0147 | 2/5; (4/5) |
| DPE | GAATGA | NA | 4/6 |
| Bridge 2 | ATGA | 0.0147 | 3/4 |

| **Element ② ^1^** | **Sequence** | **Score** | **Consensus match** |
| --- | --- | --- | --- |
| BRE upstream | CCACACG | NA | 5/7; Lda 4/7 |
| TATA box | TATATAAA | 1.0000 | 8/8 |
| BRE downstream | GTTAAAG | NA | 4/6 |
| lnr/TSS | TCATTC | 0.5567 | 6/6 |
| Bridge 1 | GAGGT | 0.0811 | 1/5; (5/5) |
| DPE | ACACGT | 0.2658 | 6/6 |
| Bridge 2 | ACGT | 0.0811 | 4/4 |

# Figure S6. Candidate gene annotation status across analyses.

Categories reflect all flight analyses and their groupings that featured annotated genes. Flight analysis abbreviations reflect the flight capability GWAS (FC) forewing length GWAS (FL), inbred line flight capability analysis (IL), and pupal differential gene expression analysis model terms Strain (ES) or Strain 𝗑 Day (ESD).


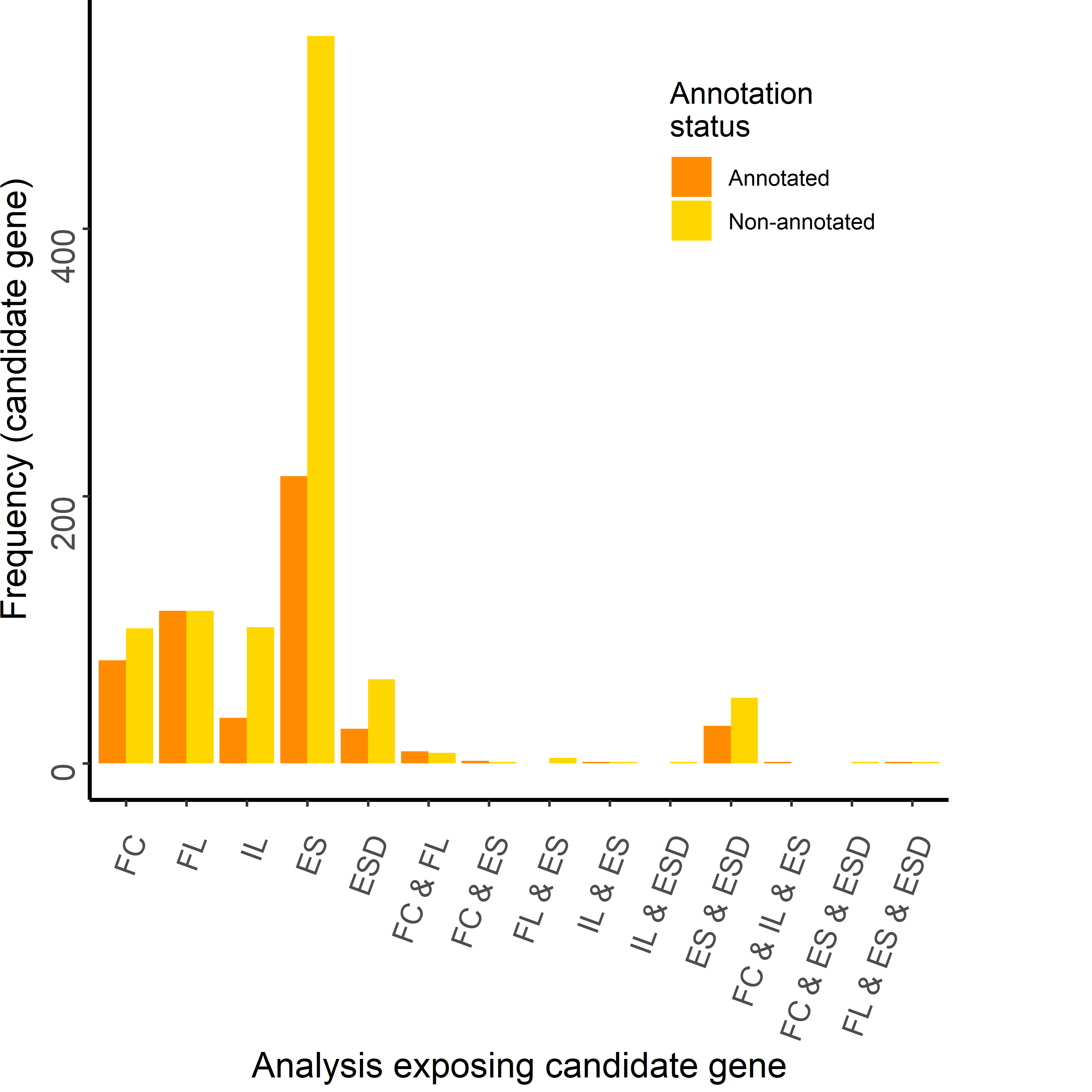


# Figure S7. Flight-relevant functional categories represented by MCL functional clusters.

Statistically significant MCL clusters (p < 0.05) were identified from among all significantly enriched GO terms (p < 0.05) within each flight analysis. The graphs sort GO terms from those clusters into manually defined flight-relevant categories. Asterisks reflect significantly strong category representation of outlier annotations from each flight analysis relative to all available annotations in the reference genome (binomial test, adjusted for multiple comparisons: * p_adj_ < 0.05, **, p_adj_ < 0.01, *** p_adj_ < 0.001).


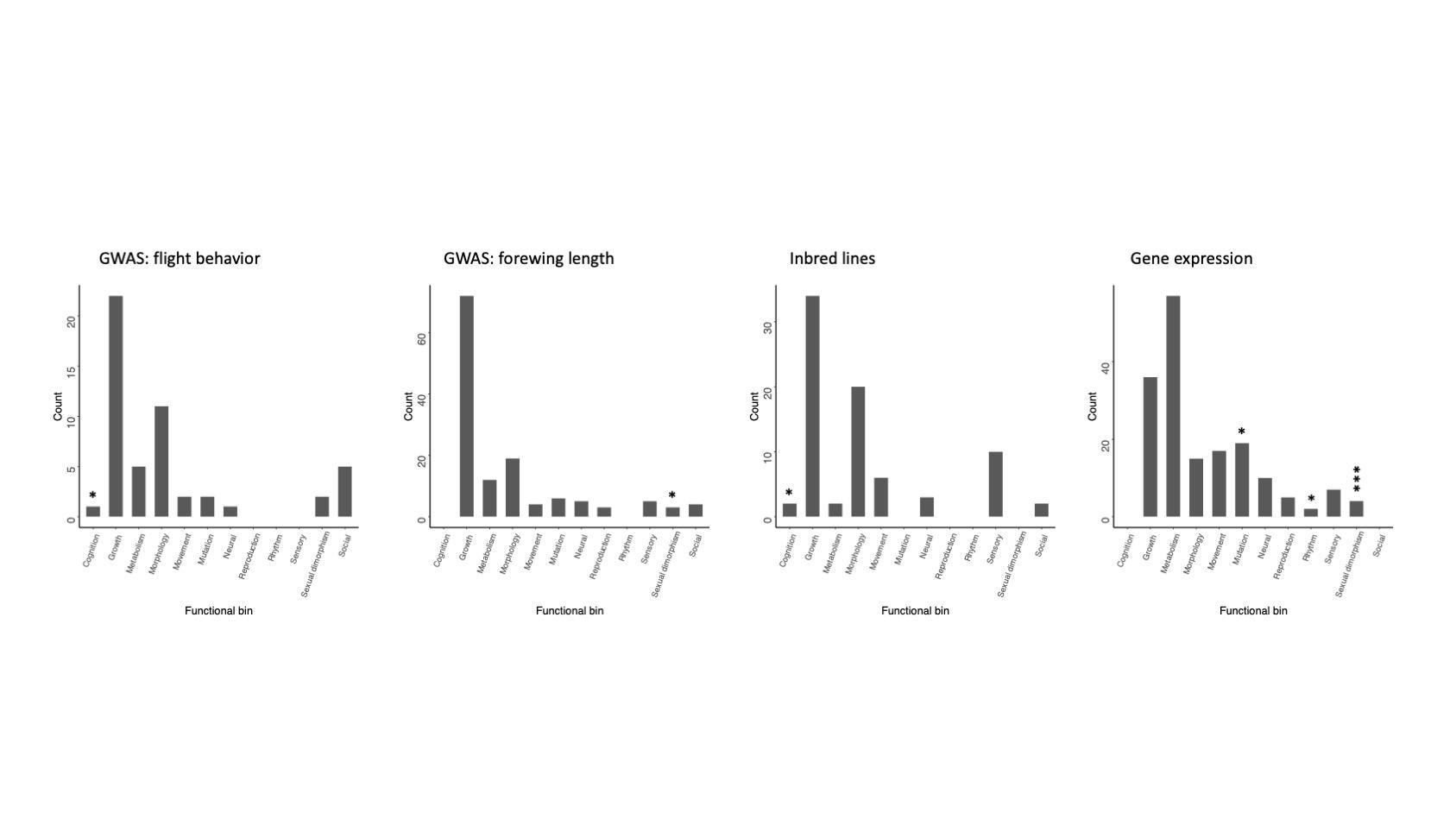


# Figure S8. Taxonomically general literature support.

Articles returned per annotated candidate gene, based on a taxonomically unrestricted search of the scientific literature (for search parameters, see Supporting Information “Literature search parameters”). Black symbols highlight genes identified as functional candidates in the insect-specific literature search. Overall, literature support was heterogeneous across candidate genes.


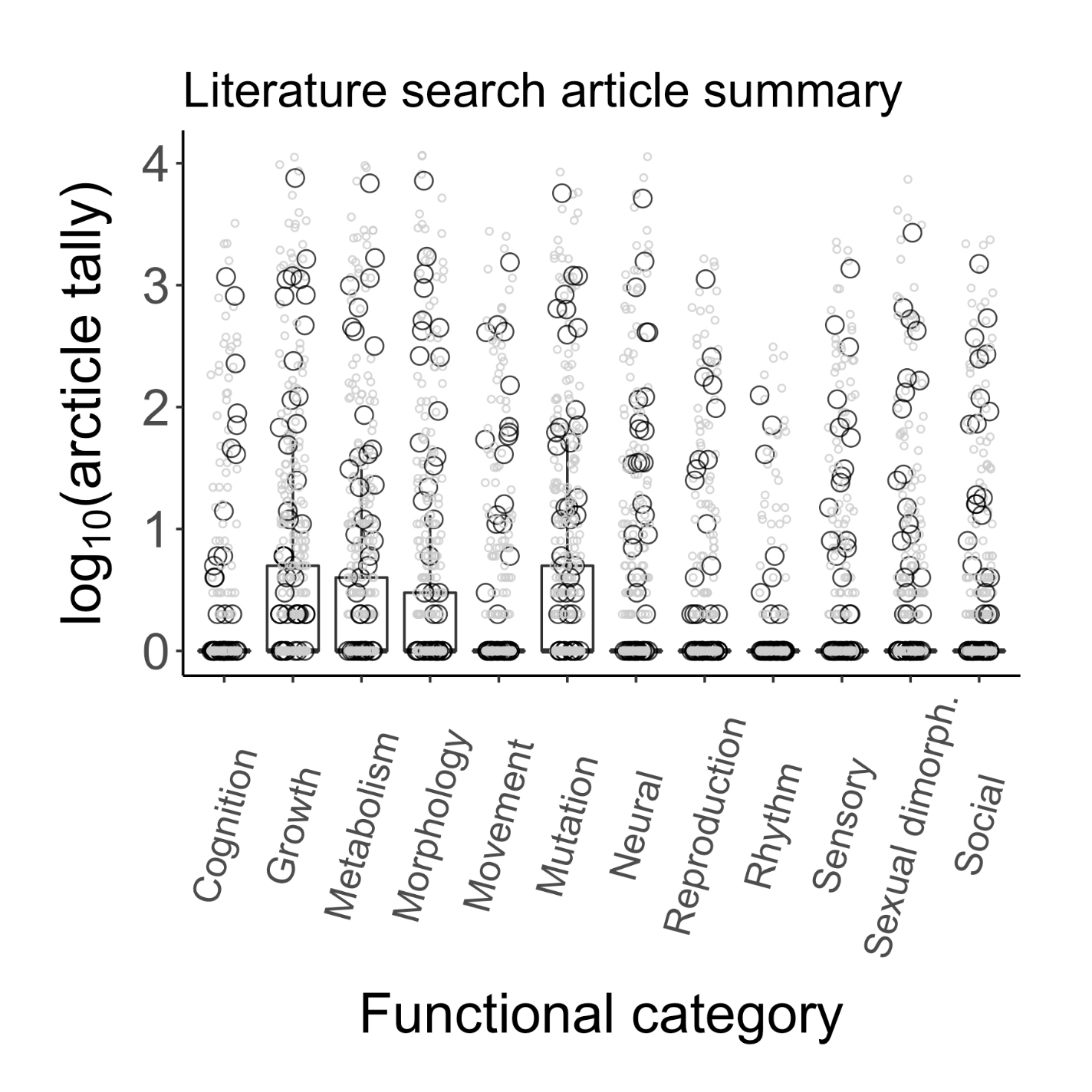

Supplement: Supplementary file 1 — Additional file 1. [file 12864_2023_9936_MOESM1_ESM.docx]
